# Bullvalene‐Containing Molecular Glasses

**DOI:** 10.1002/anie.202514797

**Published:** 2025-09-10

**Authors:** Yuzhen Wen, Christopher Hogg, Mariia Kuznetsova, Aisha N. Bismillah, Stephen J. Cowling, Paul R. McGonigal

**Affiliations:** ^1^ Department of Chemistry University of York Heslington York YO10 5DD UK; ^2^ Department of Chemistry Durham University Durham DH1 3LE UK; ^3^ Department of Chemistry University of Oxford Oxford OX1 3TA UK

**Keywords:** Amorphous, Bullvalene, Fluxionality, Glasses, Rearrangement

## Abstract

Organic molecular glasses are attractive matrices to disperse active ingredients in pharmaceuticals or electronic devices. Typically, they i) have lower glass transition temperatures than inorganic or polymeric glasses, making them easier to process, and ii) are less prone to phase segregation from other organic active materials. However, there is a dearth of functional groups that are known to induce glass formation in preference to crystallization. We have investigated the relationship between the shapeshifting isomerism of heterodisubstituted bullvalenes (BVs) and their properties as amorphous molecular glasses. Substituting a constitutionally dynamic BV unit in place of the 1,4‐phenylene motif in the molecular structures of two well‐known liquid crystal mesogens, 4‐cyano‐4′‐pentylbiphenyl and 4‐cyano‐4′‐butylbiphenyl, produces materials that readily form glasses. The properties of the two glasses are compared to analogous glasses with fixed constitutions. Using differential scanning calorimetry (DSC) and polarized optical microscopy (POM), we show that, unlike the fixed‐structure glasses, the BV‐containing molecular glasses fracture at low temperatures, which is indicative of them having larger thermal expansion coefficients. This article highlights the capability of shapeshifting building blocks to induce glass formation and to alter the physical properties of the resulting molecular materials, producing macroscopic effects that are observable by eye.

## Graphical abstract

Bullvalenes (BVs) undergo rapid pericyclic rearrangements between a network of constitutional isomers with a variety of different shapes. Their inherent covalent disorder lends them to form molecular glasses. We find that their inclusion in the structures of glass‐forming materials results in an increase in the thermal expansion coefficient.

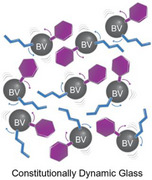

Bullvalene (BV) is a C_10_H_10_ cage that interconverts between more than a million degenerate isomers through the concerted movement of C─C bonds during rapid strain‐assisted Cope rearrangements.^[^ ^1^, ^2^, ^3^, ^4^, ^5^ ^]^ Replacing any of its H atoms with other substituents breaks the structural degeneracy, resulting in an equilibrium distribution of constitutional isomers with varying shapes and properties (Figure 1a).

**Figure 1:**
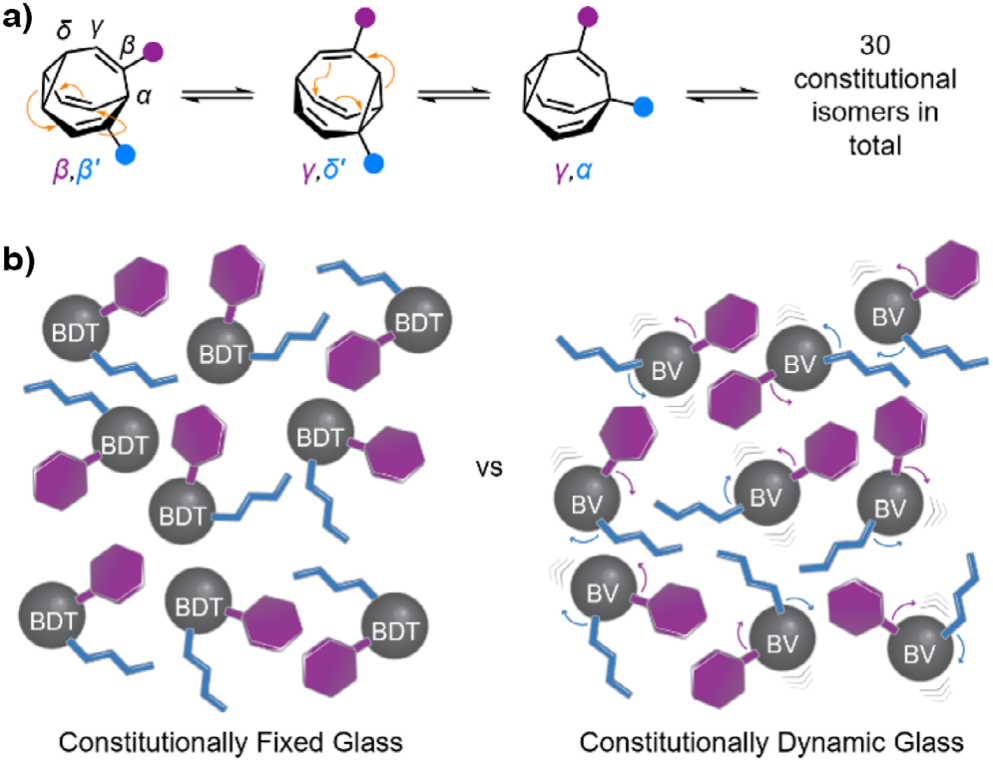
a) The shapeshifting isomerism of heterodisubstituted BVs by sequential Cope rearrangements (orange arrows). b) Schematic representation of glasses made from molecules with fixed (left) or dynamic (right) covalent structures.

The consequences of these “shapeshifting” rearrangements for solid‐state structure and properties have been investigated previously. Crystalline samples of the parent BV hydrocarbon and some structurally simple derivatives (e.g., fluorobullvalene) maintain their rapid constitutional dynamics.^[^ ^6^, ^7^, ^8^, ^9^, ^10^, ^11^, ^12^ ^]^ However, even marginally more complex BVs, such as heterodisubstituted derivatives (Figure 1a), undergo much larger shape changes during isomerization.^[^ ^13^ ^]^ Consequently, they exhibit shape‐selective crystallization dictated by packing effects in the highly ordered and rigid environment of the crystal, losing their fluxional properties and resolving to a single isomer.^[^ ^14^, ^15^, ^16^, ^17^ ^]^ Golder and coworkers have recently investigated the influence of shapeshifting rearrangements in amorphous polymeric materials. They have reported that the presence of BV units in rigid‐rod polymers modulates the polymers’ rigidity,^[^ ^18^ ^]^ whereas including BV units in cross‐linked elastomer networks dissipates energy under mechanical stimulus and increases the barrier to glass transition.^[^ ^19^ ^]^ However, the potential of small‐molecule BVs (Figure 1b) to form amorphous materials—and their resulting properties—remains unexplored.

Glasses are amorphous, metastable phases that possess the rigidity of crystalline materials while lacking long‐range positional and orientational order. Though glasses are more traditionally formed from inorganic materials (e.g., SiO_2_) or organic polymers, organic molecular glasses composed of discrete small molecules offer distinctive and attractive properties.^[^ ^20^, ^21^, ^22^ ^]^ Unlike many inorganic glasses or polymers, organic molecular glasses can often be processed conveniently as neat liquids under easily accessible conditions on account of their relatively low glass transition temperatures, *T*_g_. As solids (below their *T*_g_), their irregular structures and lack of crystallinity are attractive for use in organic electronics. They have been used in devices as host layers that effectively disperse organic active materials and dopants without being prone to phase segregation.^[^ ^23^, ^24^, ^25^, ^26^, ^27^, ^28^, ^29^, ^30^ ^]^ For similar reasons, they are also being investigated as matrices to solubilize or stabilize active pharmaceutical ingredients.^[^ ^31^, ^32^, ^33^, ^34^, ^35^, ^36^, ^37^ ^]^

Here, we report the synthesis and phase behavior of heterodisubstituted BVs **1a** and **1b** (Scheme 1), as well as their constitutionally fixed bicyclo[4.2.2]deca‐2,4,7,9‐tetraene (BDT) isomers **2a** and **2b**. We have characterized the glass transitions of these four compounds by differential scanning calorimetry (DSC) and variable‐temperature polarized optical microscopy (POM) experiments. Unlike the control materials **2a** and **2b**, the glass phases of the shapeshifting BV materials **1a** and **1b** exhibit in‐plane tensile strain‐induced fracturing as they are cooled.^[^ ^38^, ^39^, ^40^ ^]^ This phenomenon highlights how shapeshifting components can influence bulk material behavior, producing macroscopic effects that are observable by eye.

**Scheme 1:**
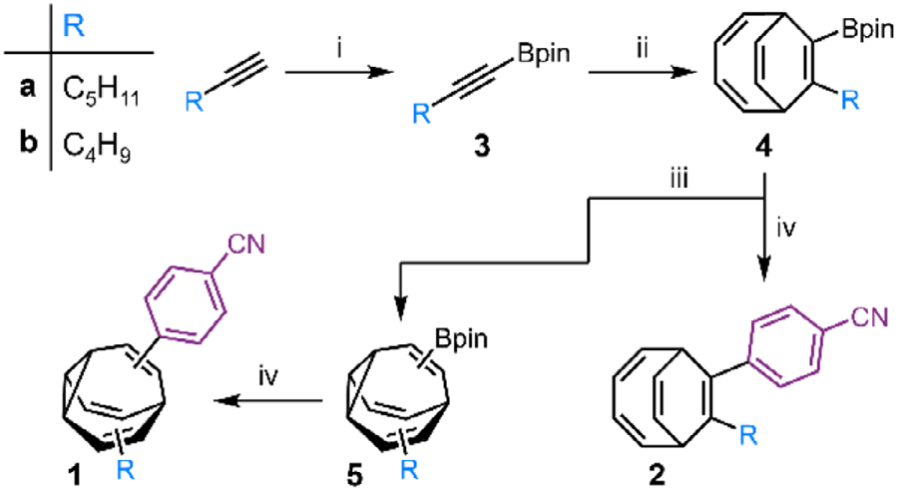
Synthesis of **1** and **2**. Reagents and conditions: i) 1. *n*‐Butyllithium (2.5 M in hexanes), Et_2_O, −78 °C, 1 h; 2. Methoxyboronic acid pinacol ester, rt, 4 h, 43% (**3a**), 31% (**3b**). ii) CoBr_2_(dppe), ZnI_2_, Zn, cyclooctatetraene, 1,2‐dichloroethane, rt, 16 h, 35% (**4a**), 29% (**4b**). iii) Thioxanthone, THF, 365 nm. iv) 4‐Bromobenzonitrile, Pd(PPh_3_)_4_, THF, H_2_O, NaOH, 60 °C, 16 h, 28% over two steps (**1a**), 12% over two steps (**1b**), 42% (**2a**), 34% (**2b**).

We targeted compounds based on 4‐cyano‐4′‐pentylbiphenyl (5CB) and 4‐cyano‐4′‐butylbiphenyl (4CB),^[^ ^41^, ^42^ ^]^ which are well‐known for their propensity to form liquid crystalline and crystalline phases, as well as glassy liquid crystals upon supercooling.^[^ ^43^ ^]^ We hypothesized that substituting the central phenylene ring of 5CB or 4CB with a BV linker, giving **1a** and **1b** (Scheme 1), respectively, would produce structures that are prone to similarly rich phase behavior. Both BVs were synthesized using Fallon's sequence of Co‐catalyzed [6+2] cycloaddition between a boronate ester alkyne and cyclooctatetraene, followed by photoisomerization and Suzuki–Miyaura coupling.^[^ ^15^, ^44^ ^]^ The static control compounds **2a** and **2b** were prepared analogously by omitting the photoisomerization step and coupling the BDT intermediate **4** to 4‐bromobenzonitrile directly (Scheme 1, see the Supporting Information for full experimental details).

We characterized **1** and **2** ^[^ ^45^ ^]^ by ^1^H and ^13^C nuclear magnetic resonance (NMR) spectroscopy (Figures S2–S41). The room‐temperature spectra of the BVs **1** display characteristically broad signals as rearrangements between isomers occur at rates comparable to the frequency differences between peaks. Typically,^[^ ^17^ ^]^ BV rearrangements have Gibbs energies of activation of ∼55 kJ·mol^−1^, corresponding to half‐lives, *t_½_*, of ∼500 µs at room temperature. Acquiring NMR spectra at −63 °C (Figure 2a) slows the rearrangements to *t_½_* ∼10 s, making the resonances for the most prevalent isomers visible as sharp signals.

**Figure 2:**
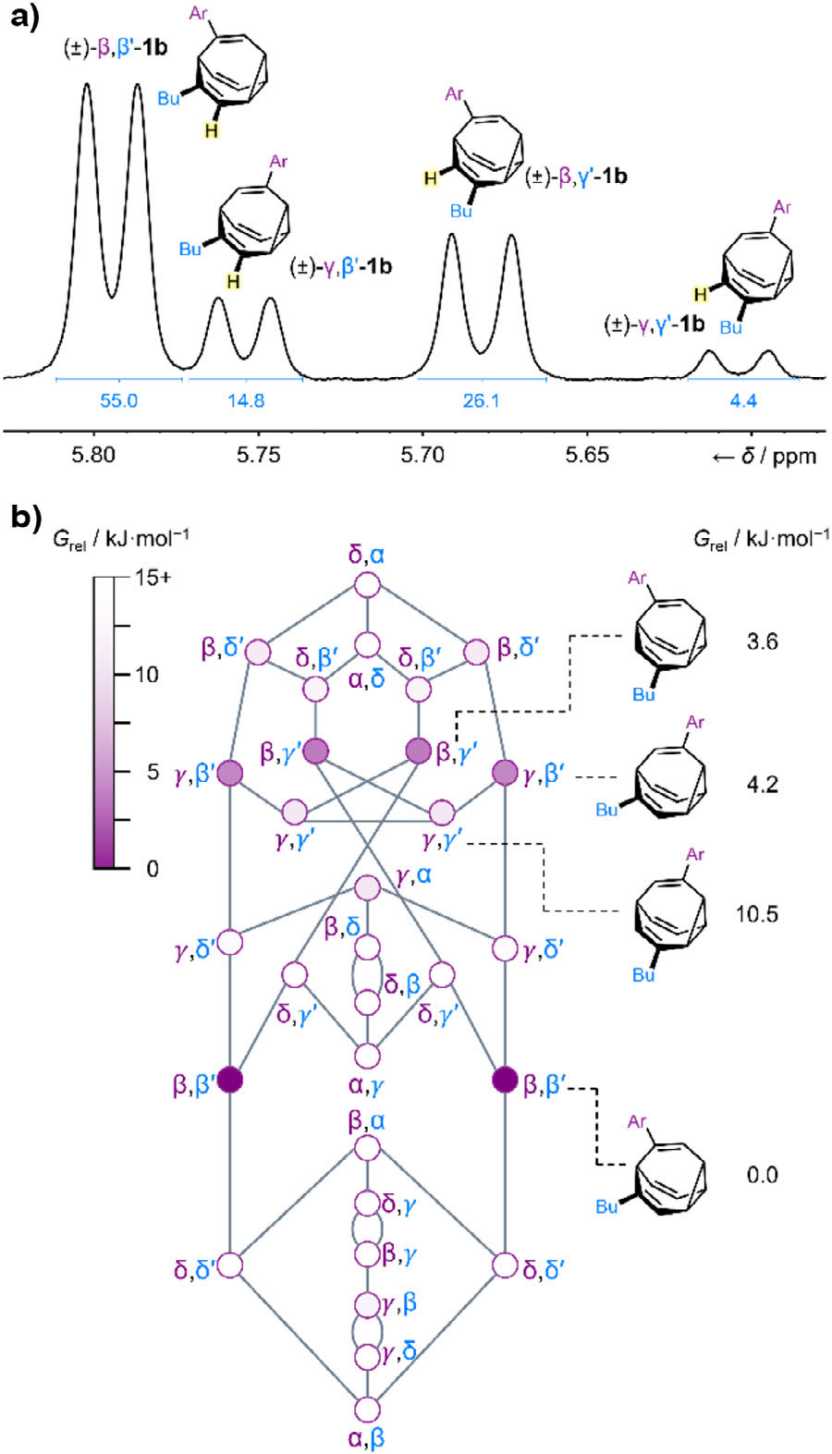
a) Partial ^1^H NMR spectrum (500 MHz, −63 °C, CDCl_3_) of **1b** showing the relative proportions of the eight most populated constitutional isomers. b) Network diagram for the isomerization of **1b** showing the relative Gibbs energies predicted by DFT modeling at the PBE0‐D3/def2‐SV(P) level of theory.^[^ ^46^, ^47^, ^48^, ^49^, ^50^, ^51^, ^52^, ^53^, ^54^, ^55^, ^56^, ^57^, ^58^, ^59^, ^60^ ^]^ Ar = C_6_H_4_CN.

Using *bullviso* to generate initial isomer geometries,^[^ ^13^ ^]^ we also performed density functional theory (DFT) calculations^[^ ^46^, ^47^, ^48^, ^49^, ^50^, ^51^, ^52^, ^53^, ^54^, ^55^, ^56^, ^57^, ^58^, ^59^, ^60^ ^]^ to model the relative Gibbs energies, *G*_rel_, of the **1b** isomers (Figure 2b).^[^ ^61^ ^]^ The shapeshifting equilibrium includes a total of 30 constitutional isomers, shown as nodes in the network diagram (Figure 2b). These isomers are linked through a series of Cope rearrangements, represented by connecting lines. The 12 achiral isomers are arranged vertically along the center of the diagram, and nine pairs of enantiomers are on either side. Our DFT calculations indicate that the lowest energy isomers of **1b** are the four pairs of enantiomers with the β,β″‐, β,γ″‐, γ,β″‐, and γ,γ″‐substitution patterns. Based on their computed energies, the Boltzmann distribution at 25 °C (Table S1) is predicted to include these isomers in a 70:16:8:1 ratio, which is relatively close to the 53:25:17:5 ratio observed experimentally by ^1^H NMR spectroscopy at −63 °C for **1a** and the 55:26:15:4 ratio observed for **1b** (Figure 2a). Therefore, the shapeshifting mixture is composed predominantly of eight isomers, (±)‐β,β″‐**1**, (±)‐β,γ″‐**1**, (±)‐γ,β″‐**1**, and (±)‐γ,γ″‐**1**, while other isomers are each present in the mixture in smaller amounts, predicted to be <0.5% (Table S1).^[^ ^62^ ^]^ These eight BV isomers orient their constituent alkyl and aryl groups with different dihedral angles (0° or 30°) and plane angles (27°–54°) while separating them by a range of distances (∼2.5 or ∼3.2 Å),^[^ ^13^ ^]^ altering the overall volumes, shapes, and dipole moments of the molecules. The BDT control compounds **2** place attached functional groups slightly closer together (1.34 Å) than the major BV isomers (Figure S44), but the dihedral angle (0°) and plane angles (53.9°) are almost identical to those of the predominant β,β'‐isomer of **1**, making it a geometrically similar, yet constitutionally fixed, structure to use as a comparison.

We identified phase transitions by performing DSC analyses of neat samples of **1** and **2** (Figures 3 and S50–S53; Table 1). All four compounds were cooled to −90 °C, then warmed to room temperature at a rate of 10 K·min^−1^. We observed step changes characteristic of a glass transition^[^ ^63^, ^64^, ^65^, ^66^, ^67^ ^]^ in the DSC curves of all four compounds. We also scanned at 5 and 1 K·min^−1^ to confirm the lack of peaks for crystallization at slower rates. The three scans were performed sequentially, showing that the glass formation and melting are reversible. Indicative *T*_g_ midpoint values are given in Table 1, and a full list of temperature ranges, midpoints of *T_g_*, and changes of specific heat capacity at constant pressure, Δ*c_p_*, are given in Table S2. The materials all display *T*_g_ midpoint values in a similar range from −26 °C to −37 °C and Δ*c_p_* values of ∼0.3 J·g^−1^·K^−1^ on cooling or ∼0.5 J·g^−1^·K^−1^ on heating. Some variations in *T*_g_ and Δ*c_p_* values are observed depending on the scan rate (Table S2), which is a common feature of glass transitions.^[^ ^63^, ^64^, ^65^, ^66^, ^67^ ^]^ As Δ*c_p_* is correlated to the change in configurational entropy between the liquid and glass states, especially large values would be expected if the isomer populations underwent significant changes during the glass transitions. Instead, the similarity of the measured Δ*c_p_* values for the shapeshifting mixture **1** and the static analogs **2** suggests that, in contrast to the crystallization of disubstituted BVs,^[^ ^13^ ^]^ the isomer populations are unperturbed by undergoing phase transition to a glass. Solid‐state NMR (ssNMR) spectra acquired across the glass transition (Figure S42) are also consistent with the isomer distribution remaining the same.

**Figure 3:**
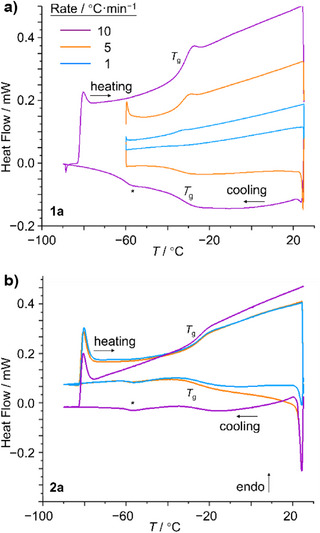
DSC traces of a) BV **1a** and b) BDT **2a**. Glass transitions upon cooling and heating are labeled as *T*_g_. An apparent peak caused by a change in intracooler power needed for the instrument to reach below −60 °C is labeled with an asterisk.

**Table 1:** Midpoint *T*_g_ values for **1** and **2**.^a)^

	*T*_g_/°C		Δ*c_p_*/J·g^−1^·K^−1^	
Compound	Cooling	Heating	Cooling	Heating
**1a**	−35	−34	0.32	0.42
**1b**	−30	−28	0.21	0.59
**2a**	−28	−26	0.28	0.54
**2b**	−26	−37	0.30	0.46

^a)^ All values were obtained using a 5 K·min^−1^ cooling and heating rate.

We used POM to observe changes in the appearance of free‐standing samples of the materials on SiO_2_ glass slides during cooling and heating (Figures 4 and S45–S48). Consistent with the formation of glass phases rather than liquid crystalline or crystalline phases, there is no evidence of birefringence or X‐ray diffraction (Figure S54) at low temperature. Unexpectedly, however, upon cooling below *T_g_*, the microscope images show that the BV glass films fracture (Figure 4a), while the BDT glasses do not (Figure 4b). Compound **1a** developed its first fracture line as the glass formed at −36 °C (Figure 4a). As the temperature is decreased further, the number of fractures increases, and each becomes more pronounced, eventually shattering the glass into millimeter‐sized pieces that then delaminate from the surface at −73 °C. Warming the sample above *T_g_* reforms the liquid, allowing the material to flow and become homogeneous (Figure 4a). The temperature cycle can be repeated, reliably inducing the same effect each time (Figure S49). BV **1b** exhibits a similar fracturing phenomenon, albeit with less pronounced fractures and with an onset at a lower temperature of approximately −75 °C (Figure S46). These fracturing and delamination phenomena have been established by Yu as hallmarks of organic glass films having substantially larger thermal expansion coefficients than the underlying substrates on which they are held.^[^ ^38^, ^39^, ^40^ ^]^ The mismatch in thermal expansion coefficients causes a buildup of in‐plane tensile strain in the organic layer as it contracts, which is released by fracture formation. Further cooling of the organic glass gives the appearance of circular fractures as the shards of glass contract further, causing their edges to curl up.

**Figure 4:**
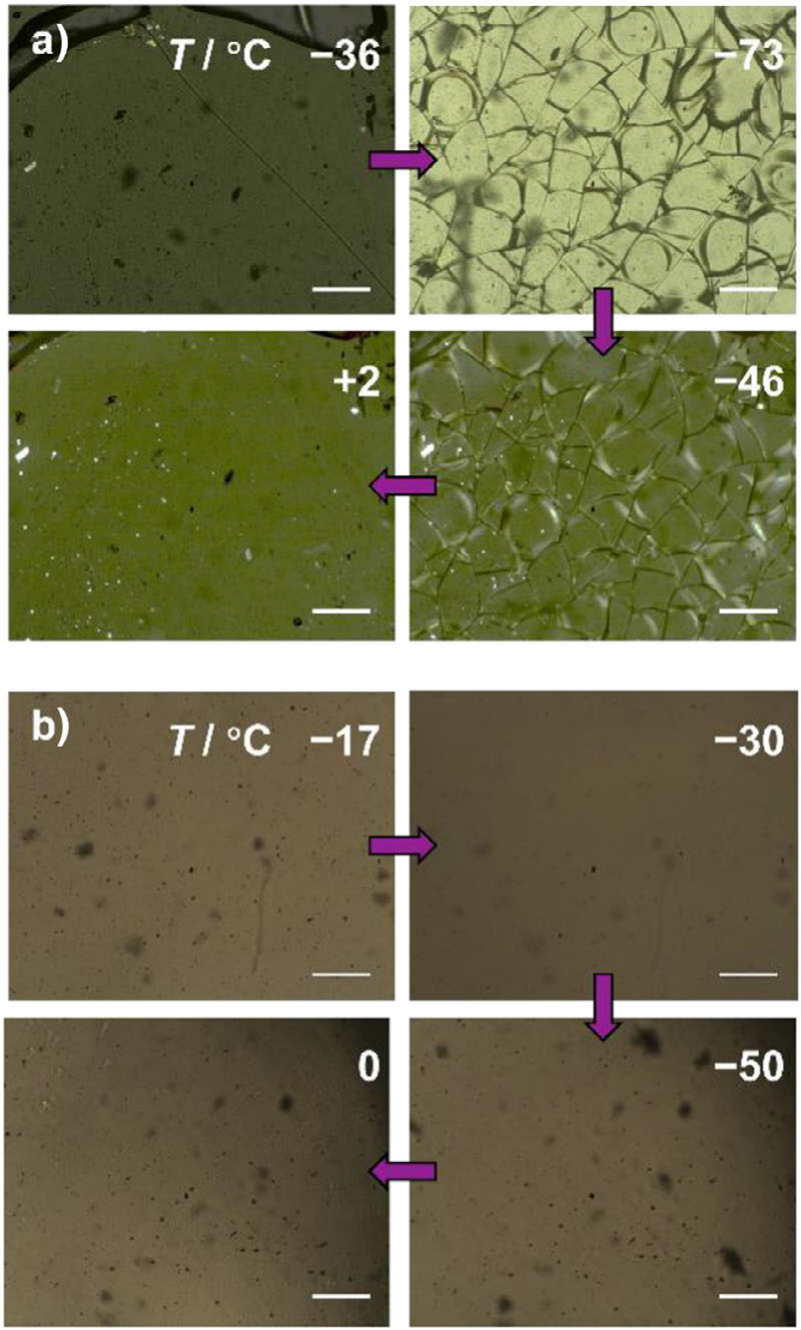
Comparison of the POM images of a) BV **1a** and b) BDT **2a** upon cooling below *T*_g_ (showing fracturing for **1a** but not **2a**), then warming above *T*_g_. Scale bars = 0.3 mm.

The absence of this fracturing phenomenon for the BDT analogs with fixed covalent structures, **2a** (Figure 4b) and **2b** (Figure S48), indicates that their thermal expansion coefficients are more closely matched to the SiO_2_ substrate. Therefore, we conclude that the subtle change in bonding from the fixed BDT core to a shapeshifting BV core, and the consequent introduction of a dynamic covalent equilibrium, alters the thermal expansion properties of the resulting molecular glasses.

In summary, we have prepared BVs **1a** and **1b**, which each access 30 constitutional isomers by shapeshifting rearrangements of their covalent structures. NMR spectroscopy and DFT modeling show that eight of these isomers are present in significant quantities at equilibrium. Cooling neat samples of the BV materials to around −40 °C produces organic molecular glasses. Evidently, replacing the 1,4‐phenylene units of 4CB and 5CB with the spherical,^[^ ^13^ ^]^ fluxional BV unit produces low‐molecular‐weight materials that are prone to forming amorphous solids. DSC and ssNMR analyses indicate that the mixture of constitutional isomers present in the liquid state persists in the glass. Therefore, the covalent structural disorder that is inherent to BV materials is likely to favor glass formation over crystallization. By comparing the shapeshifting materials to constitutionally fixed isomers **2a** and **2b** that also form glasses, we have found that the presence of the BV unit also alters the thermomechanical properties, resulting in the glasses shattering upon cooling. Shapeshifting building blocks can be considered in the design of molecular materials to impart novel physical properties and stimuli‐responsive characteristics.

## Supporting Information

General experimental details, synthetic procedures, microscope images, calorimetry data, NMR spectra, and DFT‐modeled geometries. The authors have cited additional references within the Supporting Information.^[^ ^68^, ^69^, ^70^ ^]^

## Conflict of Interests

The authors declare no conflict of interest.

## Supporting information



Supporting Information

## Data Availability

The data that support the findings of this study are available in the Supporting Information of this article.
