# Supplementary material for: Bullvalene‐Containing Molecular Glasses
Source: Angew Chem Int Ed Engl. 2025 Sep 10;64(43):e202514797. doi: 10.1002/anie.202514797 (PMC12535393; doi:10.1002/anie.202514797)
Supplement: Supplementary file 1 — Supporting Information [file ANIE-64-e202514797-s001.docx]

**Bullvalene-Containing Molecular Glasses**

Yuzhen Wen,^a,b^ Christopher Hogg,^a,c^ Mariia Kuznetsova,^a,c^ Aisha N. Bismillah,^a^ Stephen J. Cowling ^a^ and Paul R. McGonigal*^a,c^

^a^ Department of Chemistry, University of York, Heslington, York, YO10 5DD (UK)

^b^ Department of Chemistry, Durham University, Lower Mountjoy, Stockton Road, Durham, DH1 3LE (UK)

^c^ Department of Chemistry, University of Oxford, Mansfield Road, Oxford, OX1 3TA (UK)

E-mail: paul.mcgonigal@chem.ox.ac.uk

**Table of Contents**

[1. General Methods 1](#_Toc206153351)

[2. Nomenclature and Labelling System of Bullvalene **1** 2](#_Toc206153352)

[3. Synthetic Procedures 3](#_Toc206153353)

[4. ^1^H and ^13^C NMR Spectroscopic Characterisation of Synthesised Compounds 9](#_Toc206153354)

[5. Structural Assignment of Compound **1**, **2** and **4** by 2D NMR 26](#_Toc206153355)

[6. Solid-State NMR Spectroscopy 46](#_Toc206153356)

[7. Computational modelling 48](#_Toc206153357)

[8. Polarised Microscopy 52](#_Toc206153358)

[9. Differential Scanning Calorimetry 55](#_Toc206153359)

[10. X-ray Diffraction 60](#_Toc206153360)

Supporting Information

1. General Methods

All reagents were purchased from commercial suppliers (Sigma-Aldrich, Acros Organics, or Alfa Aesar) and used without further purification. Nuclear magnetic resonance (NMR) spectra were recorded using a Bruker Advance (III)-400 (^1^H 400.130 MHz and ^13^C 100.613 MHz) and Varian Inova-500 (^1^H 500.130 MHz and ^13^C 125.758 MHz). Chemical shifts (*δ*) are reported in parts per million (ppm) relative to the signals corresponding to residual nondeuterated solvents [CDCl_3_: *δ* = 7.26 or 77.2]. Coupling constants (*J*) are reported in Hertz (Hz). NMR spectra were processed using MestReNova version 14. Solid-state NMR (ssNMR) spectra were recorded using a Bruker Avance III HD spectrometer and a 4.0 mm (rotor outside diameter.) magic-angle spinning probe at a sample spin-rate of 4 kHz and a calibrated sample temperature. ^13^C spectra were recorded at 100.63 MHz. The spectra were obtained using cross-polarisation total sideband suppression (CPTOSS). The recycle delay and cross polarisation contact time were optimised at 2 s and 1 ms respectively. Spectral referencing was with respect to an external sample of neat tetramethylsilane (carried out by setting the high-frequency signal from adamantane to 38.5 ppm). Data are reported as follows: chemical shift; multiplicity; coupling constants; integral and assignment. Low-resolution ASAP-MS was performed using a Waters Xevo QTOF equipped with an Atmospheric Solids Analysis Probe (ASAP). High resolution electrospray (HR-ESI) and ASAP (HR-ASAP) mass spectra were measured using a Waters LCT Premier XE high resolution, accurate mass UPLC ES MS (also with ASAP ion source). Photoirradiation reactions were performed with an array of ten 365 nm light-emitting diodes (EvoluChem LED 365PF, 18 W, 190 mW·cm^−2^ irradiance) in a HepatoChem PhotoRedOx Box. DSC experiments were conducted using METTLER instrument equipped with TC100 intracooler. Samples were loaded using 20 μL aluminium light crucibles. Operations and data extractions were done using Mettler Star software V.18. The DSC diagrams are reformatted using OriginPro 2022. Polarised optical microscopy images were obtained using an Olympus BX50 Optical Microscope equipped with a Linkam Scientific LTS350 heating stage, Linkam LNP2 cooling pump, and Linkam TMS92 controller and samples were held on Academy microscope slides (76×26 mm, 1.0–1.2 mm thickness, cat. no. N/A143).

2. Nomenclature and Labelling System of Bullvalene 1

To maintain consistency and facilitate comparison, the nomenclature used for bullvalene isomers follows the same labelling conventions as described in our previous work.^[13]^ Specifically, we adopt the relative positional labelling system using Greek letter locants (α–δ) to denote distinct carbon sites on the bullvalene scaffold (Figure S1a), allowing isomers to be labelled according to the positions of substituents (Figure S1b). In this work, both bullvalene **1a** and **1b** bear an aryl and an alkyl chain substituent. For clarity and consistency, the aryl substituent is always listed first when using Greek letter positional labels (e.g. for isomer β,γ', “β” refers to the aryl position), reflecting its higher CIP priority. Although several substitution patterns give rise to enantiomeric pairs, the enantiomers are identical by NMR, so are labelled as a single species for the purposes of NMR assignment. When assigning numeric labels to the carbon atoms, we define the ethylene bridge bearing the aryl group as C_1_–C_3_, the one bearing the alkyl chain as C_5_–C_7_, the vacant one as C_8_–C_10_ and the bridgehead position as C_4_ (Figure S1c).


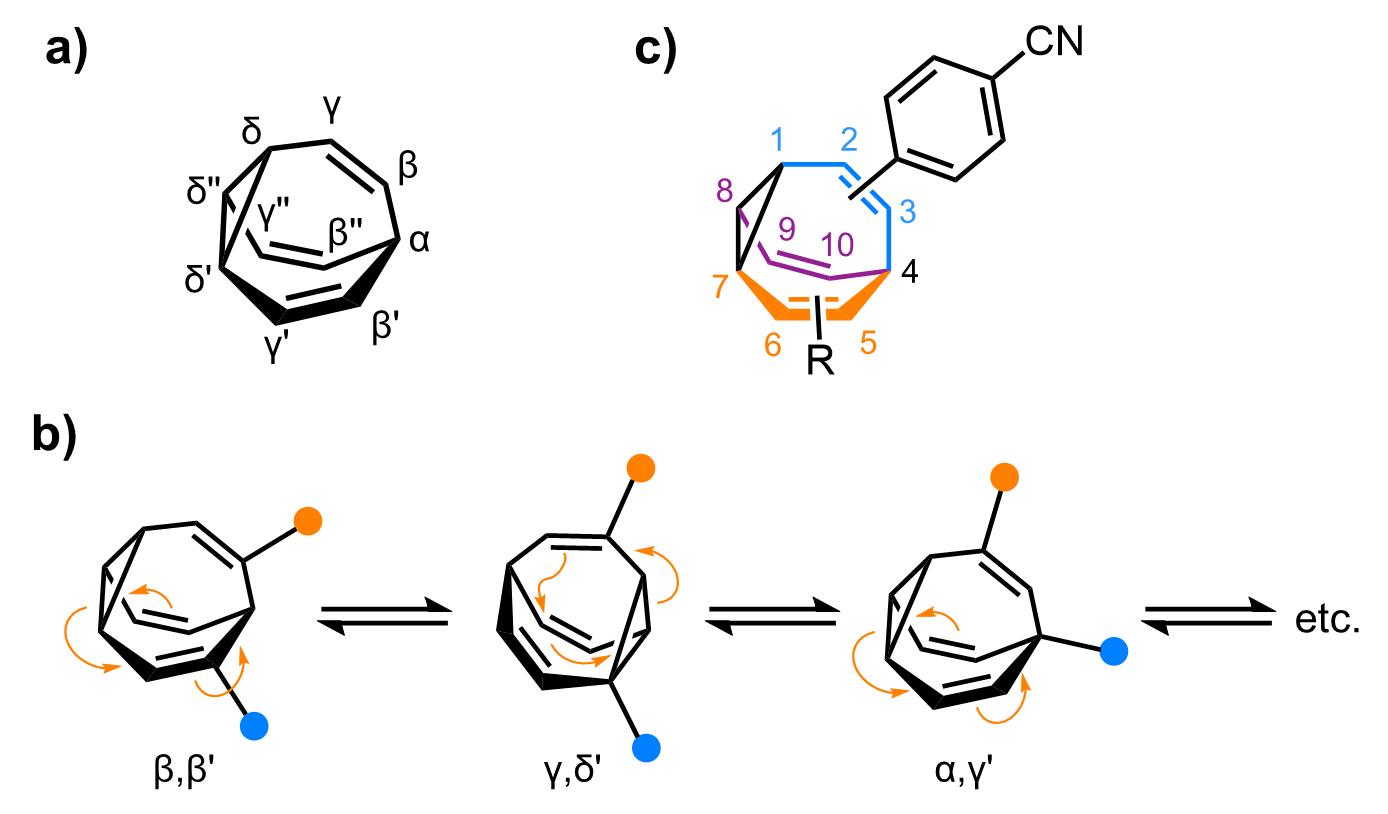


**Figure S1**. a) The relative positional labels of BV using Greek letters; b) The partial isomer network of a heterodisubstituted BV showing the positional exchange arising from two sequential Cope rearrangement steps; c) Schematic representation of the atom numbering convention used for BV **1**.

3. Synthetic Procedures


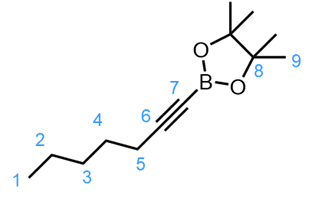
**2-(Hept-1-yn-1-yl)-4,4,5,5-tetramethyl-1,3,2-dioxaborolane (3a):** 1-Heptyne (3.2 mL, 24 mmol) in anhydrous diethyl ether (60 mL) was cooled to −78 °C under a N_2_ atmosphere. n-Butyllithium (9.8 mL, 2.5 M in hexanes, 24 mmol) was added dropwise, followed by stirring at −78 °C for 1 h. 4,4,5,5-Tetramethyl-2-(1-methylethoxy)-1,3,2-dioxaborolane (4.1 mL, 20 mmol) in anhydrous diethyl ether (60 mL) was then added. The reaction mixture was stirred at rt for 4 h before being cooled to −78 °C, followed by quenching with HCl (7.5 mL, 4.0 M in dioxane, 30 mmol) and stirring at rt for an additional 1 h. The solvent was removed under reduced pressure and the crude product was purified by column chromatography (Teledyne Isco Combi Flash Rf+ system, 80 g Al_2_O_3_, n-hexane‒EtOAc, 0–100% gradient elution) to give the title compound as a yellow oil (1.90 g, 8.56 mmol, 43%). **^1^H NMR** (400 MHz, CDCl_3_, 298 K): δ 2.23 (t, *J* = 7.2 Hz, 2H, H_5_), 1.58 – 1.46 (m, 2H, H_4_), 1.36 – 1.30 (m, 2H, H_3_), 1.28 – 1.25 (m, 2H, H_2_), 1.23 (s, 12H, H_9_), 0.87 (t, *J* = 7.1 Hz, 3H, H_1_). Characterisation data are consistent with those reported previously.^[68]^

**
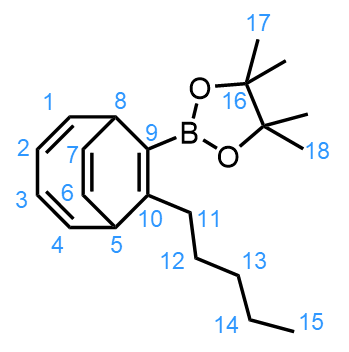
**

**(±)-4,4,5,5-Tetramethyl-2-((2*Z*,4*Z*)-8-pentylbicyclo[4.2.2] deca-2,4,7,9-tetraen-7-yl)-1,3,2-dioxaborolane (4a):** An oven-dried Schlenk tube containing anhydrous ZnI_2_ (256 mg, 800 μmol) was heated at 280 °C under high vacuum for 5 min. After cooling to rt and flushing with N_2_, CoBr_2_(dppe) (248 mg, 400 μmol), Zn (80 mg, 1.2 mmol), and anhydrous 1,2-dichloroethane (4 mL) were added and the reaction mixture was then deaerated by three freeze-pump-thaw cycles with N_2_. Cyclooctatetraene (416 mg, 4.00 mmol) was added into the reaction mixture. **3a** (904 mg, 4.35 mmol in 4 mL of anhydrous 1,2-dichloroethane, deaerated) was slowly added using a syringe pump over 6 h. The reaction was stirred at rt in a H_2_O bath for 16 h. After completion, the suspension was filtered through a short pad of SiO_2_ (5 g) eluting with Et_2_O. The solvent was removed under reduced pressure, and the crude product was purified by column chromatography (Teledyne Isco CombiFlash Rf+ system, 24 g SiO_2_, n-hexane‒EtOAc, 0–100% gradient elution) to give the title compound as a yellow oil (458 mg, 1.40 mmol, 35%). **^1^H NMR** (400 MHz, CDCl_3_, 298 K): δ 6.33 – 6.23 (m,1H, H_2_), 6.15 – 6.04 (m, 1H, 1H, H_3_), 5.75 – 5.55 (m, 4H, H_1/4/6/7_), 3.49 – 3.37 (m, 1H, H_8_), 3.35 – 3.24 (m, 1H, H_5_), 2.53 – 2.36 (m, 2H, H_11_), 1.45 – 1.26 (m, 6H, H_12/13/14_), 1.24 (d, *J* = 4.7 Hz, 12H, H_17/18_), 0.87 (t, *J* = 7.1 Hz, 3H, H_15_). **^13^C NMR** (151 MHz, CDCl_3_, 298 K): δ 151.0 (C_10_), 143.1 (C_2_), 140.4 (C_3_), 124.8 (C_7_), 123.3 (C_6_), 122.2 (C_1_), 121.3 (C_4_), 82.6 (C_16_), 40.9 (C_5_), 37.0 (C_8_), 34.2 (C_11_), 31.6 (C_13_), 30.5 (C_12_), 25.0 (C_17_), 24.4 (C_18_), 22.3 (C_14_), 14.0 (C_15_). **HR ESI MS:** m/z = 327.2480 [M+H]^+^, calculated for C_21_H_32_^11^BO_2_^+^: 327.2490

**
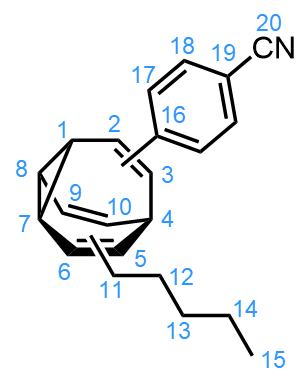
**

**4‑Cyanophenyl-pentyl-BV (1a)**: **4a** (281 mg, 860 μmol) and thioxanthone (3.6 mg, 19 μmol) were dissolved in anhydrous THF (4 mL). The reaction mixture was irradiated with a 365 nm LED lamp while being kept at 25 °C by immersion in a H_2_O bath for 24 h. The solvent was removed under reduced pressure and the crude product was purified by column chromatography (Teledyne Isco CombiFlash Rf+ system, 80 g Al_2_O_3_, n-hexane‒CH_2_Cl_2_ 0–100% gradient elution) to give compound **5a** as a colourless oil (88 mg, crude). The broad peaks of the ^1^H NMR spectrum indicated the successful formation of the corresponding BV. The product was then taken into the next step without further purification: **5a** (88 mg, crude), 4‑bromobenzonitrile (55 mg, 0.30 mmol), and Pd(PPh_3_)_4_ (69 mg, 60 μmol) were dissolved in a mixture of THF (1.5 mL) and 10% aqueous NaOH solution (0.6 mL). The mixture was deaerated by three freeze-pump-thaw cycles and stirred at 60 °C for 16 h. The reaction mixture was diluted with diethyl ether (5 mL) and dried over MgSO_4_. The solvent was removed under reduced pressure and the crude product was purified by column chromatography (Teledyne Isco Combi Flash Rf+ system, 12 g SiO_2_, hexanes−CH_2_Cl_2_, 0–100% gradient elution) to give the title compound as a yellow oil (71 mg, 0.24 mmol, 28%). **^1^H NMR** (499 MHz, CDCl_3_, 210 K) δ 7.66 – 7.58 (m, 2H, H_18_), 7.47 (m, 0.45H, H_17_, γ,β'+γ,γ'), 7.40 (m, 1.54H, H_17_, β,β'+β,γ'), 6.28 – 6.19 (m, 0.78H, H_2_, β,β'+β,γ'), 6.13 (m, 0.22H, H_3_, γ,β'+γ,γ'), 6.07 – 5.81 (m, 2H, H_9/10_), 5.77 (d, *J* = 7.7 Hz, 0.54H, H_6_, β,β'), 5.73 (d, *J* = 8.0 Hz, 0.16H, H_6_, γ,β'), 5.66 (d, *J* = 9.0 Hz, 0.24H, H_5_, β,γ'), 5.58 (d, *J* = 8.8 Hz, 0.05H, H_5_, γ,γ'), 2.98 (m, 0.25H, H_4_, β,γ'), 2.86 (d, *J* = 8.7 Hz, 0.43H, H_4_, β,β'), 2.72 - 2.57 (m, 0.32H, H_4_, γ,β'+γ,γ'), 2.53 – 2.32 (m, 3H, H_1/7/8_), 2.13 – 1.99 (m, 2H, H_11_), 1.50 – 1.01 (m, 6H, H_12/13/14_), 0.84 (m, 1.80H, H_15_, β,β'), 0.77 (m, 1.20H, H_15_, β,γ'+γ,β'+γ,γ'). **^13^C NMR** (126 MHz, CDCl_3_, 210 K) δ 149.5 (C_20_, γ,β'), 147.8 (C_20_, β,β'+β,γ'+γ,γ'), 142.3 (C_5_, γ,β'), 141.5 (C_5_, β,β'), 140.3 (C_6_, β,γ'), 139.1 (C_2_, β,γ'), 138.6 (C_2_, β,β'), 137.2 (C_3_, γ,β'), 136.8 (C_3_, γ,γ’), 132.6 (C_18_), 128.0 (C_9_), 127.9 (C_2_, β,β’), 127.5 (C_2_, β,γ'), 127.2 (C_16_, γ,β'+γ,γ'/C_10_), 126.8 (C_3_, γ,β'+γ,γ'), 126.5 (C_16_, β,β’), 126.5 (C_16_, β,γ'), 120.5 (C_6_, β,β'), 119.9 (C_19_), 119.6 (C_6_, γ,β'), 119.0 (C_5_, γ,γ'), 118.6 (C_5_, β,γ'), 109.5 (C_16_, γ,β'), 109.4 (C_16_, γ,γ'), 109.2 (C_16_, β,β'), 109.1 (C_16_, β,γ'), 41.2 (C_11_, β,γ'), 40.4 (C_11_, γ,β'), 40.2 (C_11_, β,β'), 38.9 (C_4_, β,β'), 34.8 (C_4_, γ,β'), 34.3 (C_4_, β,γ'), 31.7 (C_13_, β,γ'+γ,β'+γ,γ'), 31.3 (C_13_, β,β'), 29.1 (C_12_, γ,β'), 28.3 (C_12_, β,β'), 28.2 (C_12_, γ,β'), 24.3 (C_1_ or C_7_ or C_8_, β,γ'), 23.0 (C_14_, β,γ'+γ,β'+γ,γ'), 22.9 (C_14_, β,β'), 22.3 (C_1_ or C_7_ or C_8_, γ,β'), 21.4 (C_1_ or C_7_ or C_8_, β,β'), 21.4 (C_1_ or C_7_ or C_8_, β,γ'), 21.3 (C_1_ or C_7_ or C_8_, β,β'), 21.1 (C_1_ or C_7_ or C_8_, β,β'), 21.0 (C_1_ or C_7_ or C_8_, β,γ'), 20.1 (C_1_ or C_7_ or C_8_, γ,β'), 19.7 (C_1_ or C_7_ or C_8_, γ,β'), 14.7 (C_15_, β,γ'+γ,β'+γ,γ'), 14.6 (C_15_, β,β'). **HR ASAP MS**: m/z = 302.1898 [M+H]^+^, calculated for C_22_H_24_N^+^: 302.1903.

**
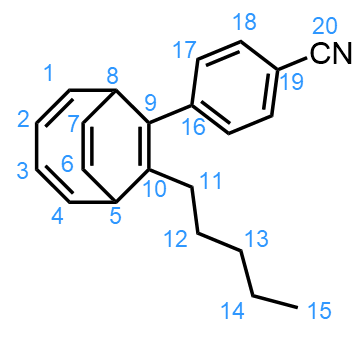
**

**(±)-4-((2*Z*,4*Z*)-8-pentylbicyclo[4.2.2]deca-2,4,7,9-tetraen-7-yl)benzonitrile (2a)**: **4a** (140 mg, 0.43 mmol), 4‑bromobenzonitrile (83 mg, 0.46 mmol), and Pd(PPh_3_)_4_ (23 mg, 20 μmol) were dissolved in the mixture of THF (2 mL) and 10% aqueous NaOH solution (0.6 mL). The mixture was deaerated by three freeze-pump-thaw cycles and stirred at 60 °C for 16 h. The reaction mixture was diluted with diethyl ether and dried over MgSO_4_. The solvent was removed under reduced pressure and the crude product was purified by column chromatography (Teledyne Isco Combi Flash Rf+ system, 12 g SiO_2_, hexanes–CH_2_Cl_2_, 0–100% elution) to give the title compound as a yellow oil (54 mg, 0.18 mmol, 42%). **^1^H NMR** (400 MHz, CDCl_3_, 298 K): δ 7.61 – 7.55 (m, 2H, H_18_), 7.29 – 7.20 (m, 2H, H_17_), 6.33 – 6.15 (m, 2H, H_2/3_), 5.88 – 5.67 (m, 4H, H_1/4/6/7_), 3.33 (m, 2H, H_5/8_), 2.10 – 2.00 (m, 1H, H_11E_), 1.93 – 1.82 (m, 1H, H_11A_), 1.46 – 1.28 (m, 2H, H_12_), 1.20 – 1.05 (m, 4H, H_13/14_), 0.81 (t, *J* = 7.1 Hz, 3H, H_15_). **^13^C NMR** (101 MHz, CDCl_3_, 298 K): δ 146.9 (C_20_), 141.9 (C_2_), 141.2 (C_3_), 134.9 (C_9_), 131.9 (C_18_), 130.4 (C_10_), 130.0 (C_17_), 125.0 (C_1/4_), 121.1 (C_6/7_), 119.2 (C_19_), 110.1 (C_16_), 41.5 (C_8_), 39.2 (C_5_), 32.0 (C_11_), 31.8 (C_13_), 29.8 (C_12_), 22.4 (C_14_), 14.0 (C_15_). **HR ESI MS**: m/z = 302.1903 [M+H]^+^, calculated for C_22_H_24_N^+^: 302.1909.

**
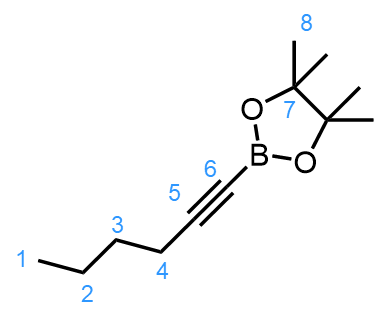
**

**2-(Hex-1-yn-1-yl)-4,4,5,5-tetramethyl-1,3,2-dioxaborolane (3b):** 1-Hexyne (2.8 mL, 24 mmol) in anhydrous diethyl ether (60 mL) was cooled to −78 °C under a N_2_ atmosphere. n-Butyllithium (9.7 mL, 2.5 M in hexanes, 24 mmol) was added dropwise, followed by stirring at −78 °C for 1 h. 4,4,5,5-Tetramethyl-2-(1-methylethoxy)-1,3,2-dioxaborolane (4.1 mL, 20 mmol) in anhydrous diethyl ether (60 mL) was then added. The reaction mixture was stirred at rt for 4 h before being cooled to ‒78 °C, followed by quenching with HCl (7.5 mL, 4.0 M in dioxane, 30 mmol) and stirring at rt for an additional 1 h. The solvent was removed under reduced pressure and the crude product was purified via column chromatography (Teledyne Isco Combi Flash Rf+ system, 80 g Al_2_O_3_, hexanes‒EtOAc, 0–100% elution) to give the title compound as a yellow oil. (1.30 g, 6.25 mmol, 31%) **^1^H NMR** (400 MHz, CDCl_3_, 298 K): δ 2.25 (t, *J* = 7.1 Hz, 2H, H_4_), 1.56 – 1.46 (m, 2H, H_3_), 1.46 – 1.36 (m, 2H, H_2_), 1.26 (s, 12H, H_8_), 0.88 (t, *J* = 7.3 Hz, 3H, H_1_). Characterisation data are consistent with those reported previously.^[69]^

**
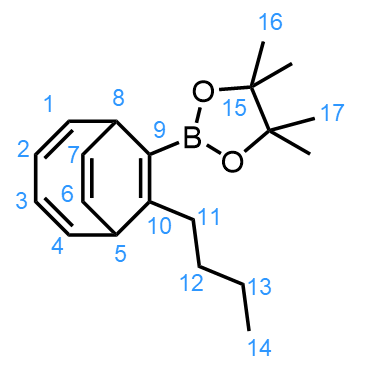
**

**(±)-4,4,5,5-Tetramethyl-2-((2*Z*,4*Z*)-8-butylbicyclo[4.2.2] deca-2,4,7,9-tetraen-7-yl)-1,3,2-dioxaborolane (4b)**: An oven-dried Schlenk tube containing anhydrous ZnI_2_ (128 mg, 400 μmol) was heated at 280 °C under high vacuum for 5 min. After cooling to rt and flushing with N_2_, CoBr_2_(dppe) (124 mg, 200 μmol), Zn (40 mg, 600 μmol), and anhydrous 1,2-dichloroethane (2 mL) were added and the reaction mixture was then deaerated by three freeze-pump-thaw cycles. Cyclooctatetraene (208 mg, 2.00 mmol) was added into the reaction mixture. **3b** (624 mg, 3.00 mmol in 2 mL of anhydrous 1,2-dichloroethane, deaerated) was slowly added using a syringe pump over 8 h. The reaction was stirred at rt in a H_2_O bath for 16 h. After completion, the suspension was filtered through a short pad of SiO_2_ (5 g) gel eluting with Et_2_O. The solvent was removed under reduced pressure, and the crude product was purified by column chromatography (Teledyne Isco CombiFlash Rf+ system, 24 g SiO_2_, n‑hexane‒EtOAc, 0–100% elution) to give the title compound as a colourless oil. (178 mg, 570 μmol , 29%) **^1^H NMR** (400 MHz, CDCl_3_, 298 K): δ 6.31 – 6.24 (m, 1H, H_2_), 6.13 – 6.05 (m, 1H, H_3_), 5.76 – 5.48 (m, 4H, H_1/4/6/7_), 3.49 – 3.37 (m, 1H, H_8_), 3.35 – 3.24 (m, 1H, H_5_), 2.54 – 2.38 (m, 2H, H_11_), 1.43 – 1.28 (m, 4H, H_12/13_), 1.24 (m, 12H, H_16/17_), 0.88 (t, *J* = 7.2 Hz, 3H, H_14_). **^13^C NMR** (151 MHz, CDCl_3_, 298 K): δ 151.1 (C_10_), 143.2 (C_2_), 140.5 (C_3_), 124.9 (C_7_), 123.4 (C_6_), 122.3 (C_1_), 121.4 (C_4_), 82.7 (C_15_), 41.0 (C_5_), 37.0 (C_8_), 34.1 (C_12_), 33.2 (C_11_), 25.1 (C_16_), 24.6 (C_17_), 22.5 (C_13_), 14.0 (C_14_). **HR ASAP MS**: m/z =313.2338 [M+H]^+^, calculated for C_20_H_30_^11^BO_2_^+^: 313.2333.

**
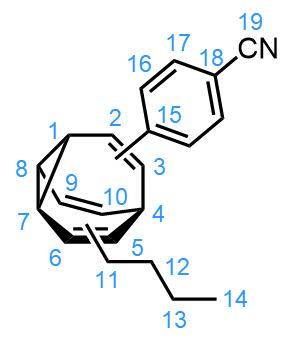
4‑Cyanophenyl-butyl-BV (1b)**: **4b** (535 mg, 1.70 mmol) and thioxanthone (7.2 mg, 34 μmol) were dissolved in anhydrous THF (5 mL). The reaction mixture was irradiated with a 365 nm LED lamp while being kept at 25 °C by immersion in a H_2_O bath for 24 h. The solvent was removed under reduced pressure and the crude product was purified by column chromatography (Teledyne Isco CombiFlash Rf+ system, 80 g Al_2_O_3_, hexanes‒CH_2_Cl_2_, 0–100% elution) to give compound **5b** (250 mg, crude). The broad peaks in the ^1^H-NMR spectrum indicated the successful formation of BV. The product was carried into the next step without further purification: **5b** (250 mg, crude), 4‑bromobenzonitrile (146 mg, 800 μmol) and Pd(PPh_3_)_4_ (44 mg, 40 μmol) were dissolved in a mixture of THF (4 mL) and 10% aqueous NaOH solution (1.2 mL). The mixture was deaerated by three freeze-pump-thaw cycles and stirred at 60 °C for 16 h. The reaction mixture was diluted with diethyl ether and dried over MgSO_4_. The solvent was removed under reduced pressure and the crude product was purified by column chromatography (Teledyne Isco Combi Flash Rf+ system, 12 g SiO_2_, hexanes–CH_2_Cl_2_, 0–100% elution) to give the title compound as a yellow oil (58 mg, 0.20 mmol, 12%). **^1^H NMR** (500 MHz, CDCl_3_, 210 K) δ 7.68 – 7.61 (m, 2H, H_17_), 7.49 (m, 0.39H, H_16_, γ,β'+γ,γ'), 7.43 (m, 1.60H, H_16_, β,β'+β,γ'), 6.28 – 6.22 (m, 0.81H, H_2_, β,β'+β,γ'), 6.18 – 6.11 (m, 0.19H, H_3_, γ,β'+γ,γ'), 6.09 – 5.84 (m, 2H, H_9/10_), 5.79 (d, *J* = 7.7 Hz, 0.54H, H_6_, β,β'), 5.75 (d, *J* = 7.9 Hz, 0.15H, H_6_, γ,β'), 5.68 (d, *J* = 9.1 Hz, 0.26H, H_5_, β,γ’), 5.60 (d, *J* = 8.9 Hz, 0.05H, H_5_, γ,γ’), 3.01 (m, 0.26H, H_4_, β,γ’), 2.88 (d, *J* = 8.7 Hz, 0.46H, H_4_, β,β'), 2.74 - 2.60 (m, 0.28H, H_4_, γ,β’+γ,γ’), 2.55 – 2.34 (m, 3H, H_1/7/8_), 2.17 – 2.03 (m, 2H, H_11_), 1.52 – 1.26 (m, 2H, H_12_), 1.20 (m, 2H, H_13_), 0.93 – 0.84 (m, 1.67H, H_14_, β,β'), 0.76 (m, 1.33H, H_14_, β,γ'+γ,β'+γ,γ'). **^13^C NMR** (126 MHz, CDCl_3_, 210 K) δ 149.5 (C_19_, γ,β'), 147.8 (C_19_, β,β'+β,γ'+γ,γ'), 142.3 (C_5_, γ,β'), 141.4 (C_5_, β,β'), 140.2 (C_6_, β,γ'), 139.6 (C_6_, γ,γ'), 139.1 (C_3_, β,γ'), 138.7 (C_3_, β,β'), 137.3 (C_3_, γ,β'), 136.9 (C_3_, γ,γ'), 132.6 (C_17_), 128.1 (C_9_), 128.0 (C_2_, β,β'), 127.6 (C_2_, β,γ'), 127.2 (C_16_, γ,β'+γ,γ'/C_10_), 126.8 (C_3_, γ,β'+γ,γ'), 126.6 (C_16_, β,β'+β,γ'), 120.5 (C_6_, β,β'), 120.0 (C_18_), 119.7 (C_6_, γ,β'), 119.1 (C_5_, γ,γ'), 118.7 (C_5_, β,γ'), 109.6 (C_15_, γ,β'), 109.5 (C_15_, γ,γ'),109.3 (C_15_, β,β'), 109.1 (C_15_, β,γ'), 41.6 (C_11_, γ,γ'), 41.0 (C_11_, β,γ'), 40.2 (C_11_, γ,β'), 40.0 (C_11_, β,β'), 39.0 (C_4_, β,β'), 34.7 (C_4_, γ,β'), 34.3 (C_4_, β,γ'), 31. 9 (C_12_, γ,γ'), 31.5 (C_12_, γ,β'), 30.5 (C_12_, β,β'), 30.5 (C_12_, β,γ'), 30.0 (C_4_, γ,γ'), 24.3 (C_1_ or C_7_ or C_8_, β,γ'), 23.2 (C_12_, γ,γ'), 22.9 (C_1_ or C_7_ or C_8_, γ,γ'), 22.7 (C_13_, γ,β'), 22.6 (C_13_, β,γ'), 22.5 (C_1_ or C_7_ or C_8_, γ,γ'), 22.3 (C_1_ or C_7_ or C_8_, γ,β'), 22.3 (C_13_, β,β'), 21.5 (C_1_ or C_7_ or C_8_, β,β'), 21.4 (C_1_ or C_7_ or C_8_, β,γ'), 21.3 (C_1_ or C_7_ or C_8_, β,β'), 21.2 (C_1_ or C_7_ or C_8_, β,β'), 21.0 (C_1_ or C_7_ or C_8_, β,γ'), 20.1 (C_1_ or C_7_ or C_8_, γ,β'), 20.1 (C_1_ or C_7_ or C_8_, γ,γ'), 19.7 (C_1_ or C_7_ or C_8_, γ,β'), 14.8 (C_14_, γ,γ'), 14.7 (C_14_, β,γ'), 14.6 (C_14_, γ,β'), 14.5 (C_14_, β,β'). **HR ESI MS**: m/z = 310.1577 [M+Na]^+^, calculated for C_21_H_21_NNa^+^: 310.1566.

**
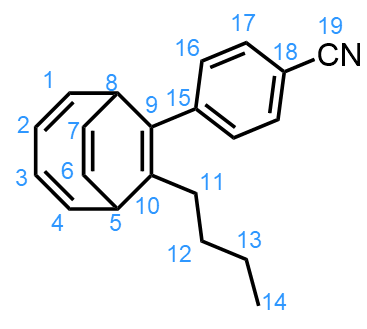
**

**(±)-4-((2*Z*,4*Z*)-8-Butylbicyclo[4.2.2]deca-2,4,7,9-tetraen-7-yl)benzonitrile (2b)**: **4b** (183 mg, 590 μmol), 4-bromobenzonitrile (108 mg, 590 μmol), and Pd(PPh_3_)_4_ (33 mg, 30 μmol) was dissolved in a mixture of THF (3 mL) and 10% aqueous NaOH solution (0.9 mL). The mixture was deaerated by three freeze-pump-thaw cycles and stirred at 60 °C for 16 h. The reaction mixture was diluted with diethyl ether and dried over MgSO_4_. The solvent was removed under reduced pressure and the crude product was purified *via* column chromatography (Teledyne Isco Combi Flash Rf+ system, 12 g SiO_2_, n-hexane–CH_2_Cl_2_ elution) to give the title compound as a yellow oil (56 mg, 0.20 mmol, 34%). **^1^H NMR** (400 MHz, CDCl_3_, 298 K): δ 7.60 – 7.54 (m, 2H, H_17_), 7.27 – 7.21 (m, 1H, H_16_), 6.33 – 6.14 (m, 2H, H_2/3_), 5.82 – 5.72 (m, 4H, H_1/4/6/7_), 3.33 (m, 2H, H_5/8_), 2.15 – 2.05 (m, 1H, H_11E_), 1.93 – 1.84 (m, 1H, H_11A_), 1.40 – 1.30 (m, 2H, H_12_), 1.20 – 1.11 (m, 2H, H_13_), 0.78 (t, *J* = 7.3 Hz, 3H, H_14_). **^13^C NMR** (101 MHz, CDCl_3_, 298 K): δ 146.9 (C_19_), 141.9 (C_2_), 141.2 (C_3_), 134.9 (C_9_), 131.9 (C_17_), 130.4 (C_10_), 130.0 (C_16_), 125.0 (C_7_), 124.8 (C_6_), 121.1 (C_1_), 121.1 (C_4_), 119.2 (C_18_), 110.1 (C_15_), 41.5 (C_5_), 39.2 (C_8_), 32.4 (C_12_), 31.8 (C_11_), 22.7 (C_13_), 14.0 (C_14_). **HR ESI MS**: m/z = 310.1573 [M+Na]^+^, calculated for C_21_H_21_NNa^+^: 310.1566.

4. ^1^H and ^13^C NMR Spectroscopic Characterisation of Synthesised Compounds


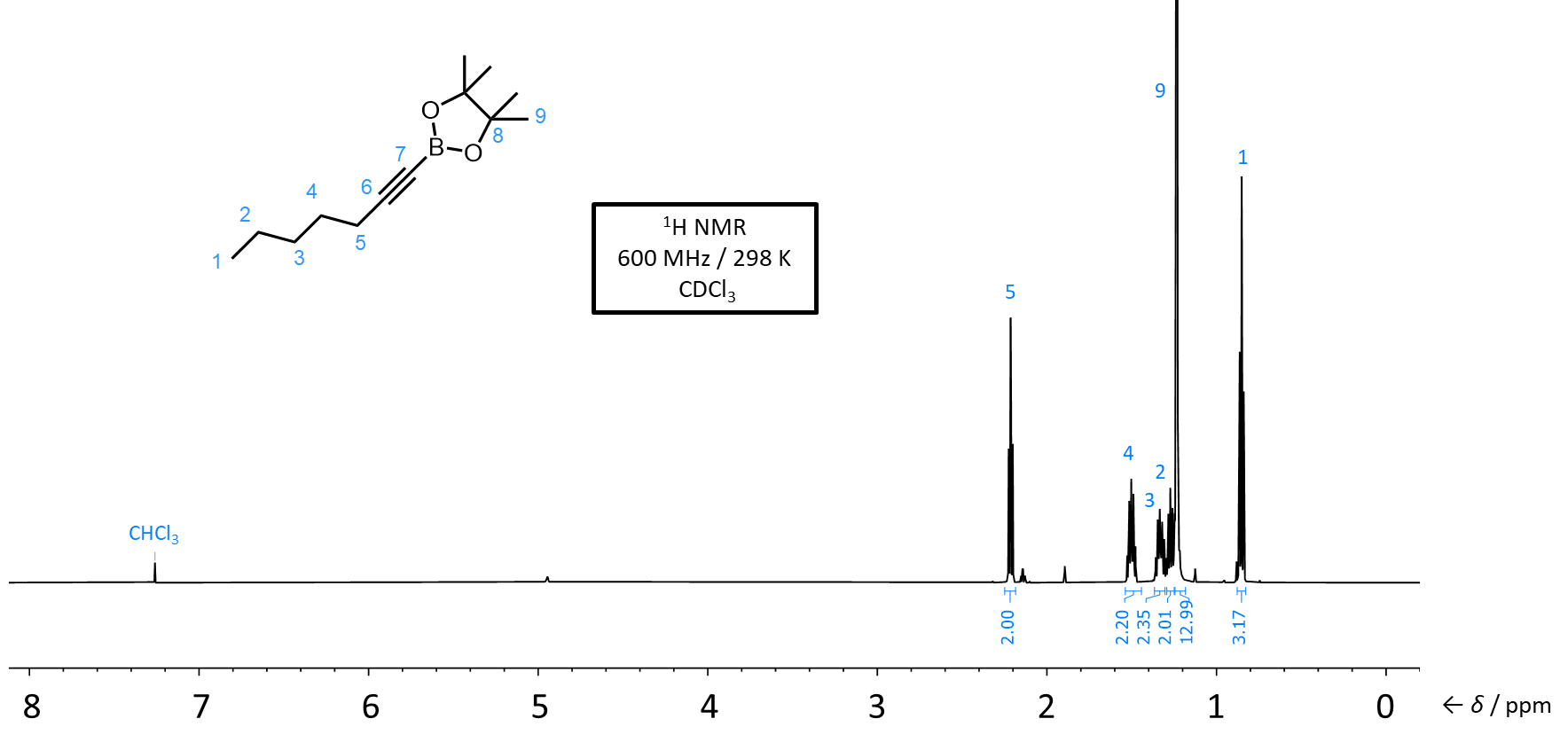


**Figure S2**. ^1^H NMR spectrum of **3a**.


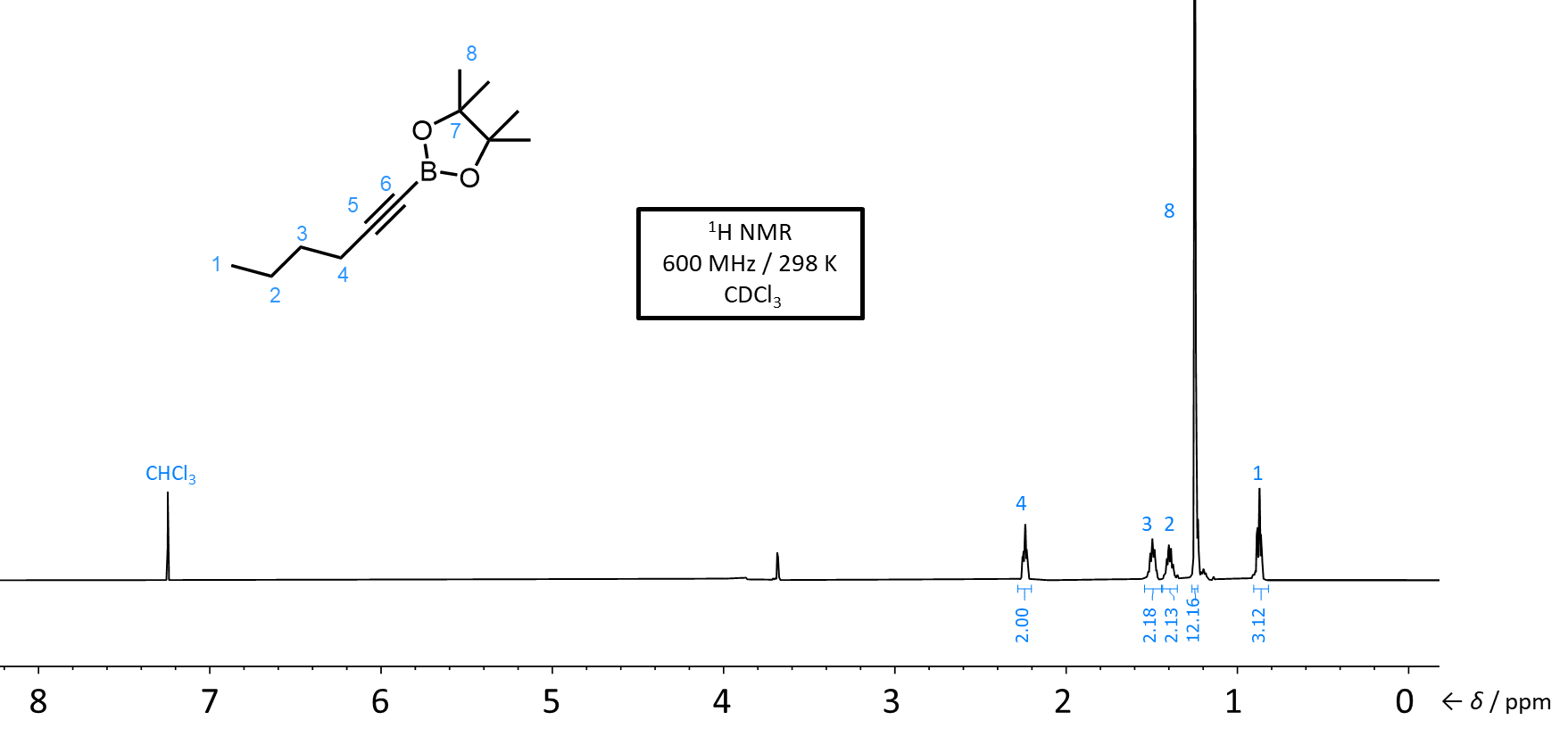


**Figure S3**. ^1^H NMR spectrum of **3b**.


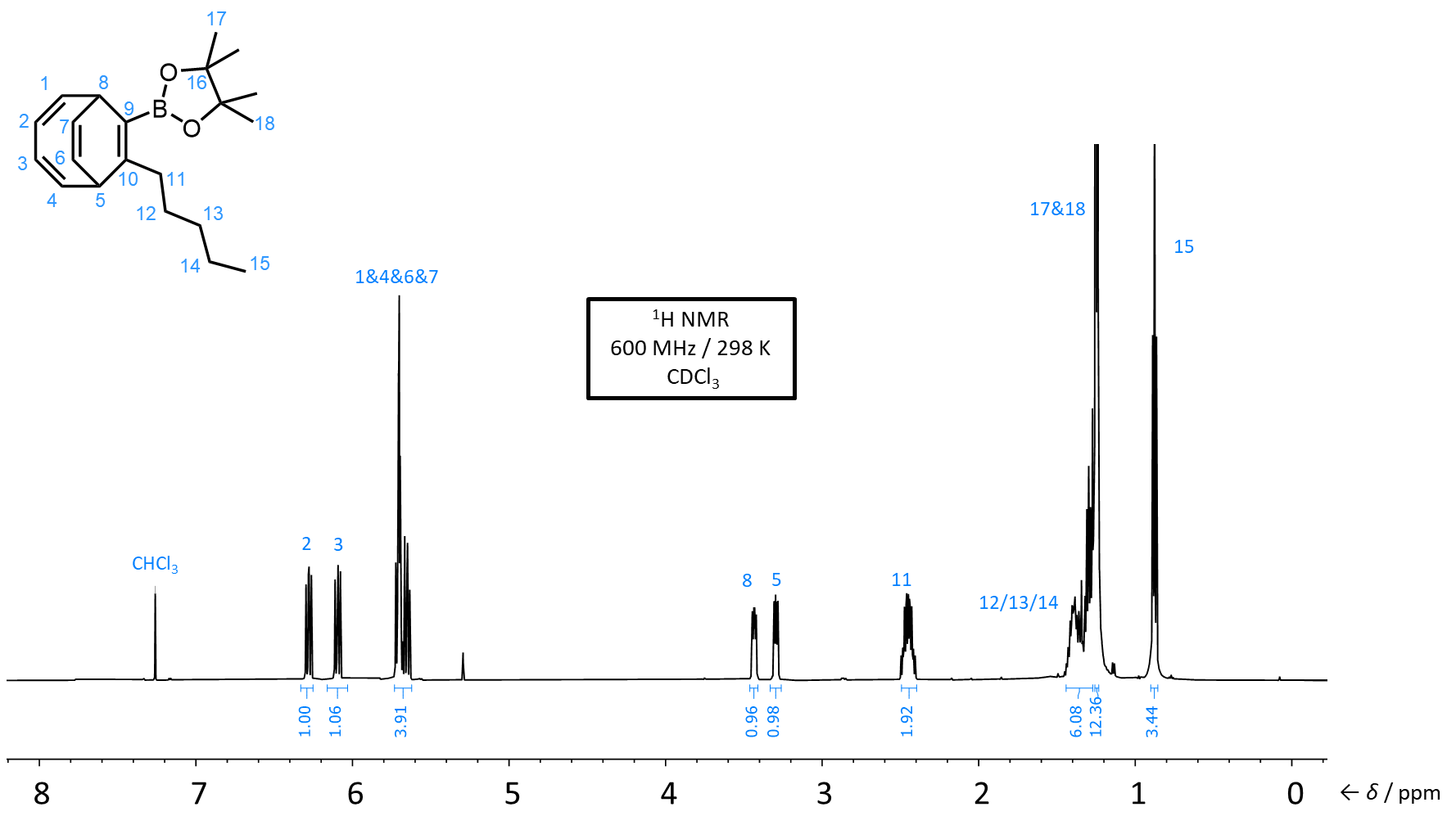


**Figure S4**. ^1^H NMR spectrum of **4a**.

**
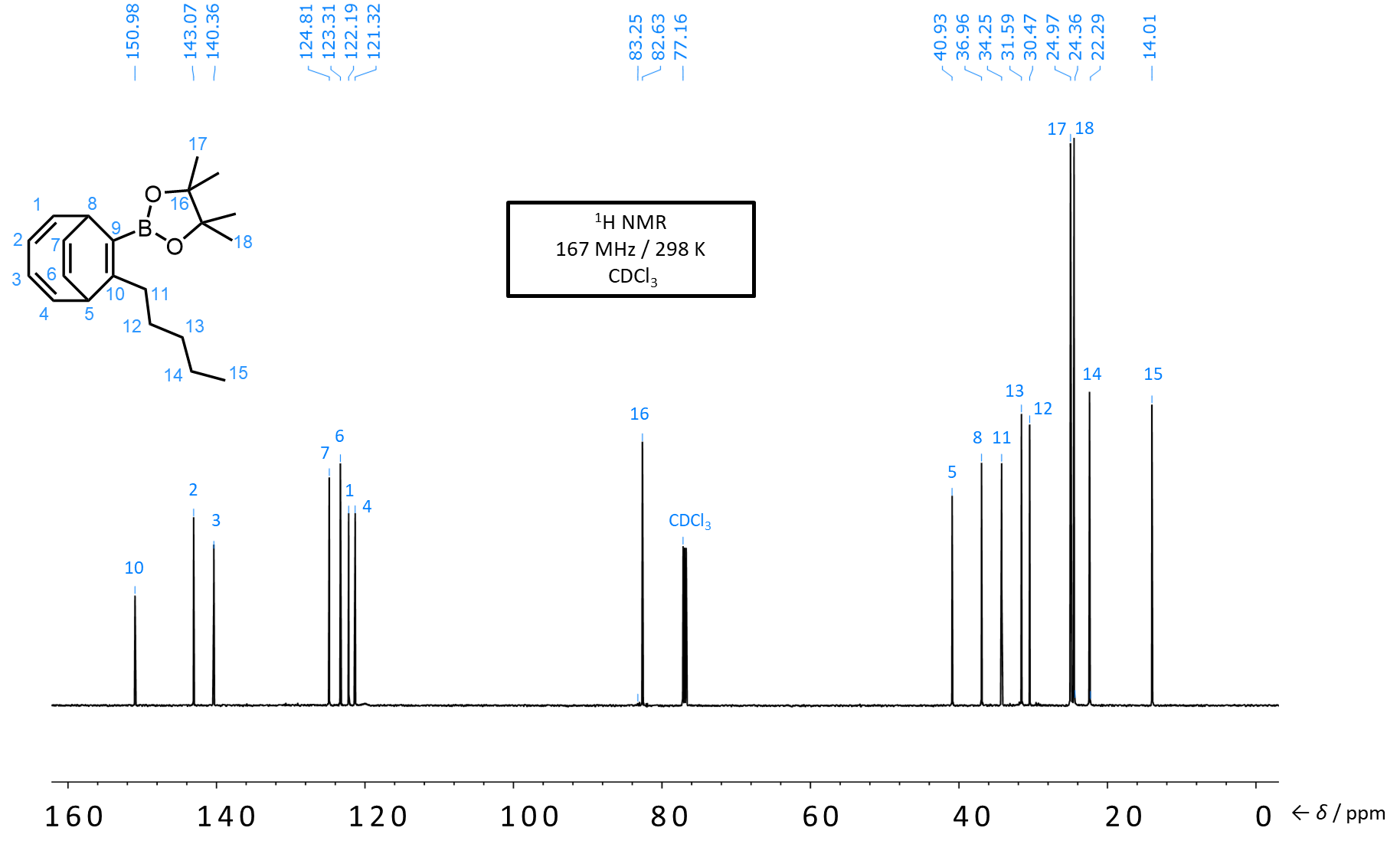
**

**Figure S5**. ^13^C NMR spectrum of **4a**.


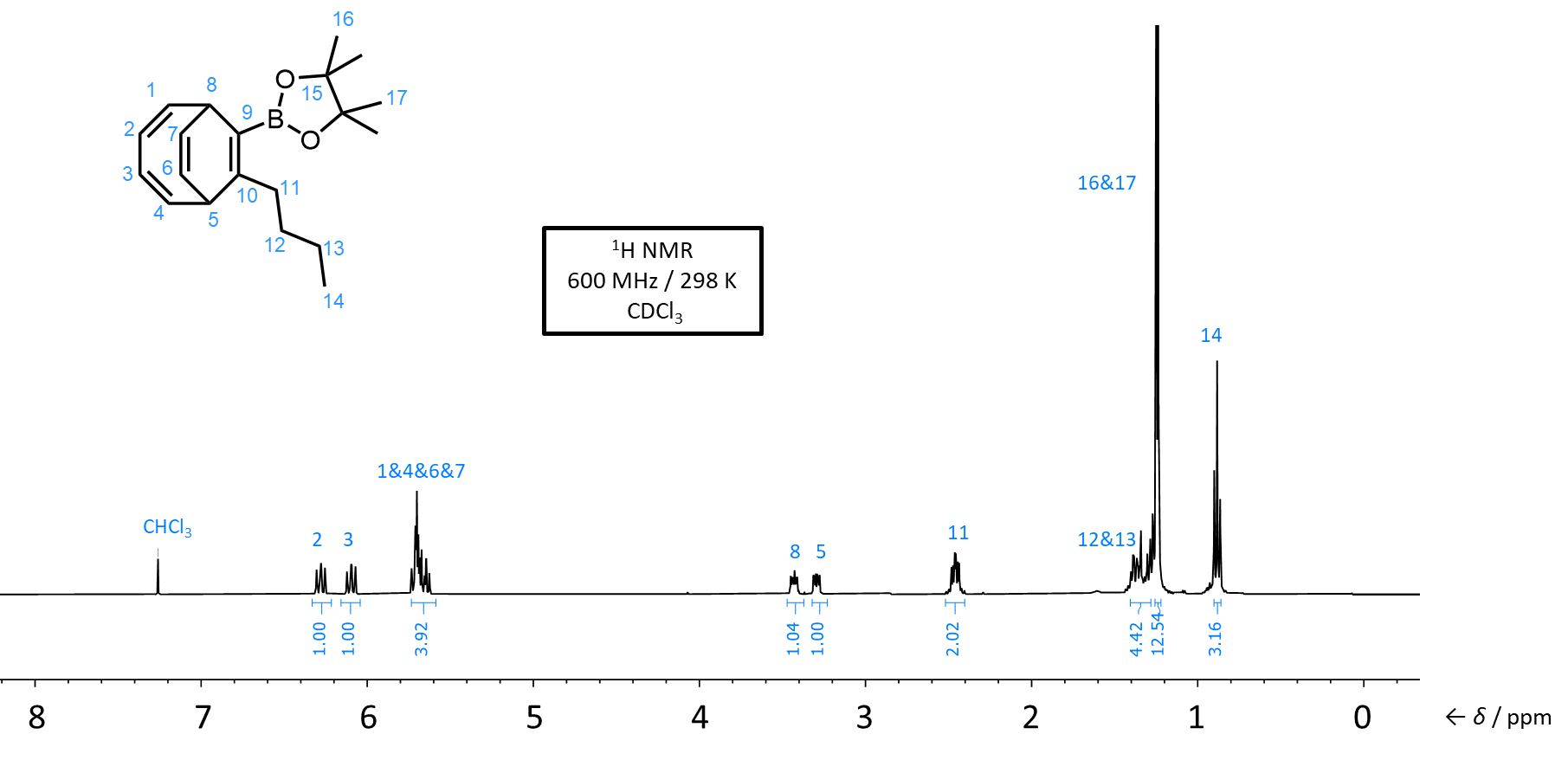


**Figure S6**. ^1^H NMR spectrum of **4b**.


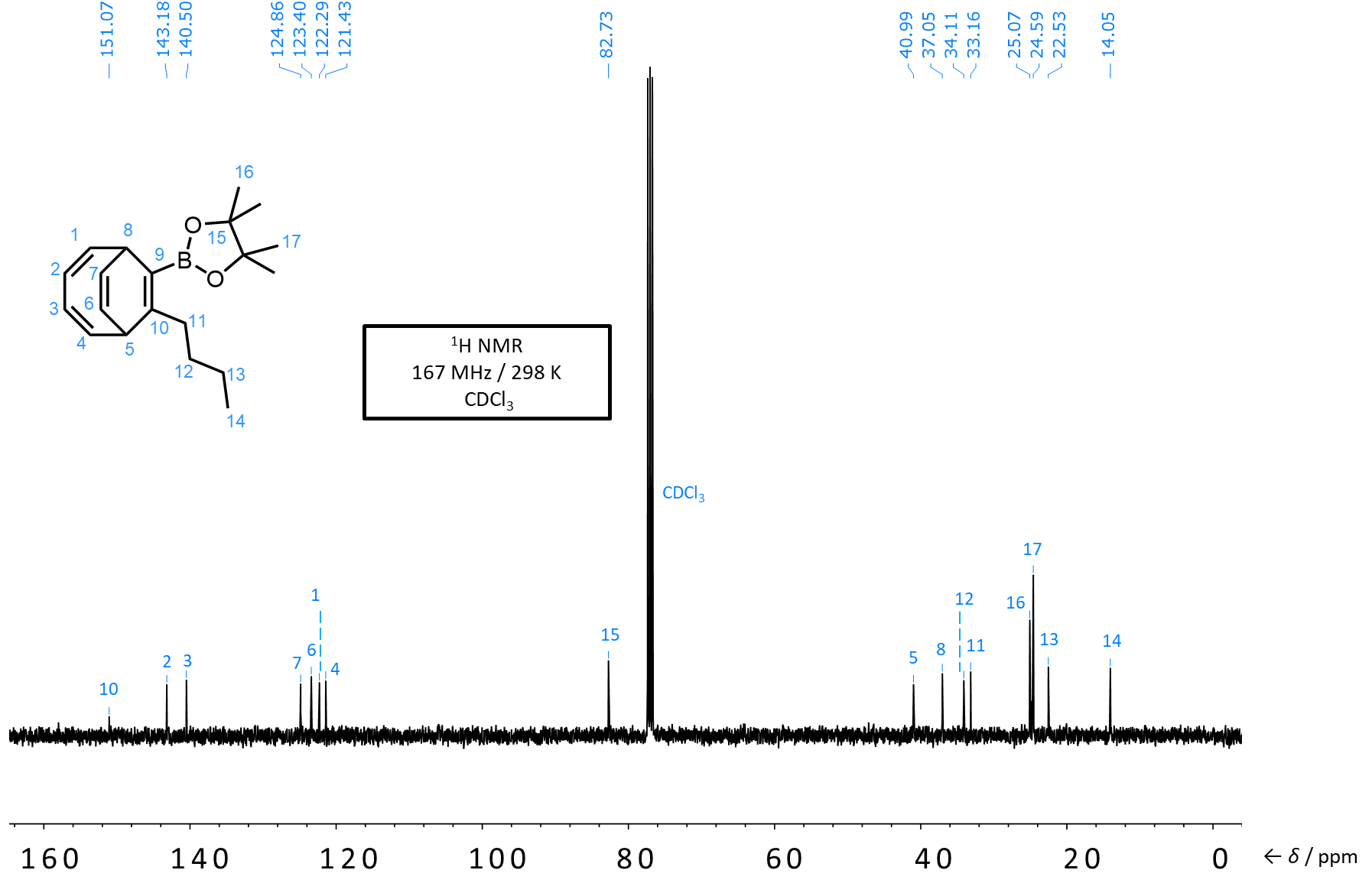


**Figure S7**. ^13^C NMR spectrum of **4b**.


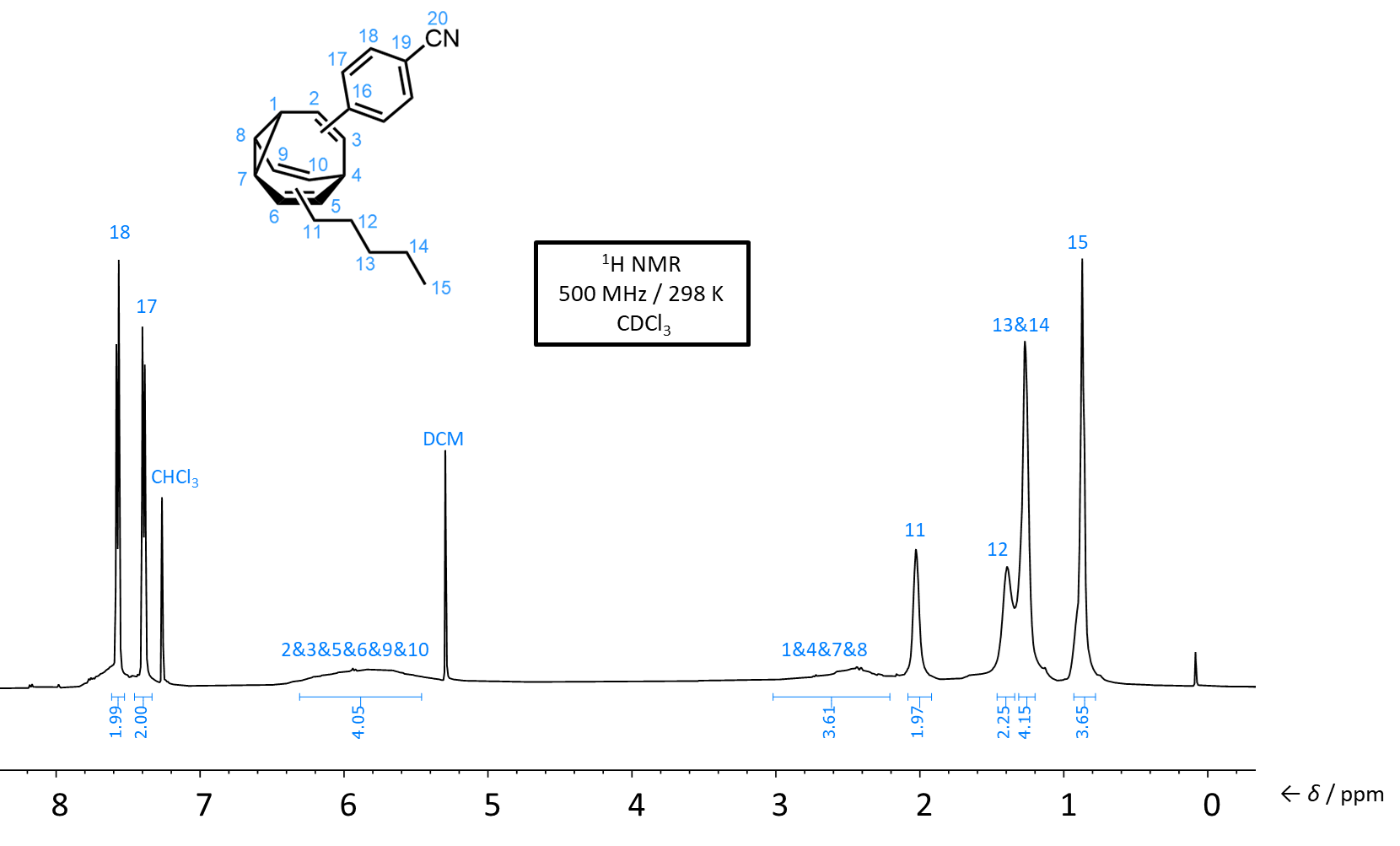


**Figure S8**. ^1^H NMR spectrum (298 K) of **1a**.


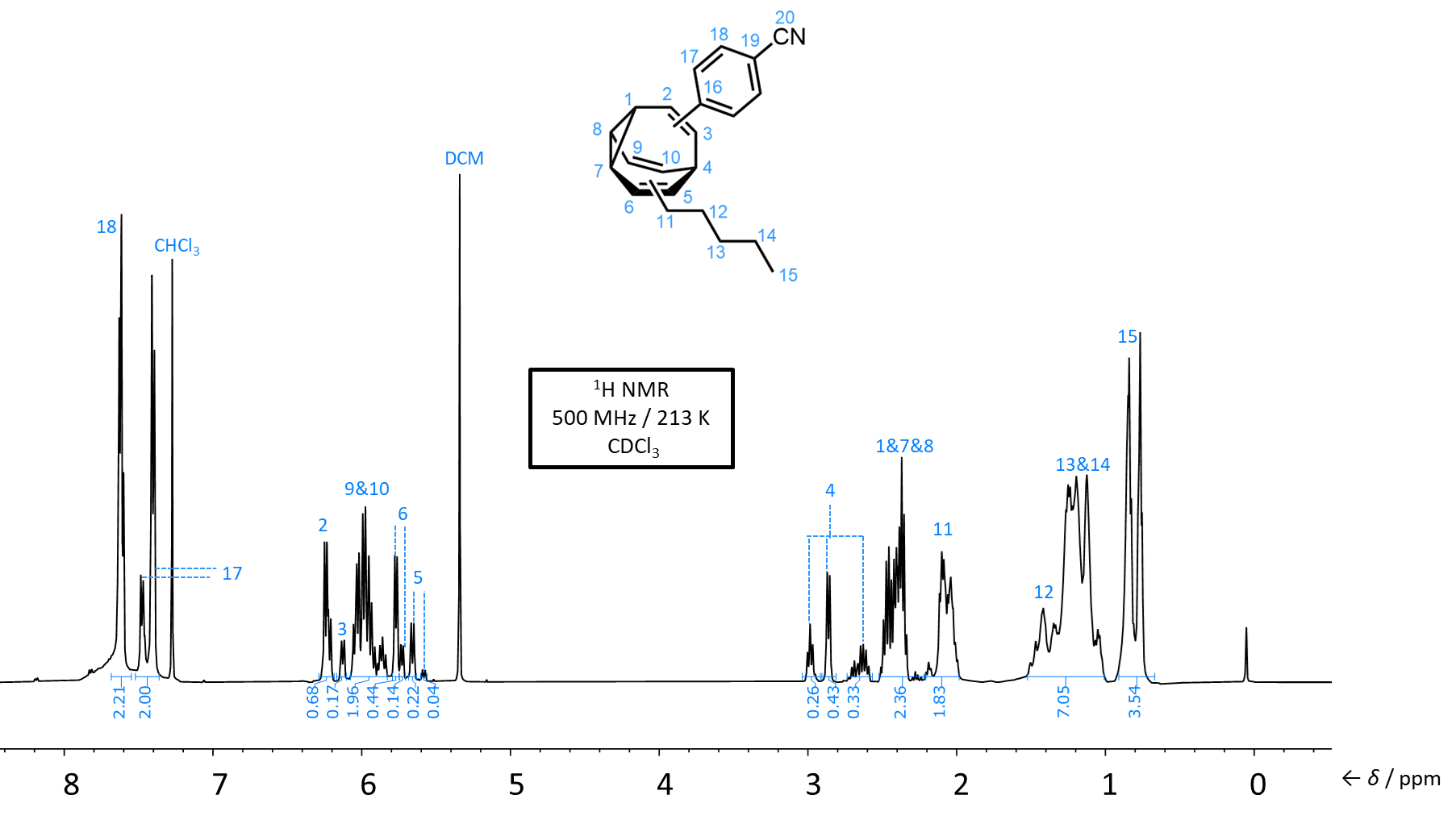


**Figure S9**. ^1^H NMR spectrum (210 K) of **1a**.


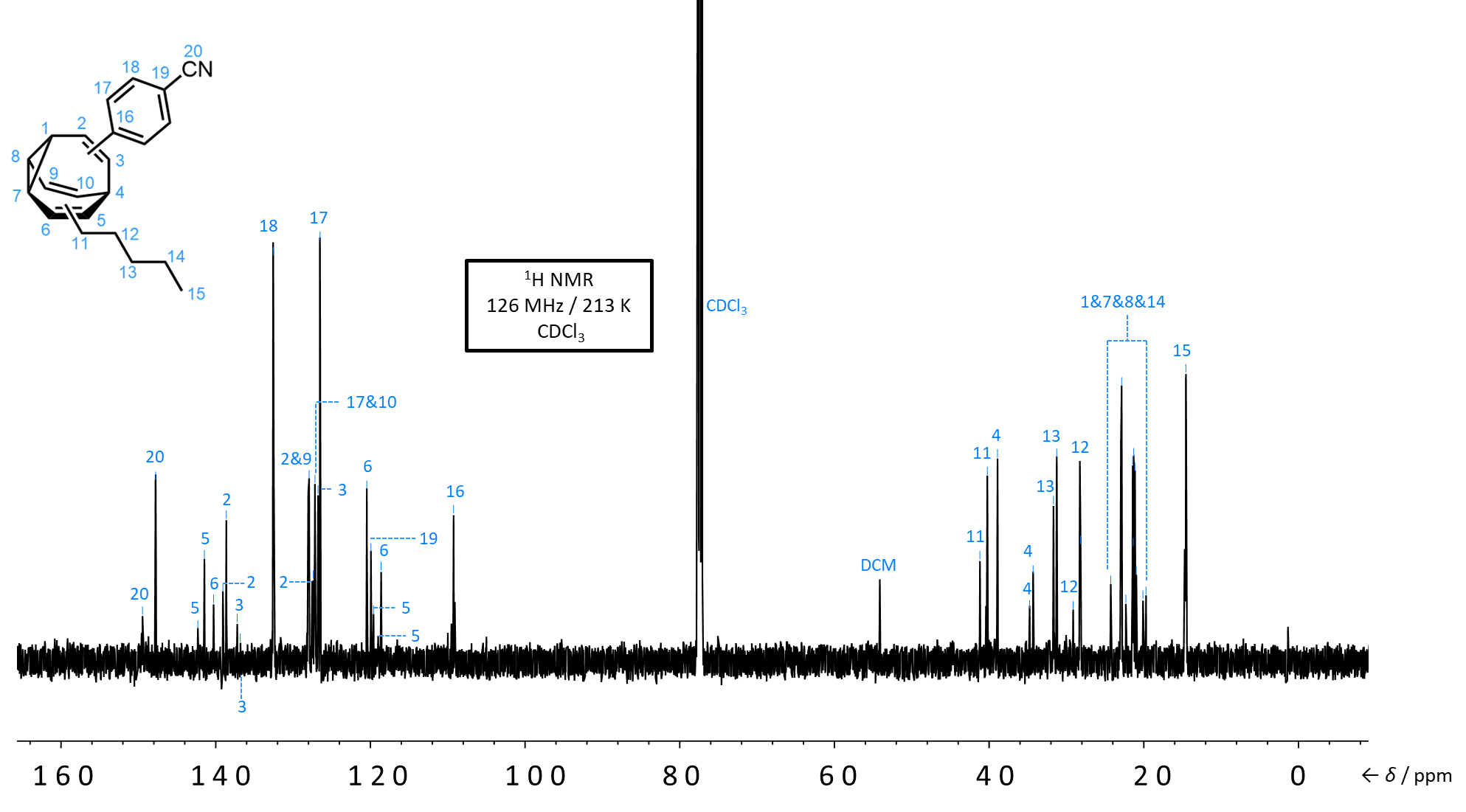


**Figure S10**. ^13^C NMR spectrum (210 K) of **1a**.


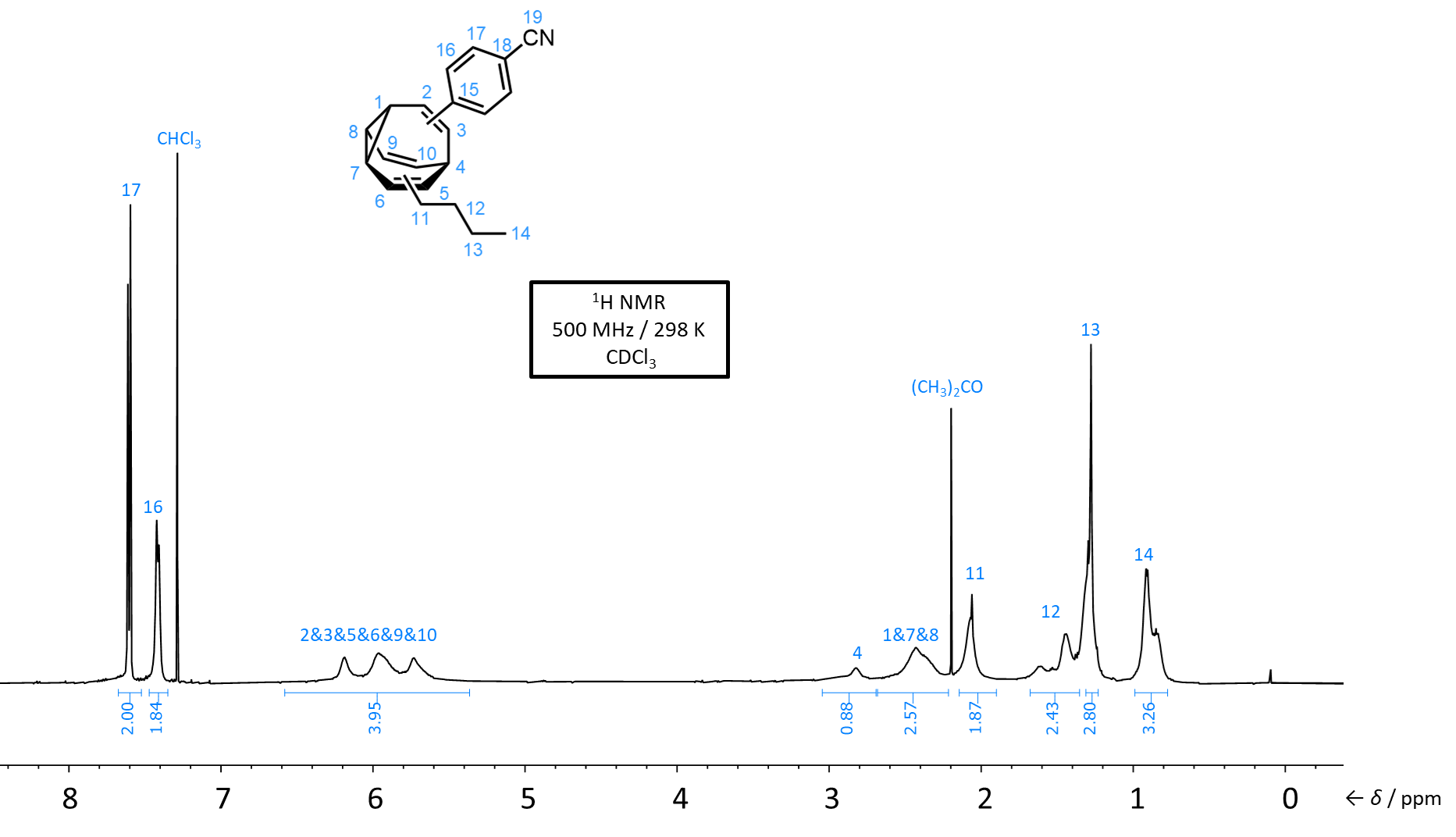


**Figure S11**. ^1^H NMR spectrum (298 K) of **1b**.


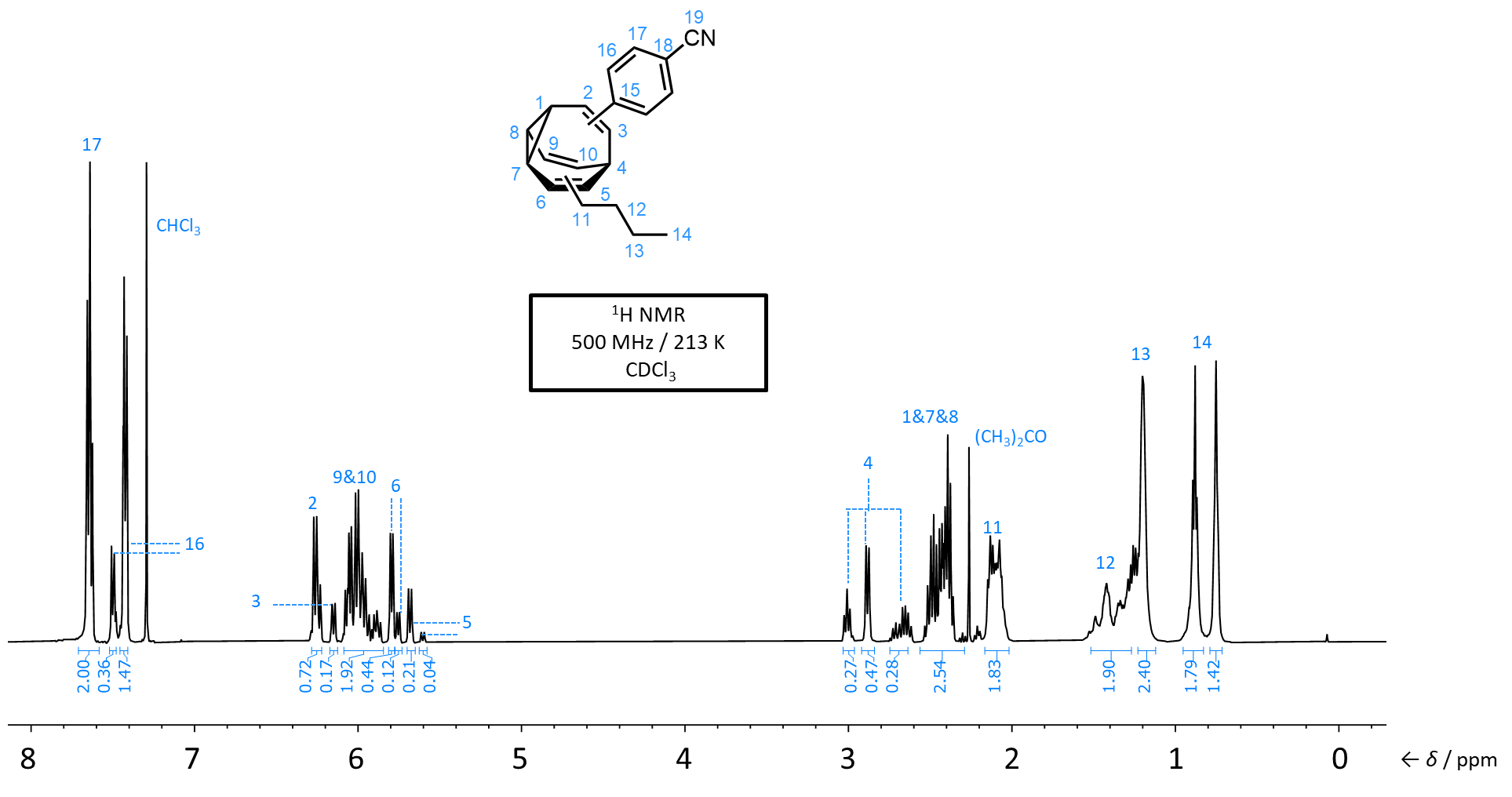


**Figure S12**. ^1^H NMR spectrum (210 K) of **1b**.


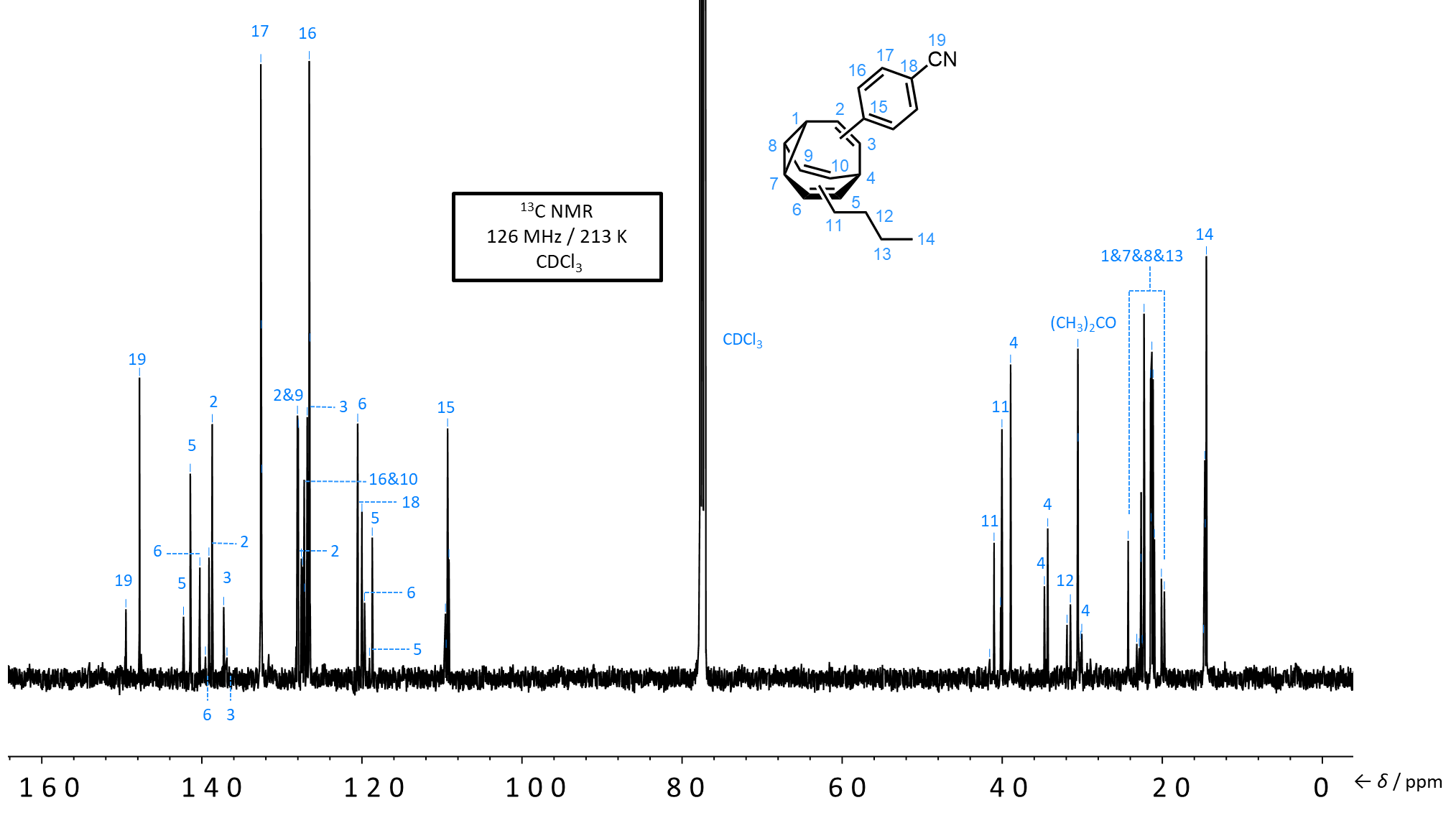


**Figure S13**. ^13^C NMR spectrum (210 K) of **1b**.


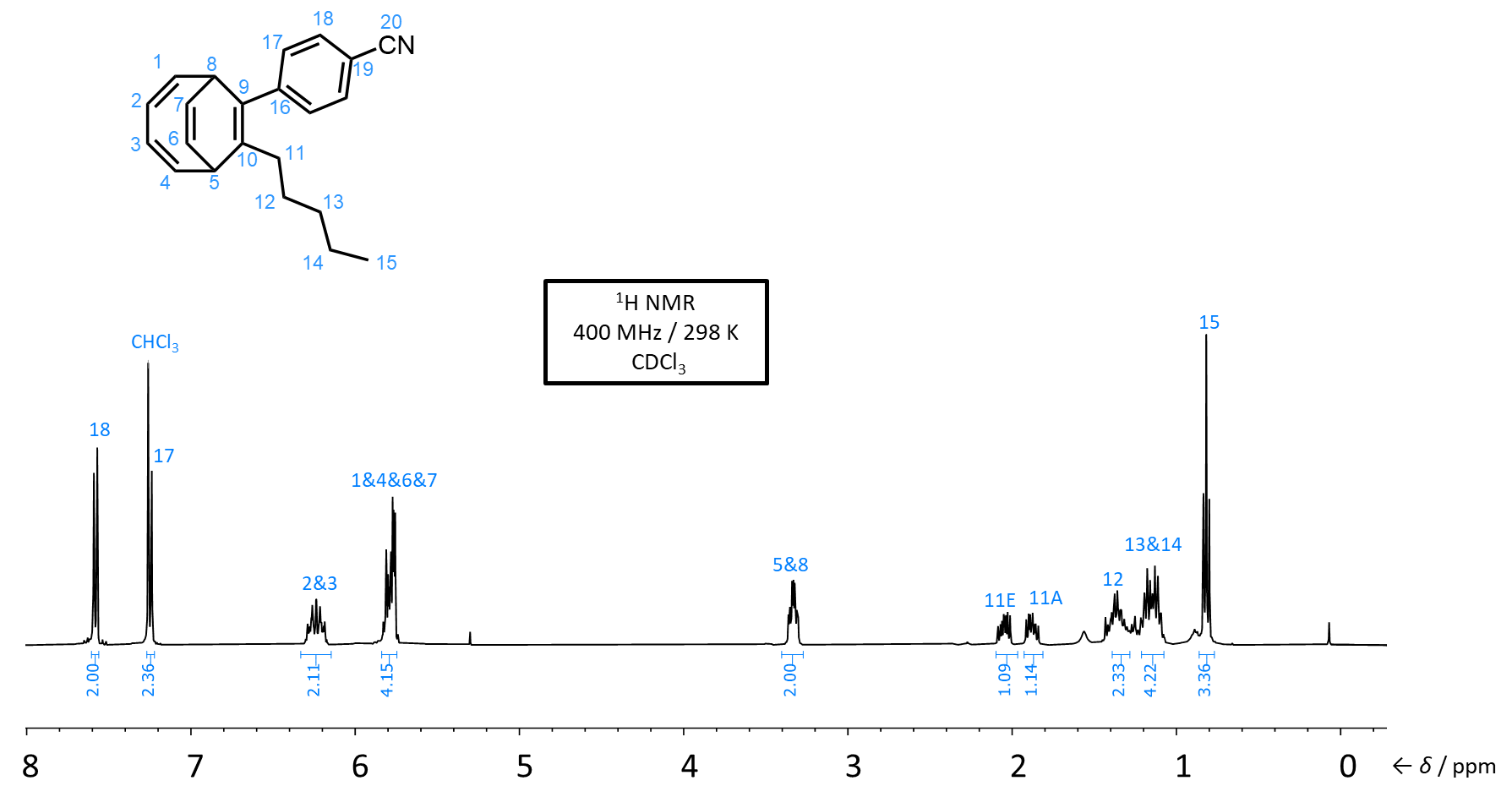


**Figure S14**. ^1^H NMR spectrum of **2a**.


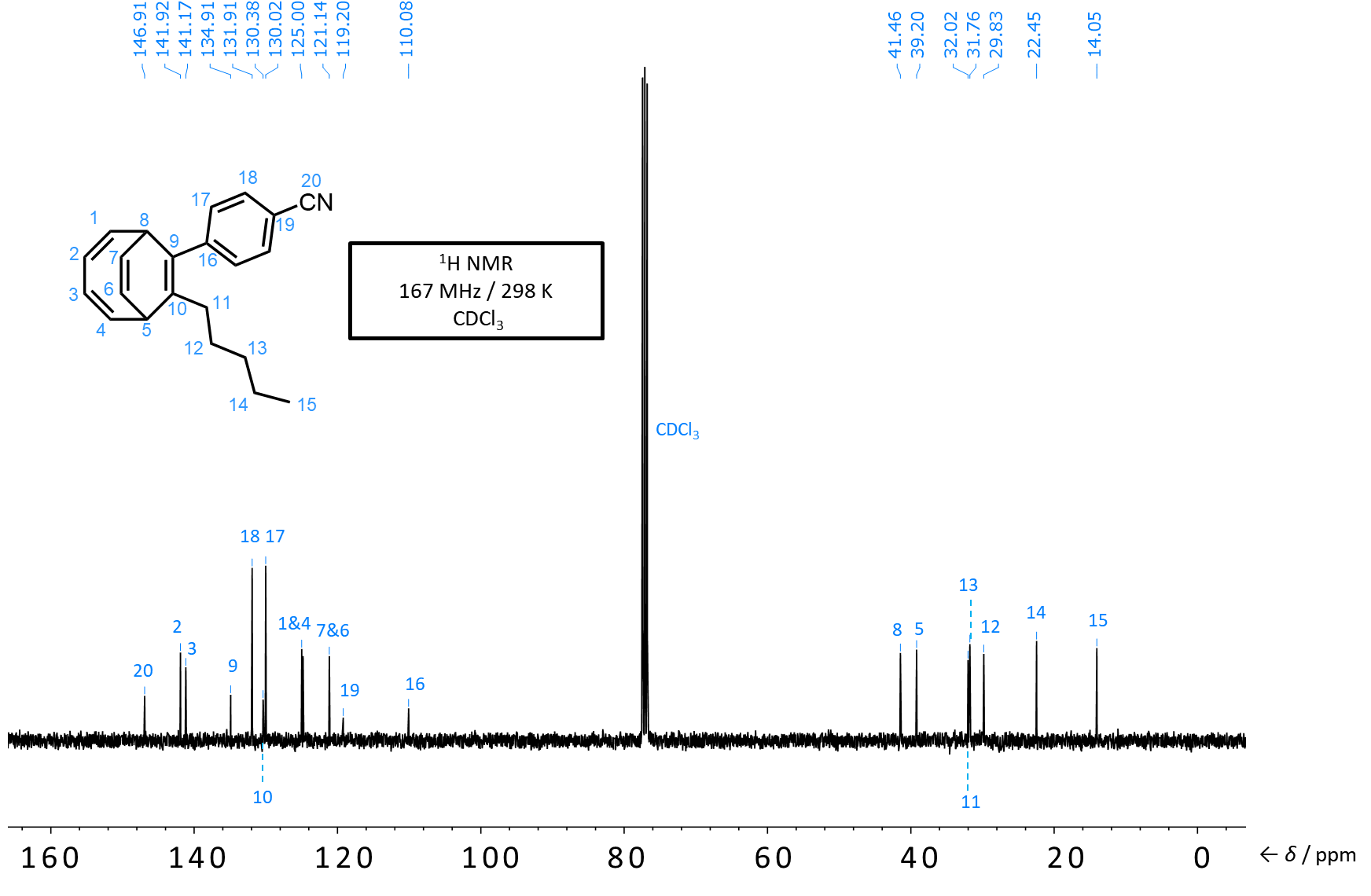


**Figure S15**. ^13^C NMR spectrum of **2a**.


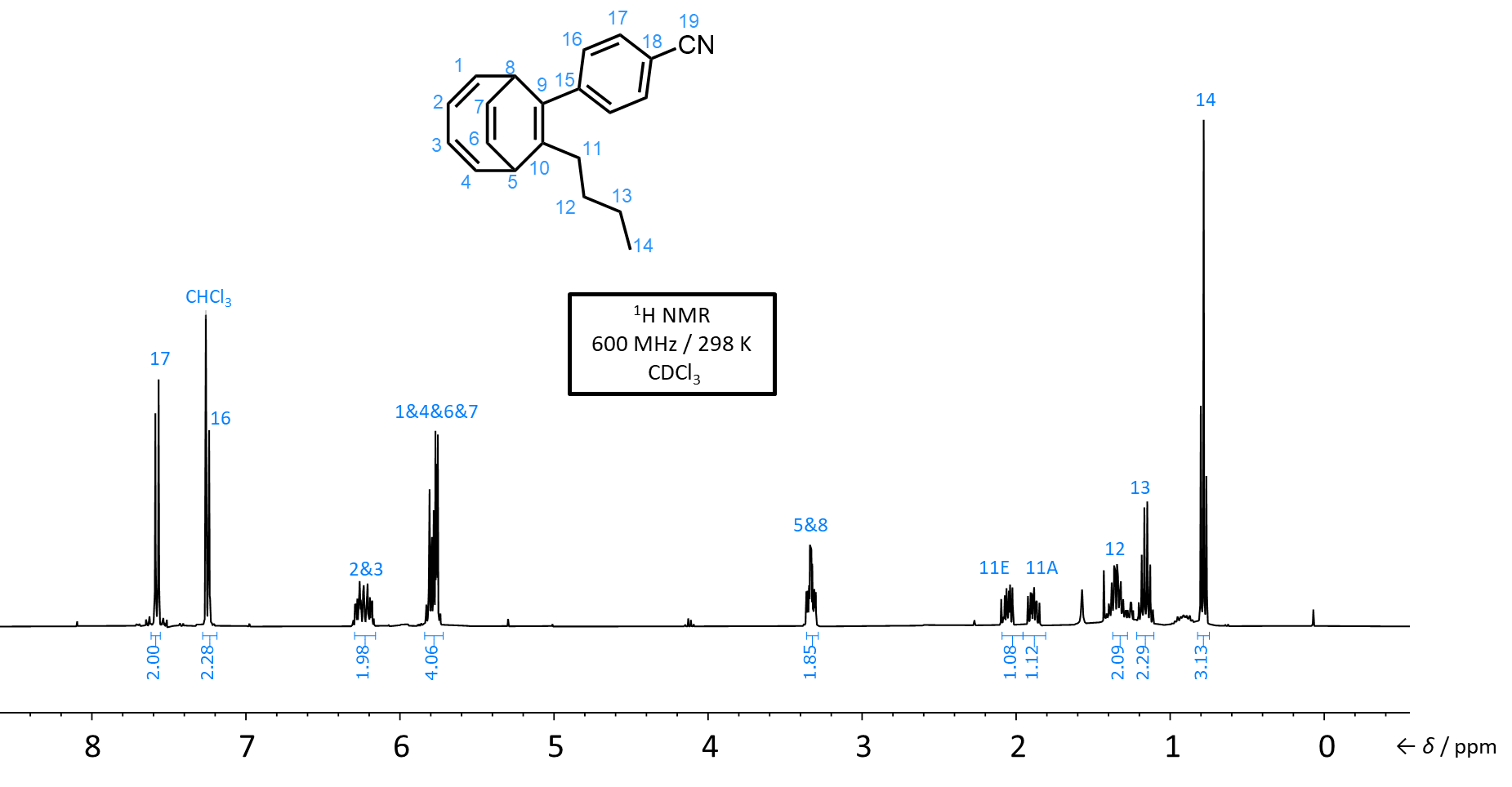


**Figure S16**. ^1^H NMR spectrum of **2b**.


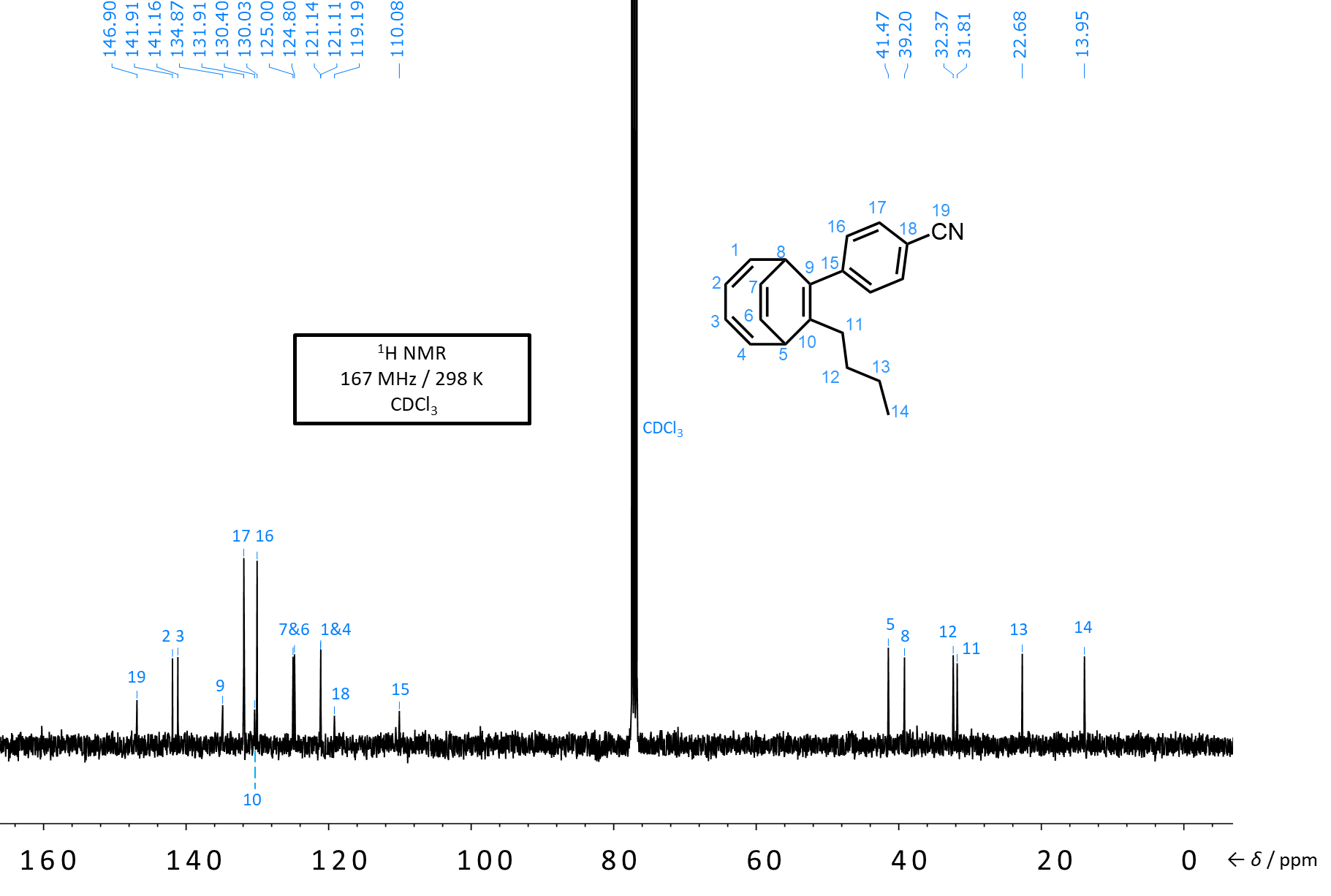


**Figure S17**. ^13^C NMR spectrum of **2b**.

Samples of **1** and **2** were handled under ambient laboratory conditions without precautions to exclude oxygen or moisture. A comparison of ^1^H NMR spectra of **1b** (Figure S18) show no signs of degradation after the neat material was kept at ambient temperature in air for 3 days. After 2 years under ambient conditions, there are a few low-intensity peaks, which indicate a small amount of decomposition over this extended period.


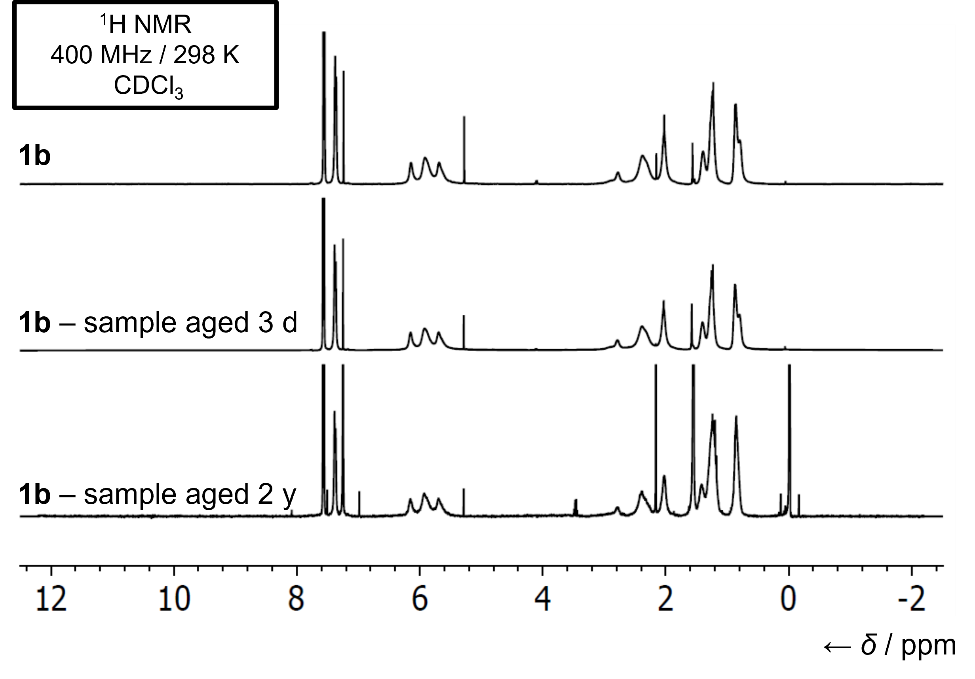


**Figure S18**. ^1^H NMR spectra of **1b** recorded before and after storing the sample under ambient conditions for 3 days and 2 years at 298 K.

5. Structural Assignment of Compound 1, 2 and 4 by 2D NMR

The broad peaks in the room-temperature ^1^H NMR spectra of **1b** resolve into individual peaks at low temperatures, as the rate of Cope rearrangements is slowed compared to the difference in frequency of BV isomers present in solution (Figure S19). This section outlines our attempt to assign these signals at low temperature with the aid of 2D NMR spectroscopic experiments, namely COSY, HMBC and HSQC.


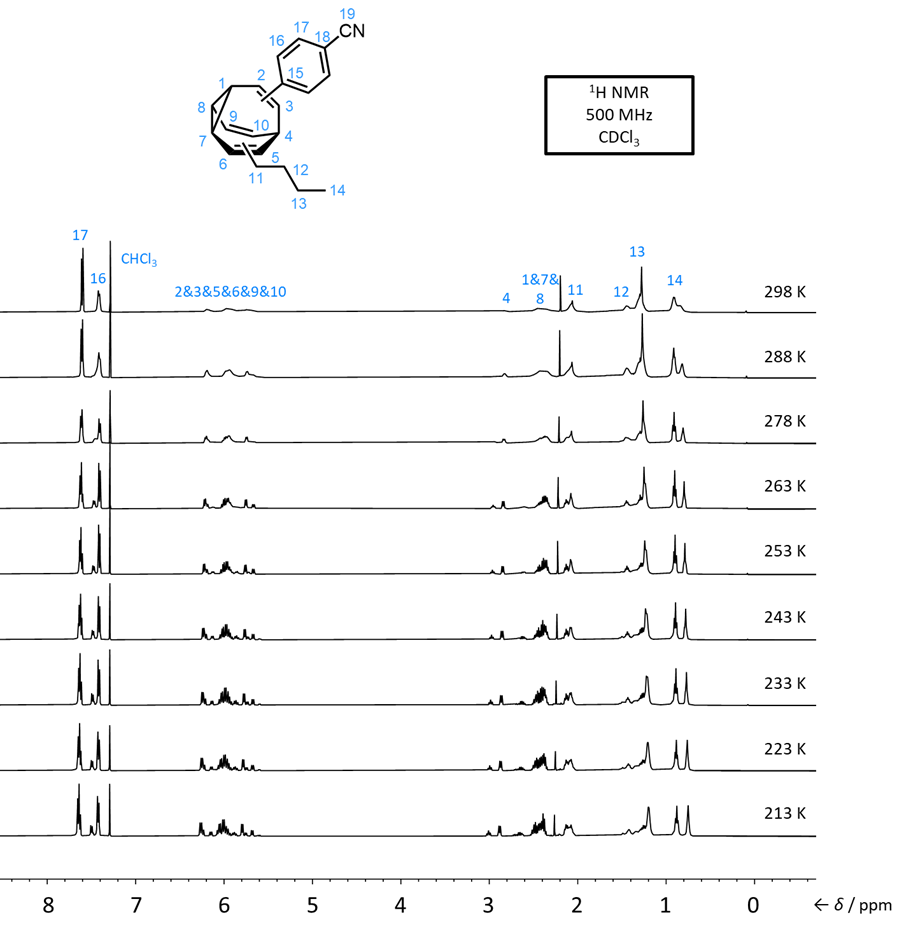


**Figure S19**. Variable-temperature ^1^H-NMR spectra of **1b**.

The assignment of each peak of **1b** and analysis of its isomer distribution was achieved by using 2D NMR spectra obtained at 210 K. Previous studies on bullvalene isomerisation^[70]^ have demonstrated that di-substituted bullvalenes tend to have a distribution in which the presence of each isomer follows: β,β'＞β,γ'＞γ,β'＞γ,γ'. In the ^1^H and ^13^C spectra of **1b**, multiple sets of peaks in the aromatic region are observed to have a ratio close to 55:26:15:4 (Figure S20). It is assumed that the ratio represents the distribution of four isomers (β,β'; β,γ'; γ,β' and γ,γ') in solution and this initial assumption provides a starting point for our assignment.


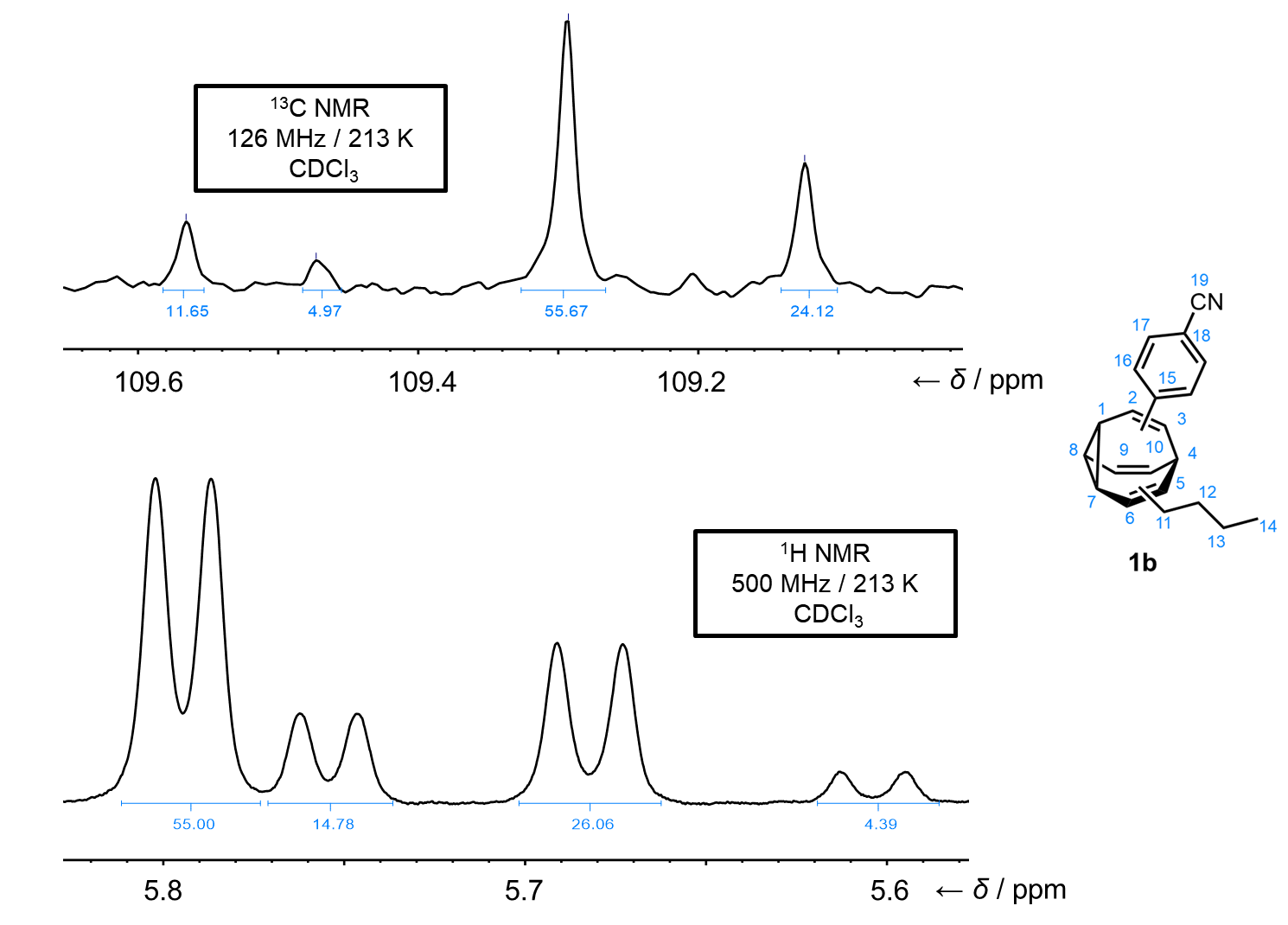


**Figure S20**. Partial ^1^H and ^13^C NMR spectra of **1b** exemplifying a recurring scenario where four similar peaks of intensities roughly 55:26:15:4 are present.

H_16_ and H_17_ were initially assigned due to their characteristic aromatic chemical shifts. The signals arising from each isomer of H_17_ are overlapping, whilst H_16_ shows two separate signals, each constituting a mixture of two isomers.Based on their integrals, the signal at around 7.5 ppm is a mixture of the γ,β' and γ,γ' isomers whilst the signal at around 7.4 ppm is a mixture of the β,β' and β,γ' isomers. Using the HMBC spectrum, C_15_ is determined via correlation with H_16_ (Figure S21) and the ratio of the four observed peaks matches with the initially assumed distribution of four isomers.


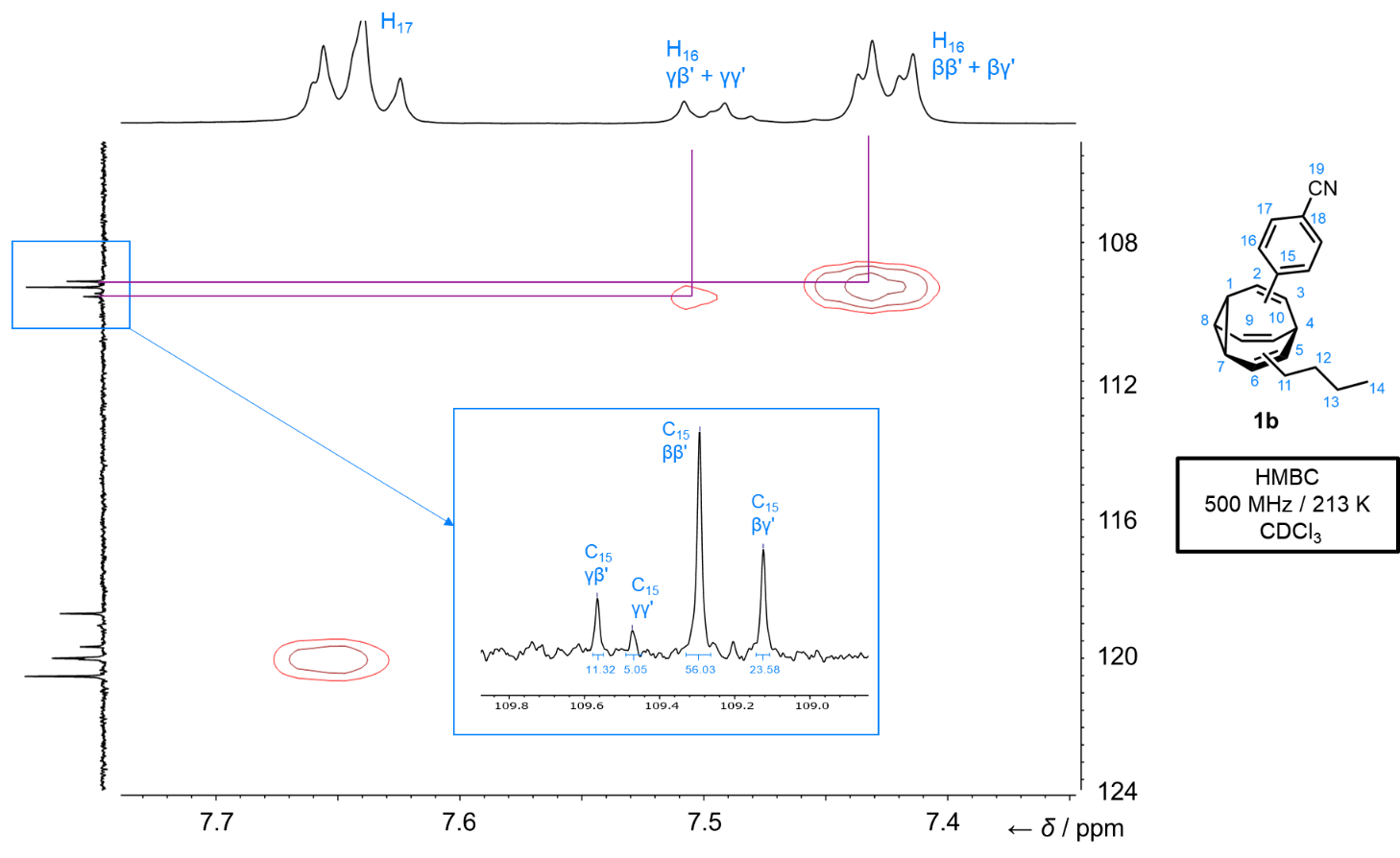


**Figure S21**. Partial HMBC spectra of **1b**.

H_16_ is also observed to correlate with another C atom within the HMBC spectrum. This is presumably an olefinic C on the bridge attached to the aryl substituent (C_3_ for isomer ββ' and β,γ', C_2_ for isomer γ,β' and γ,γ') (Figure S22). Assignment of the C that shares the C=C and its attached H (H_2_ for isomer ββ' and β,γ', H_3_ for isomer γ,β' and γ,γ') was again possible using HMBC correlations. Note that the correlations of isomer γ,β' and γ,γ' are sometimes not observable in 2D spectra due to their low intensity, and in that case peaks are determined solely by the assumed ratio of integrals with isomer ββ' and β,γ'.


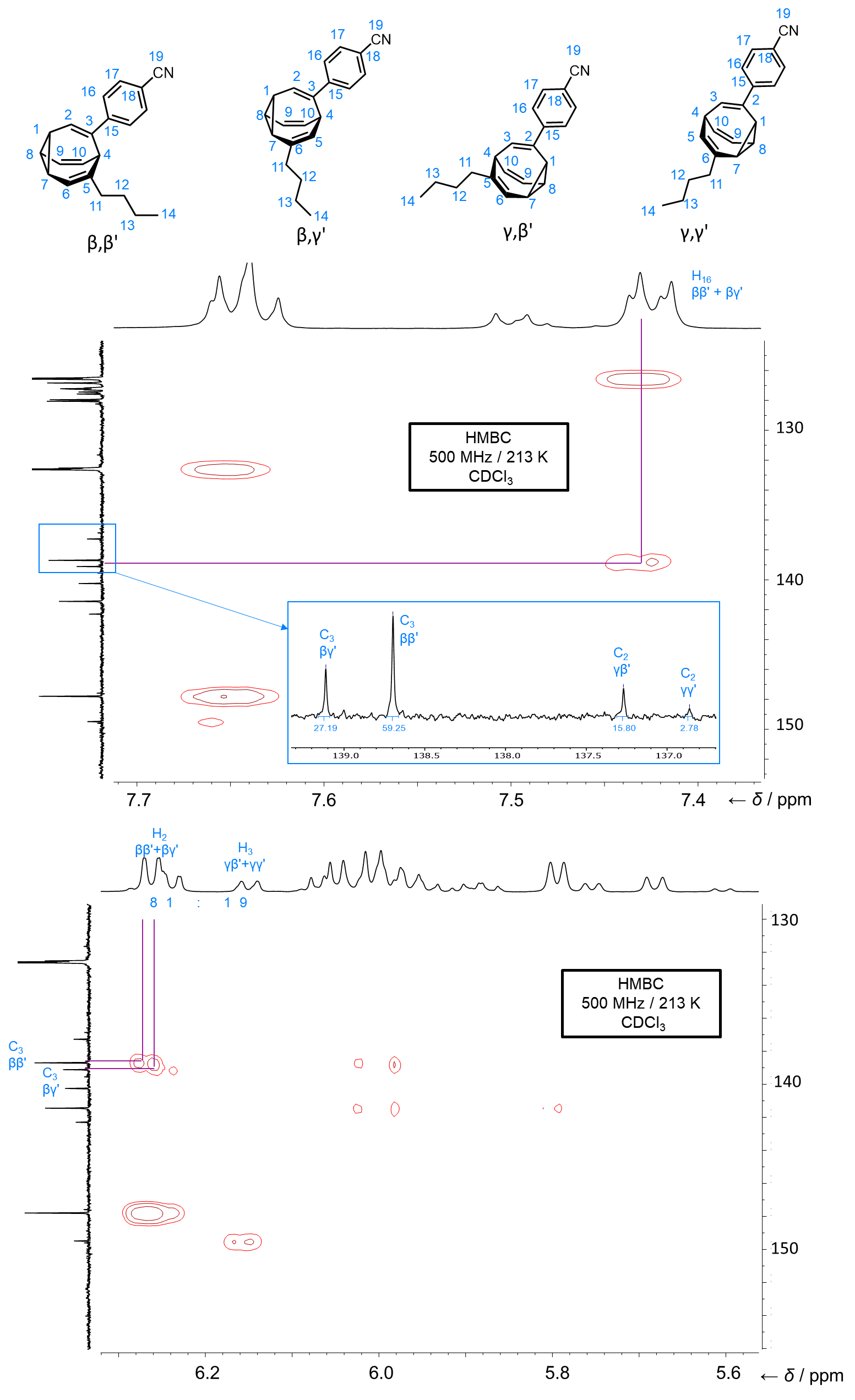


**Figure S22**. Partial HMBC spectra of **1b**.

With H_2_ and H_3_ assigned, the COSY spectrum allowed for the determination of their neighbouring Hs, as H_2_ correlates to H_1_ in isomer β,β' and β,γ', and H_3_ correlates to H_4_ in isomer γ,β' and γ,γ' (Figure S23).


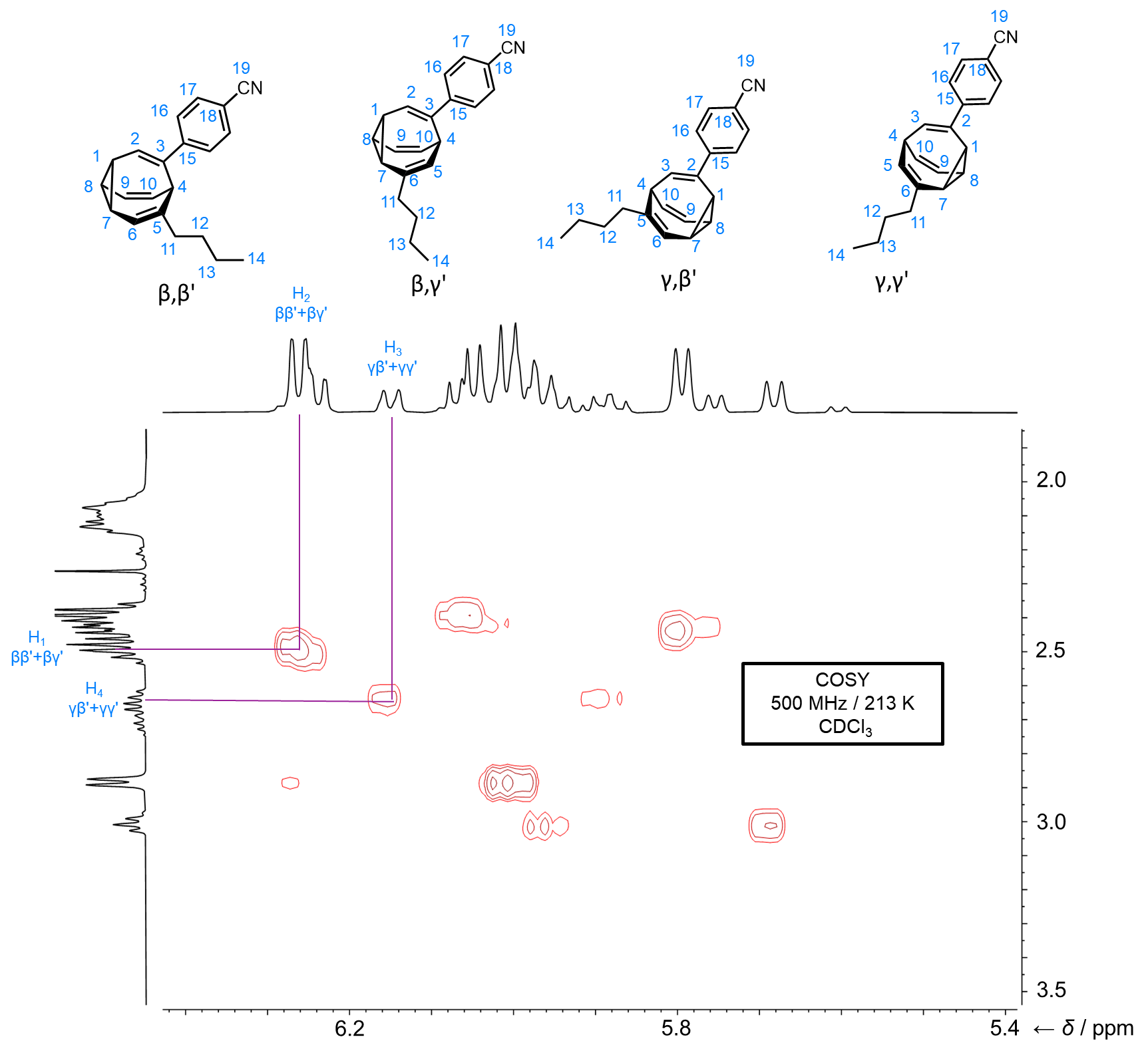


**Figure S23**. Partial COSY spectra of **1b**.

The Hs on the butyl substituents are determined using integrals in and chemical shifts in the ^1^H spectrum. Two signals arising from H_14_ were observed, with the signal at 0.9 ppm being assigned to the β,β' isomer and the signal at 0.75 ppm being assigned to a mixture of β,γ', γ,β' and γ,γ' based on the relative integrals of the two signals (Figure S24).


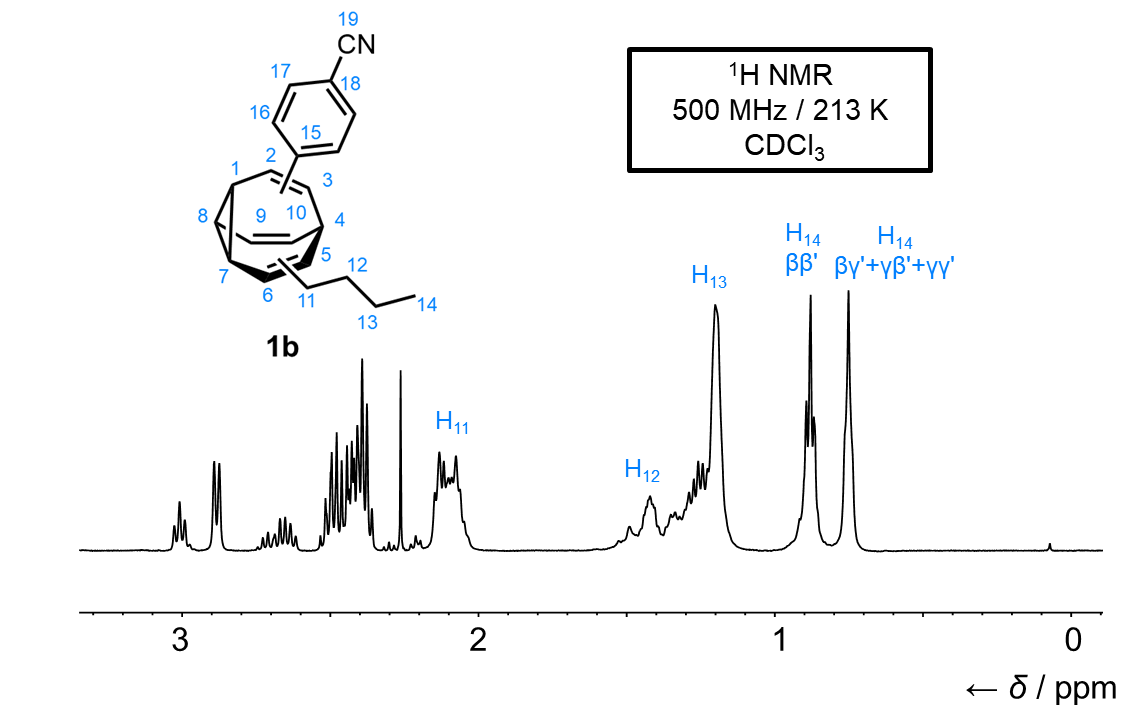


**Figure S24**. Partial ^1^H NMR spectrum of **1b**.

Determination of H_11_ allowed assignment of the olefinic C atoms attached to the butyl substituent (C_5_ for isomer ββ' and γ,β', C_6_ for isomer β,γ' and γ,γ') by HMBC (Figure S25), as well as their attached Hs (H_6_ for isomer ββ' and γ,β’, H_5_ for isomer β,γ' and γ,γ').


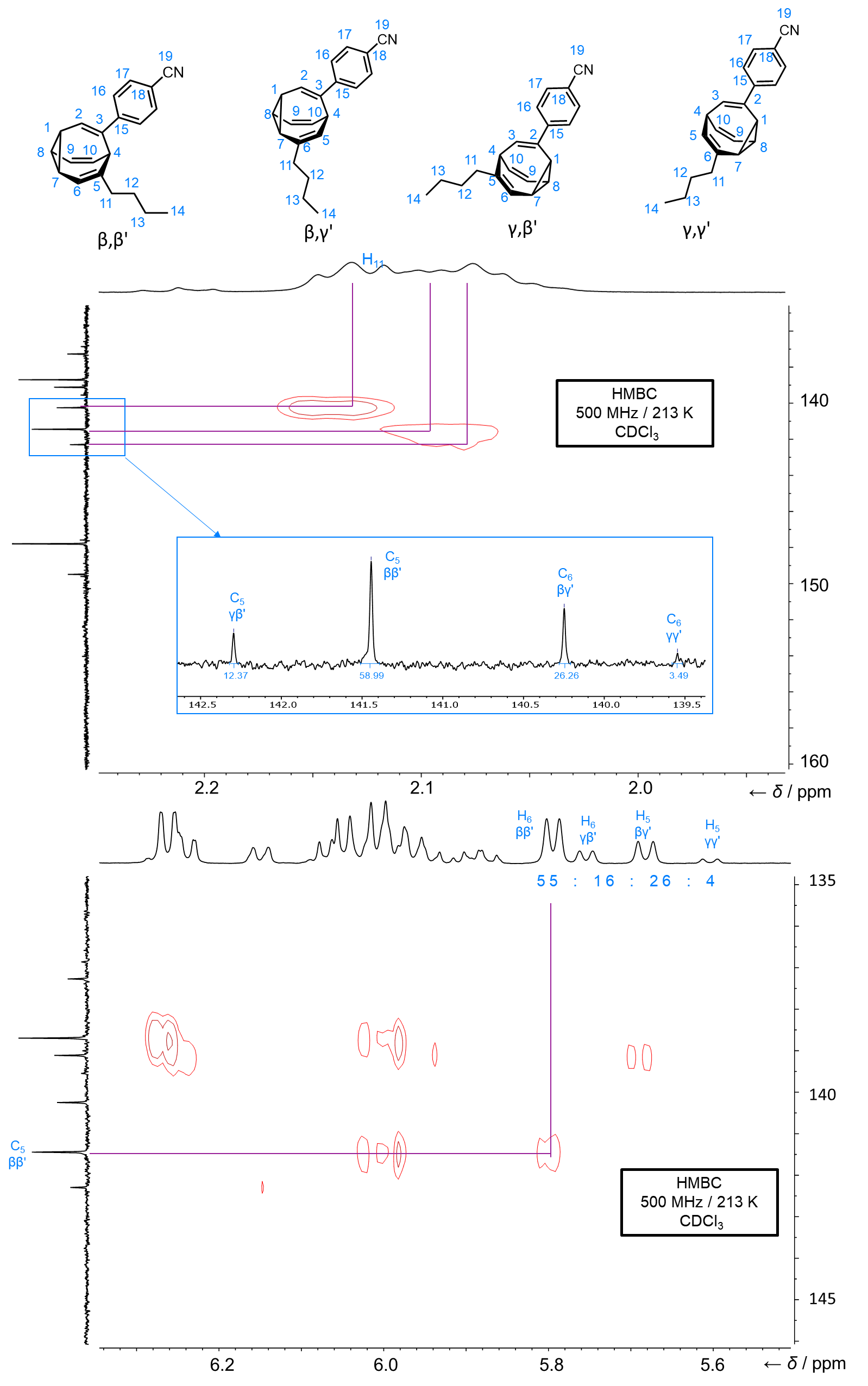


**Figure S25**. Partial HMBC spectrum of **1b**.

H_4_ of isomer β,β' was assigned by virtue of this signal’s correlation with both C_3_ and C_5_ in the HMBC spectrum (Figure S26).


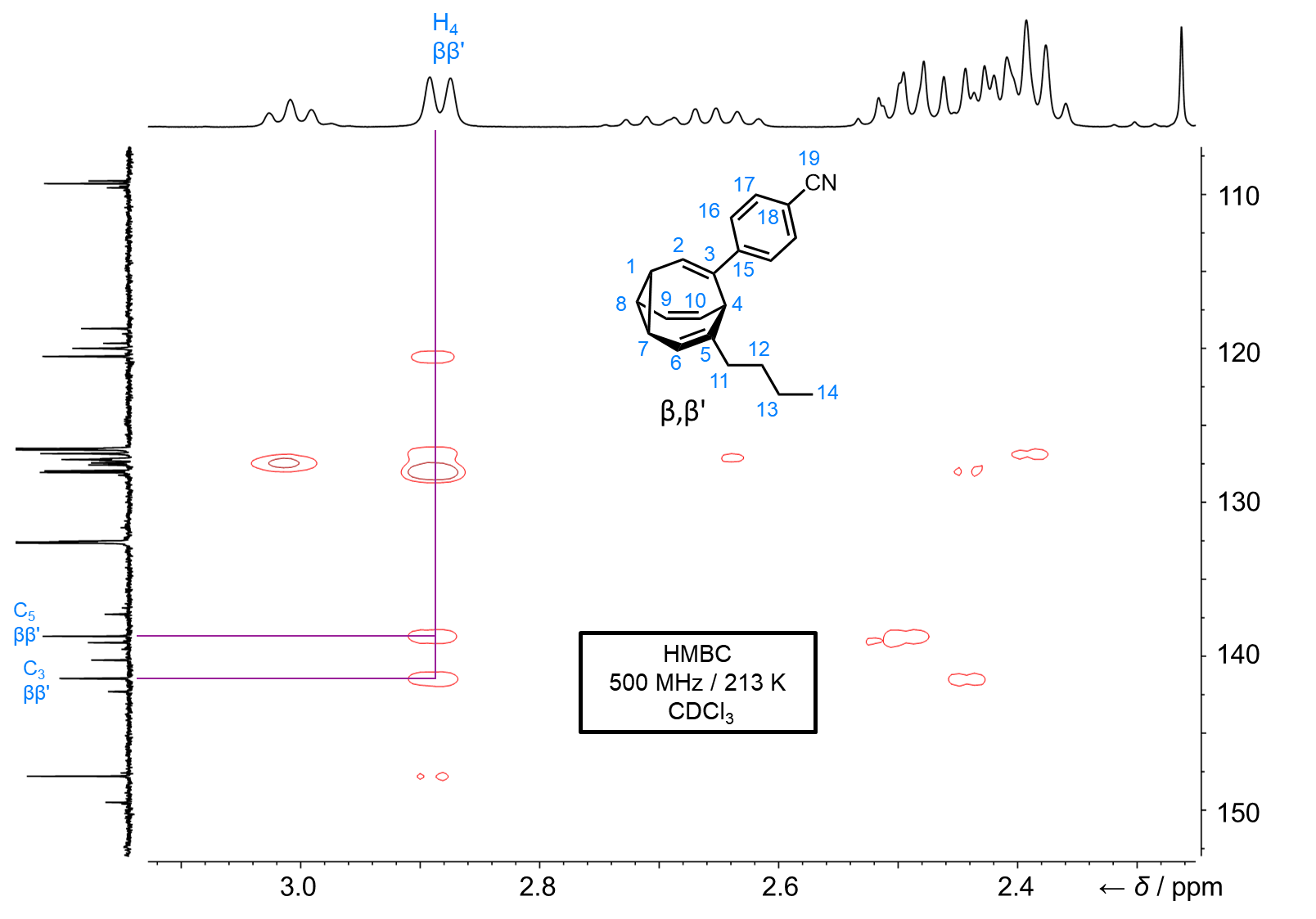


**Figure S26**. Partial HMBC spectrum of **1b**.

H_4_ of the other three isomers are then determined using the COSY spectrum, as this proton would be expected to correlate to H_3_ in isomer γ,β' and γ,γ' and H_5_ in isomer β,γ' (Figure S27).


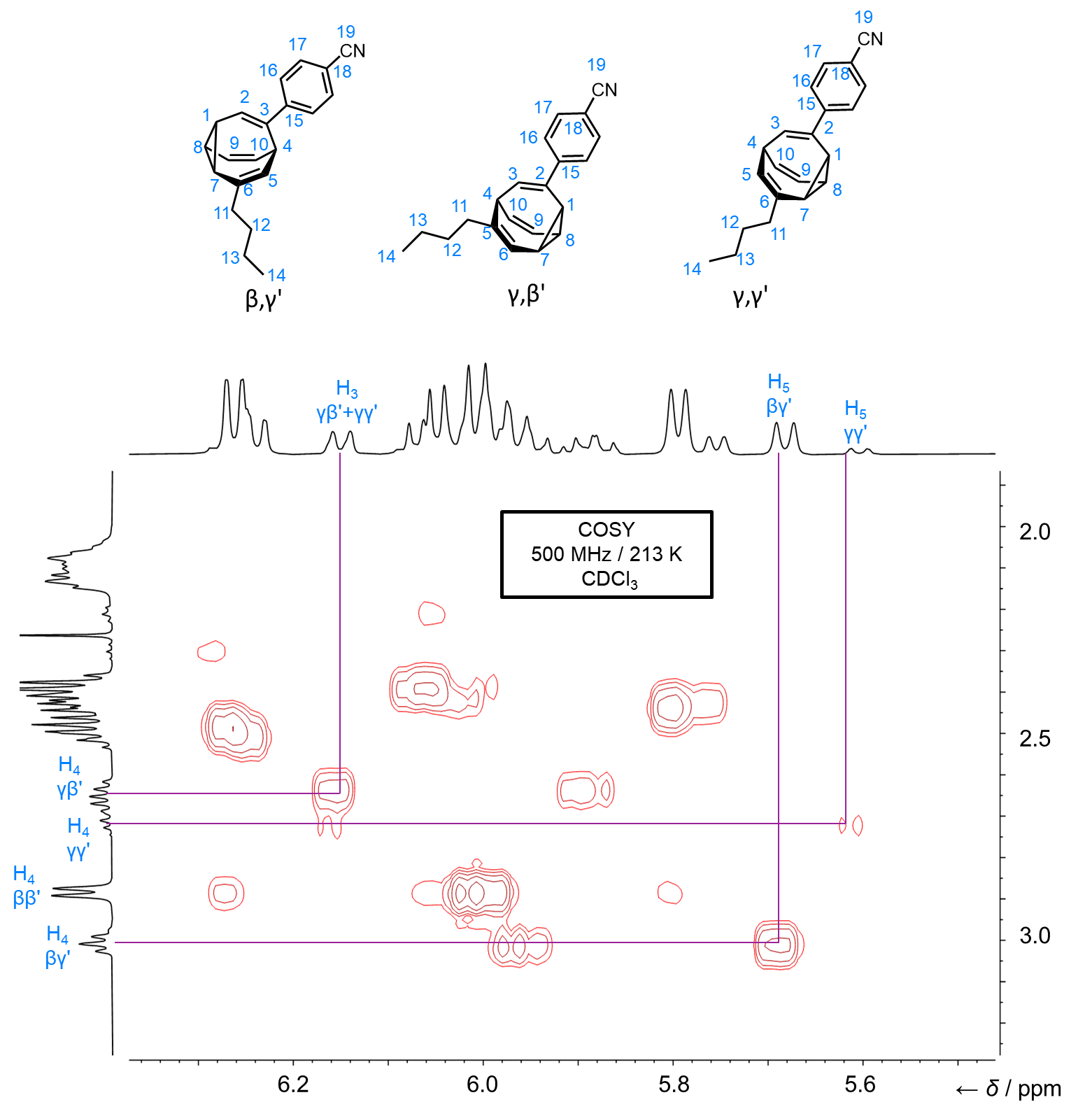


**Figure S27**. Partial HMBC spectrum of **1b**.

H_10_ of all isomers could then be determined via the correlations with H_4_ in the COSY spectrum (Figure S28). After the determination of H_2/3/5/6/10_, the remaining set of peaks in the olefin region (6.09－6.03 ppm) are assigned to H_9_ from each isomer by elimination.


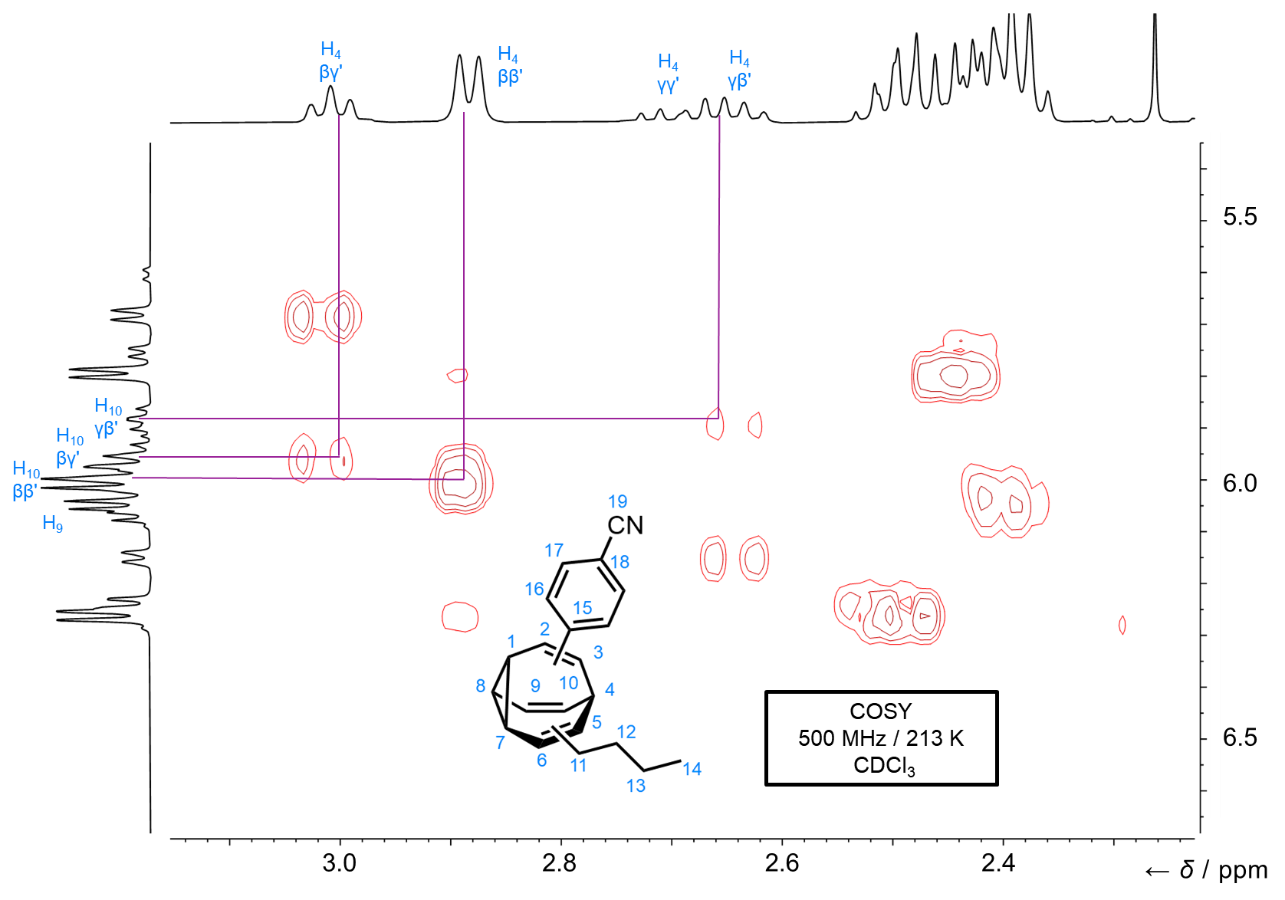


**Figure S28**. Partial COSY spectrum of **1b**.

After determination of all the other ^1^H peaks, the remaining peaks at 2.55 – 2.34 ppm were assigned to be a mixture of signals corresponding to H_1/7/8_. The three positions on the cyclopropane ring could not be distinguished from each other as their correlations are overlapping in the 2D spectra. A final assignment of all signals in the ^1^H NMR at low temperature is shown in Figure S29.


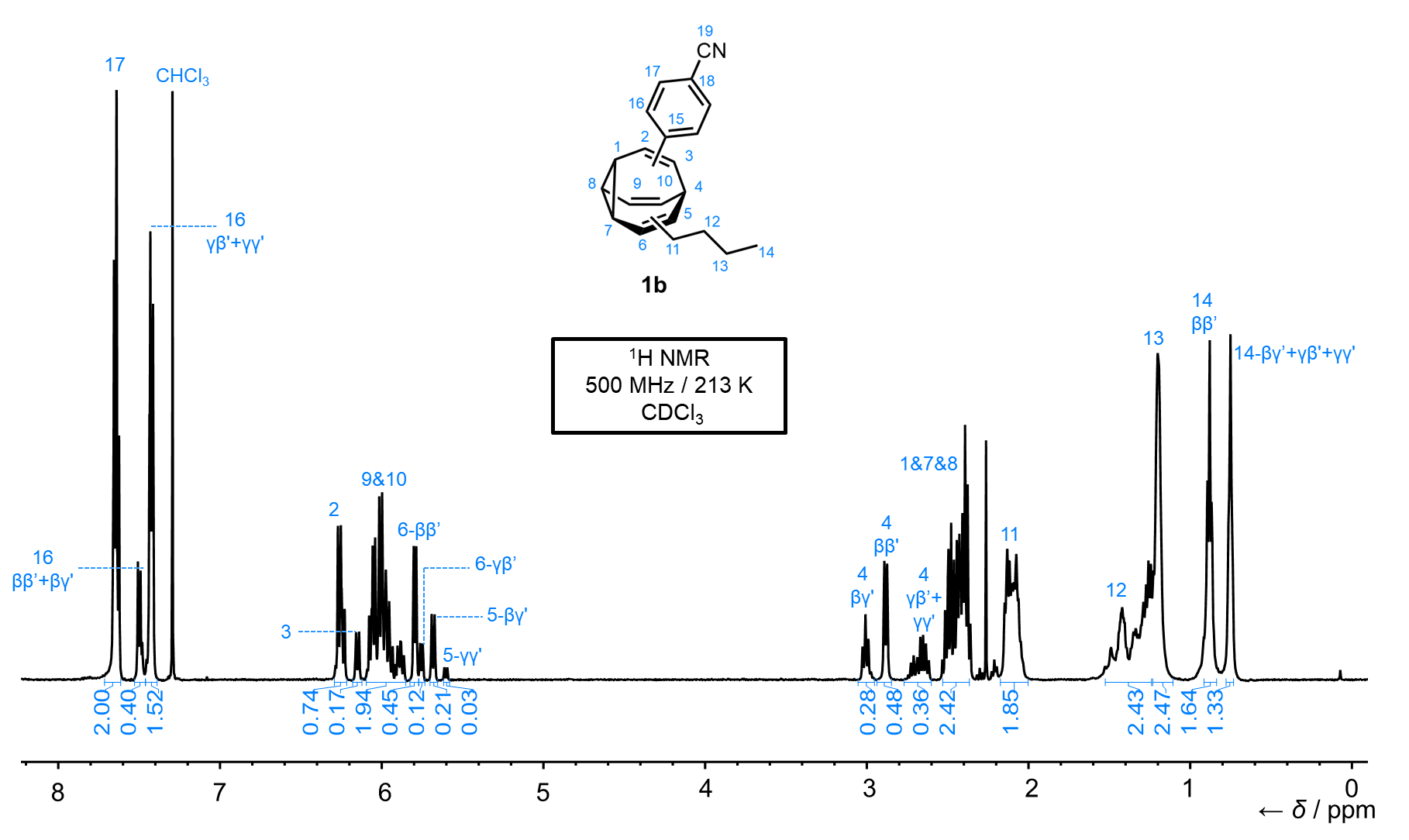


**Figure S29**. Fully assigned ^1^H NMR spectrum of **1b**.

Finally, C_18_ and C_19_ are distinguished from each other by their chemical shifts and by HMBC, where C_18_ correlates to both H_16_ and H_17_ while C_19_ only correlates to H_17_ (Figure S30). Two signals were observed for C_19_ of the two most populous isomers, with the other two signals not being visible due to the low intensity of this quaternary C.


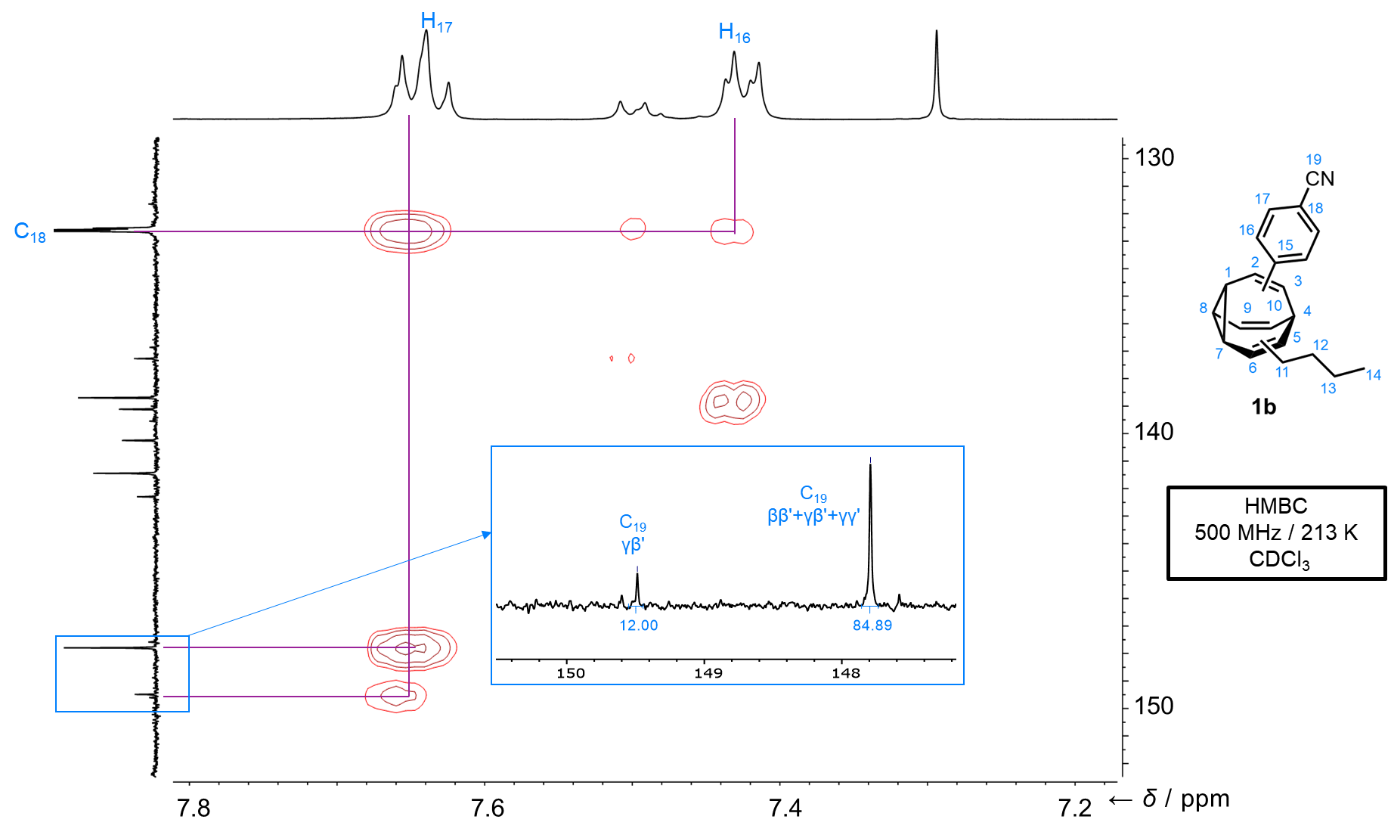


**Figure S30**. Partial HMBC spectrum of **1b**.

The remaining ^13^C peaks are determined by looking for their corresponding ^1^H peaks in the HSQC spectrum to complete the assignment of bullvalene **1b** (Figure S31). The ^1^H and ^13^C NMR spectra of bullvalene **1a** are assigned following the same procedure. Using an initial assumption that four isomers are present (β,β'; β,γ'; γ,β' and γ,γ'), it was possible to produce a consistent set of assignments for the signals present in the low temperature ^1^H and ^13^C NMR spectra using 2D NMR spectroscopy. These experiments, in conjunction with previous reports on disubstituted bullvalenes and our DFT calculations, lend confidence to our assignment of the isomer distribution in the solution state.


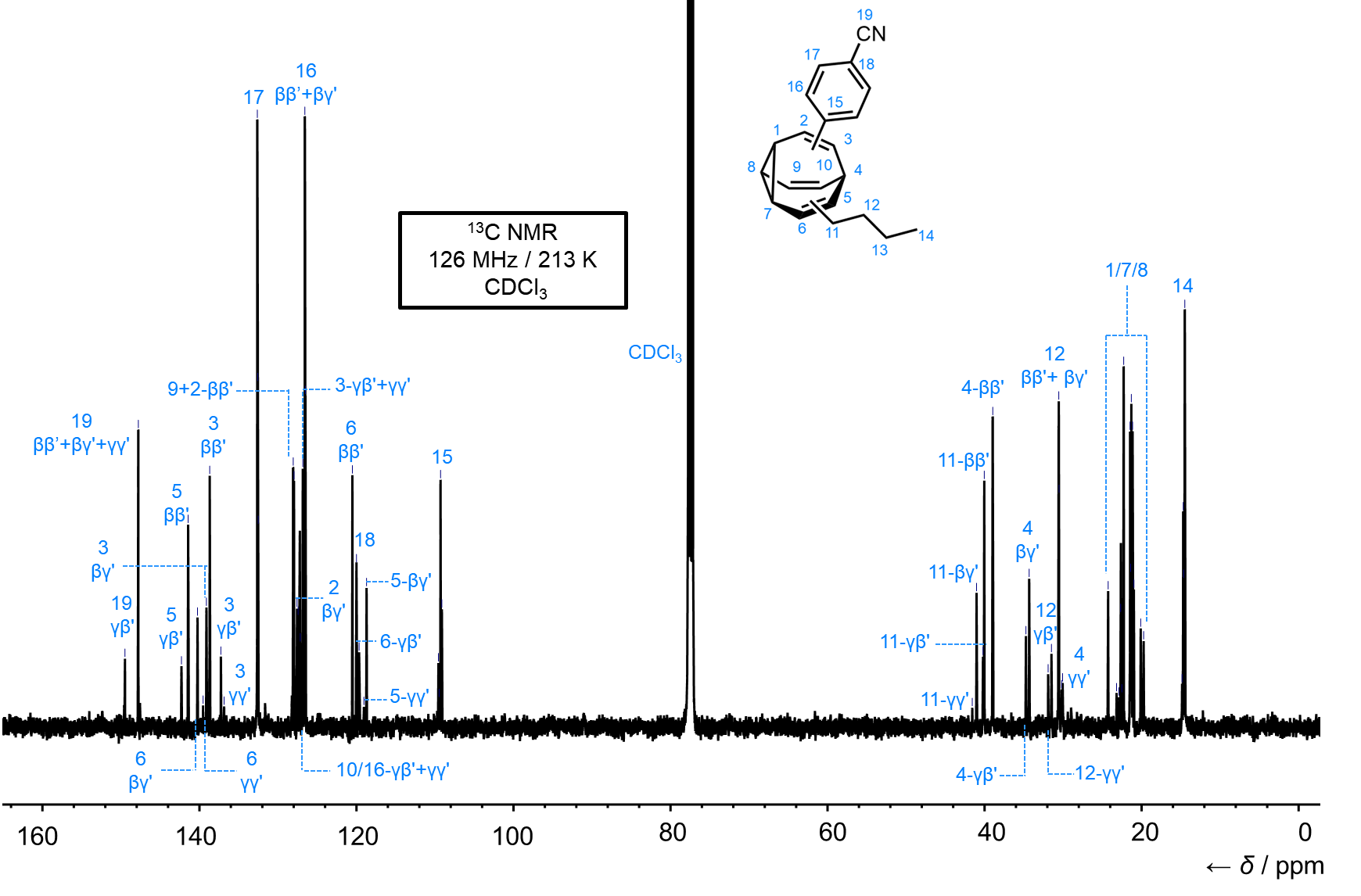


**Figure S31**. Fully assigned ^13^C NMR spectrum of **1b**.

The peaks of synthesized BDTs **2** and **4** were also assigned using 2D NMR spectra obtained at 298 K. For BDT **2a**, the aromatic peaks H_17/18_ and alkyl peaks H_11_–H_15_ were easily determined by chemical shifts, and their corresponding ^13^C peaks were assigned by HSQC spectrum (Figure S32). H_11_ appears as two distinct peaks due to the different environments of the axial and equatorial hydrogen atoms. The equatorial hydrogen (H_11E_) exhibits a higher chemical shift than the axial hydrogen (H_11A_), attributed to the deshielding effect of the aryl substituent.


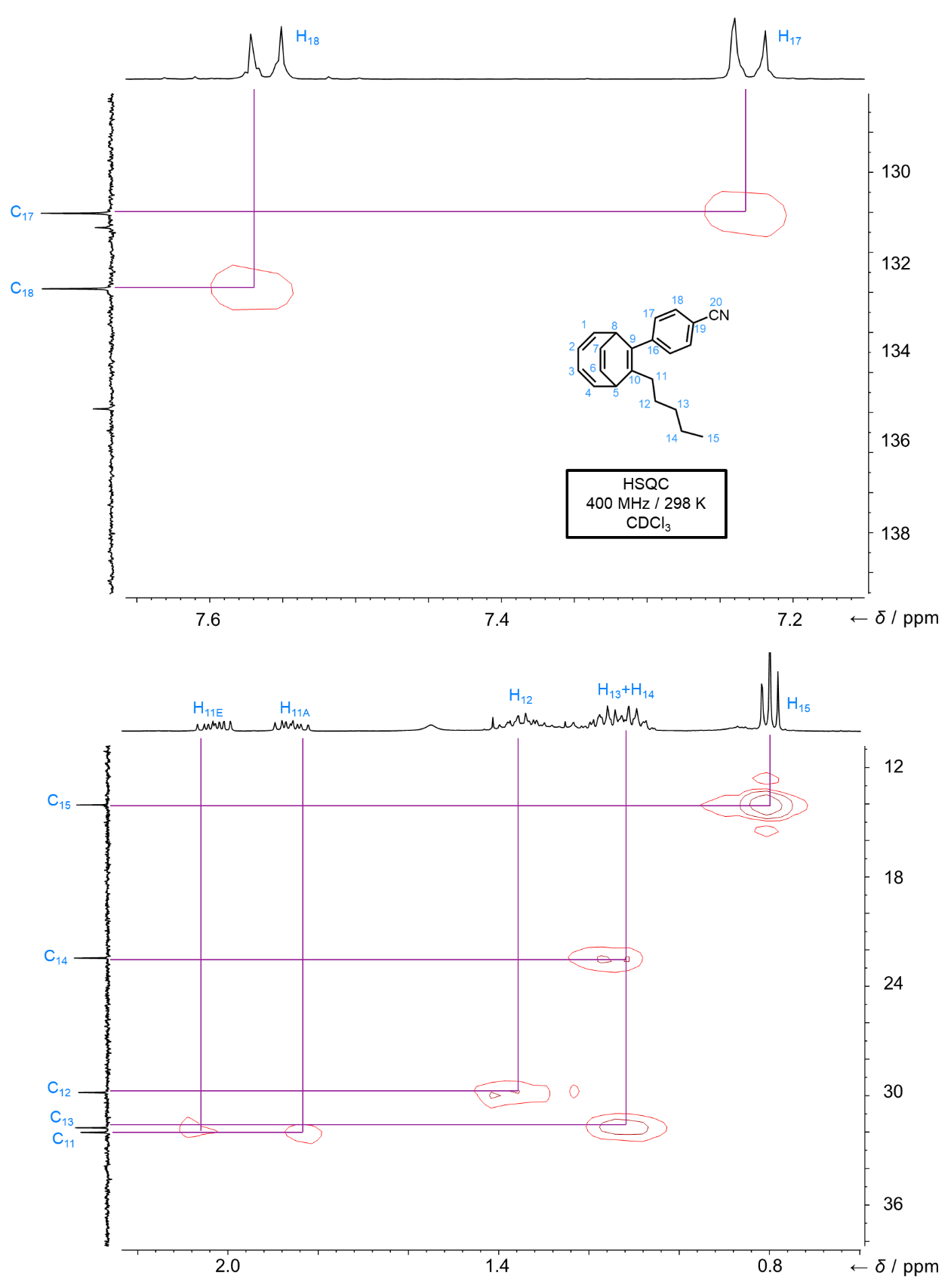


**Figure S32**. Partial HSQC spectra of **2a**.

C_16_, C_19_ and C_20_ are determined through their correlations with H_17_ and H_18_ in the HMBC spectrum, respectively (Figure S33).


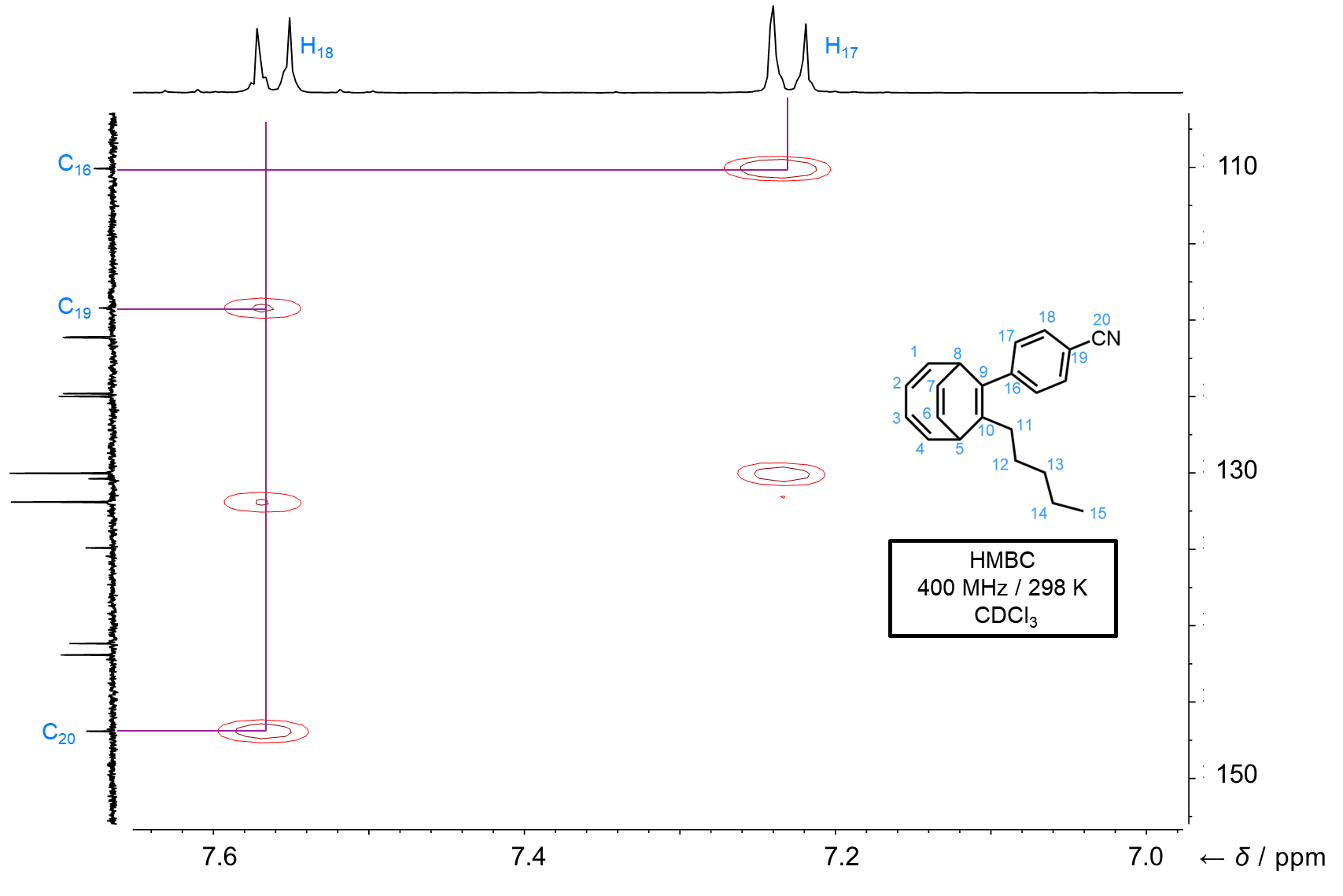


**Figure S33**. Partial HMBC spectrum of **2a**.

C_9_ and C_10_ are determined through their correlations with H_11_ in the HMBC spectrum (Figure S34). C_9_ exhibits a higher chemical shift due to the deshielding effect of the aryl substituent.


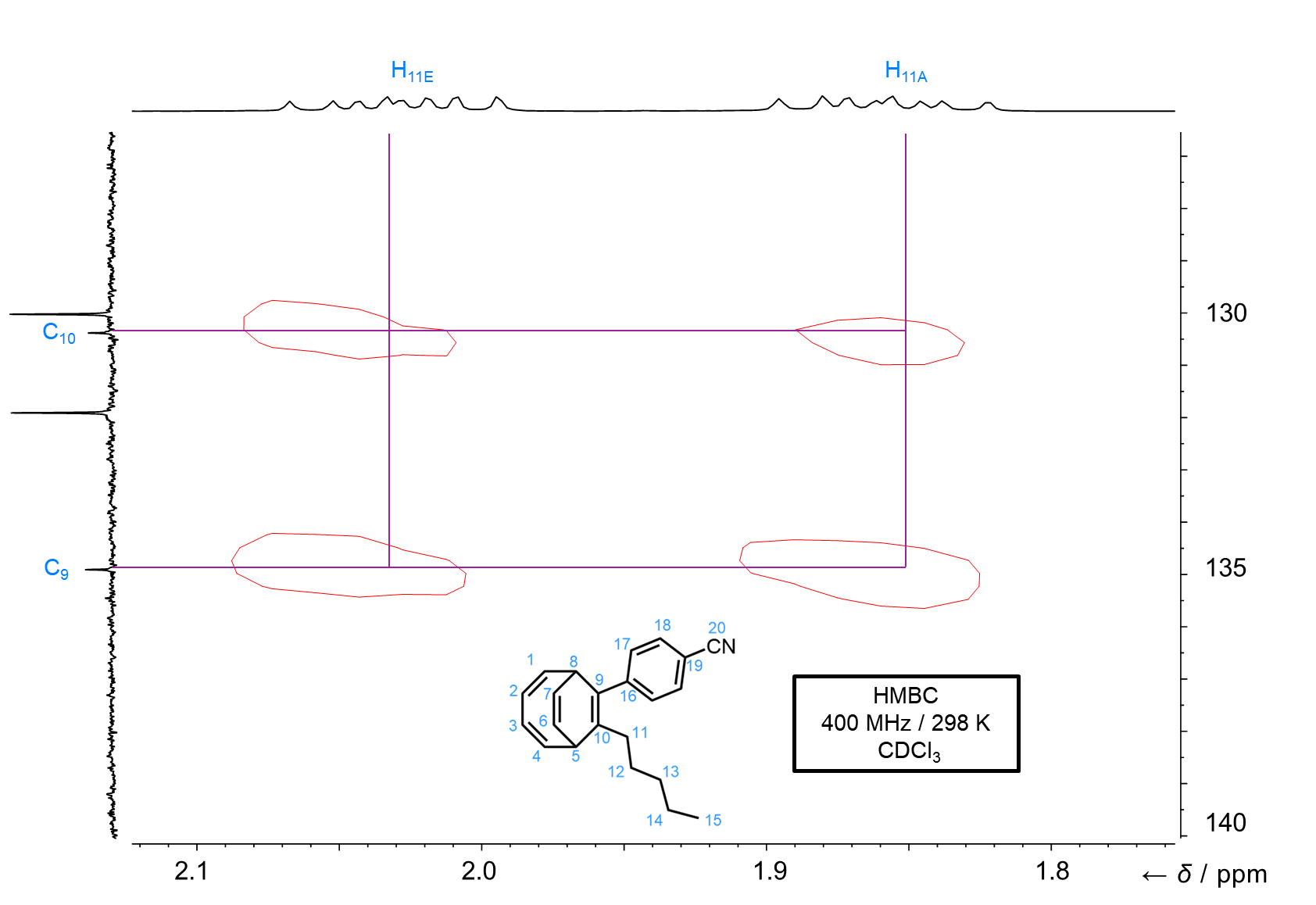


**Figure S34**. Partial HMBC spectrum of **2a**.

H_5_ and H_8_ are determined through correlations with C_9_ and C_10_ in the HMBC (Figure S35).


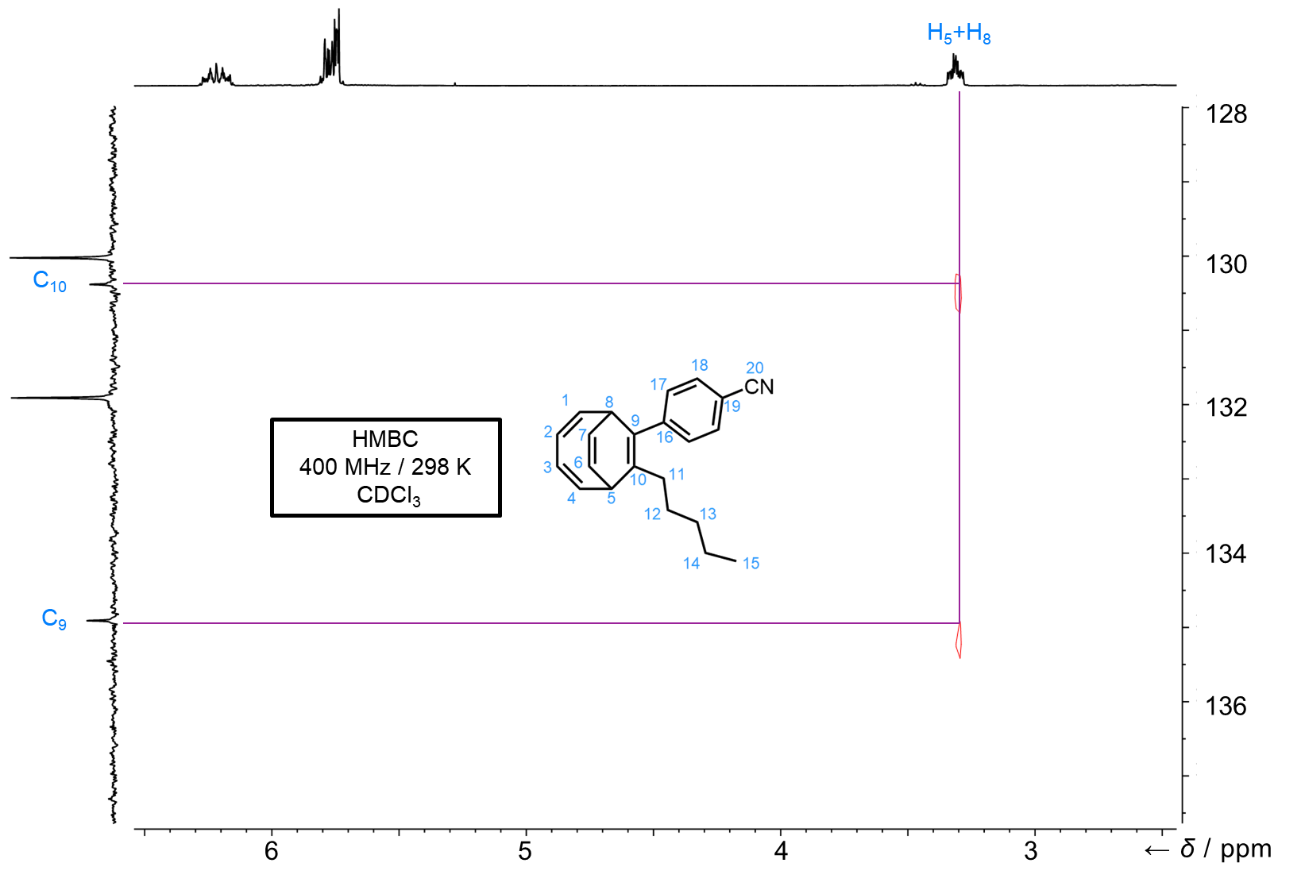


**Figure S35**. Partial HMBC spectrum of **2a**.

Upon determination of C_5_ and C_8_ through HSQC, H_1/4/6/7_ were determined through their correlations in the HMBC, then the remaining H peaks at around 6.2 ppm belongs to H_2/3_ (Figure S36). A final assignment of all signals of **2a** in the ^1^H NMR is shown in Figure S14.


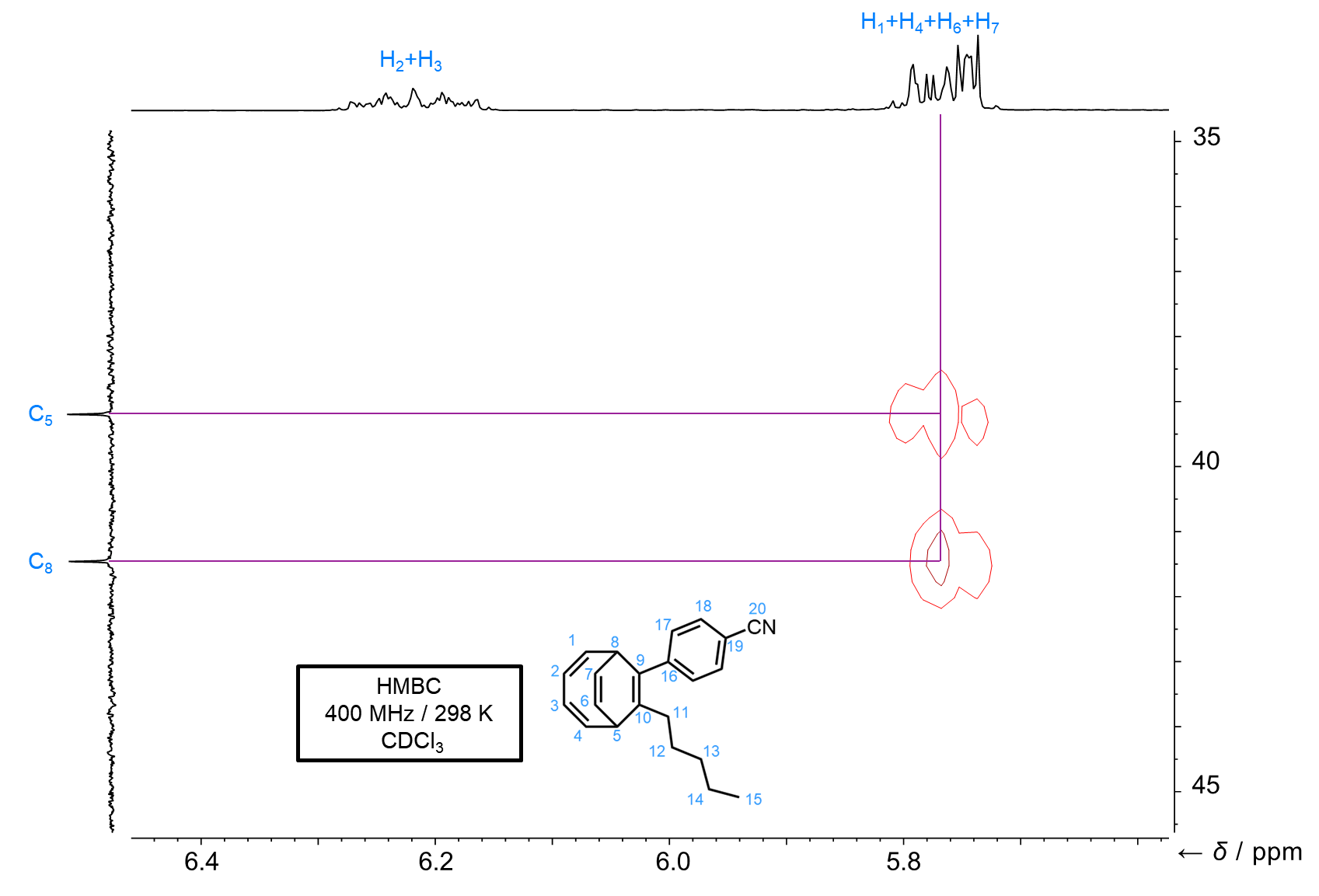


**Figure S36**. Partial HMBC spectrum of **2a**.

Though the peaks of H_1/4/6/7_ are mixed together, the correlations with C_2/3_ in the HMBC suggest that H_1/4_ roughly exhibit the higher chemical shifts while H_6/7_ exhibit the lower (Figure S37).


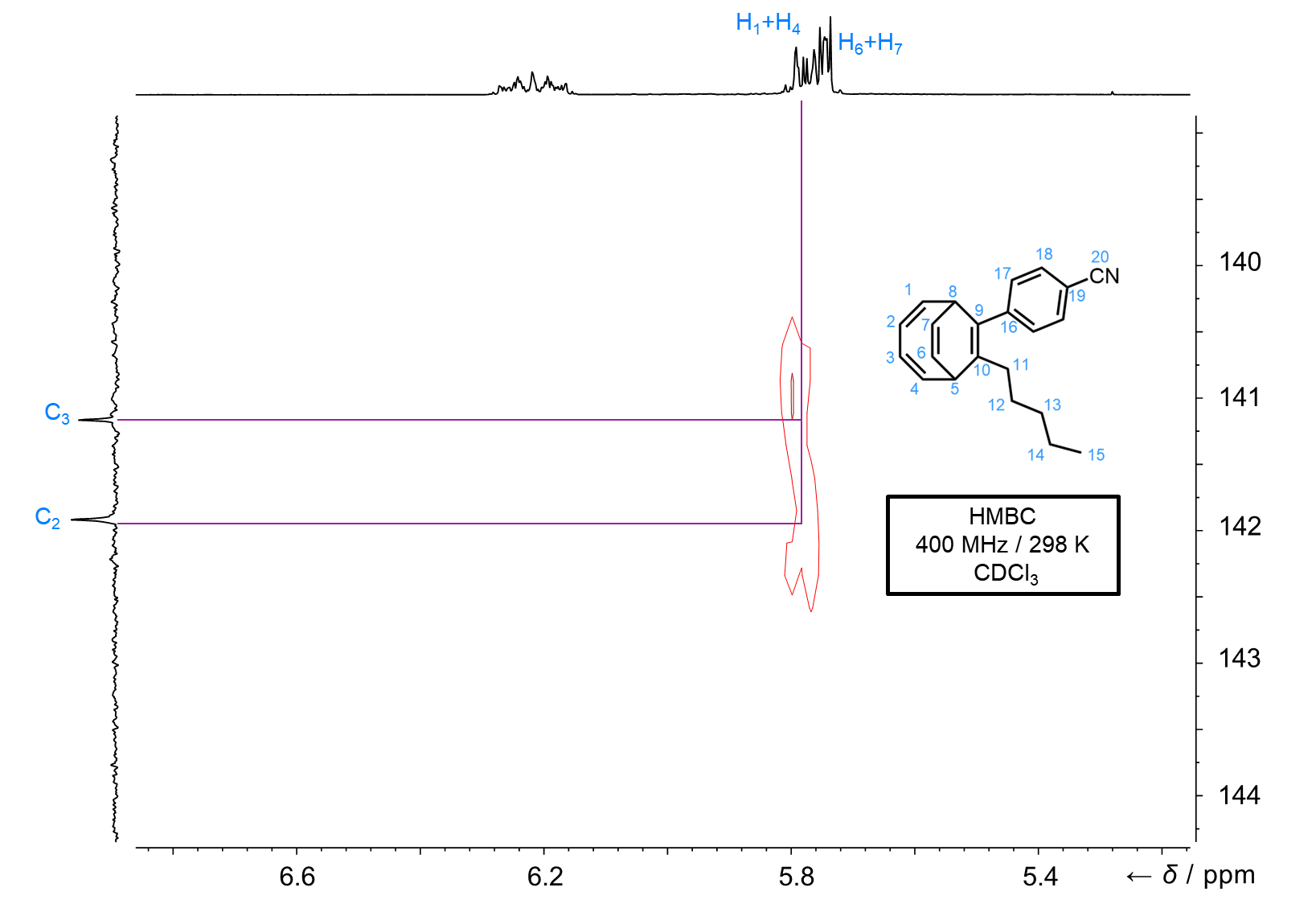


**Figure S37**. Partial HMBC spectrum of **2a**.

Through the slight difference in the HSQC, C_1/4_ and C_6/7_ can be distinguished. A final assignment of all signals of **2a** in the ^1^H NMR is shown in Figure S15. The ^1^H and ^13^C spectra of BDT **2b** were assigned following the same procedure.


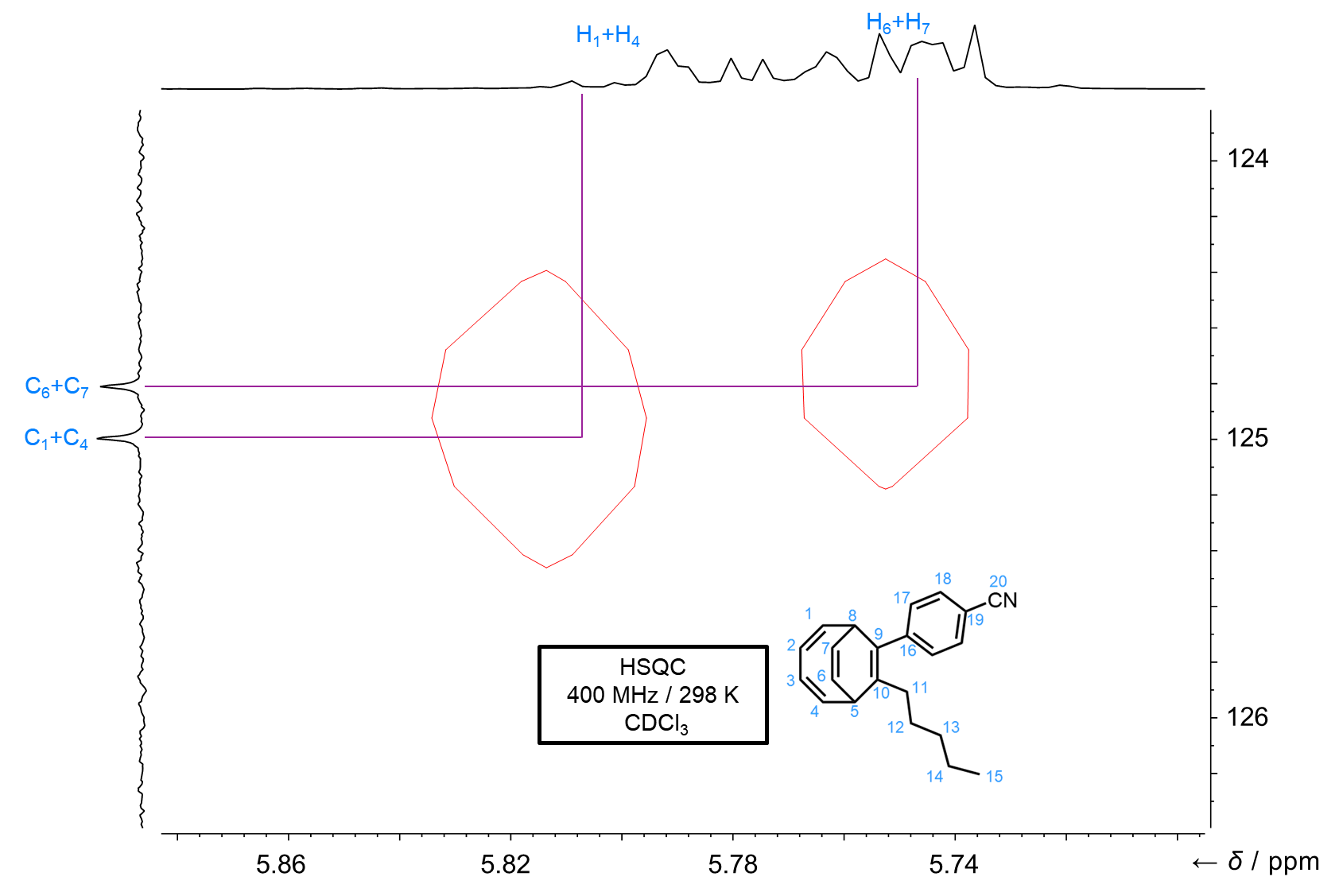


**Figure S38**. Partial HSQC spectrum of **2a**.

For BDT **4a**, the peaks H_17/18_ and H_11_–H_15_ were easily determined by chemical shifts, and their corresponding ^13^C peaks were assigned by HSQC spectrum. C_18_ exhibits a lower chemical shift than C_17_ due to the shielding effect of the alkyl chain (Figure S39).


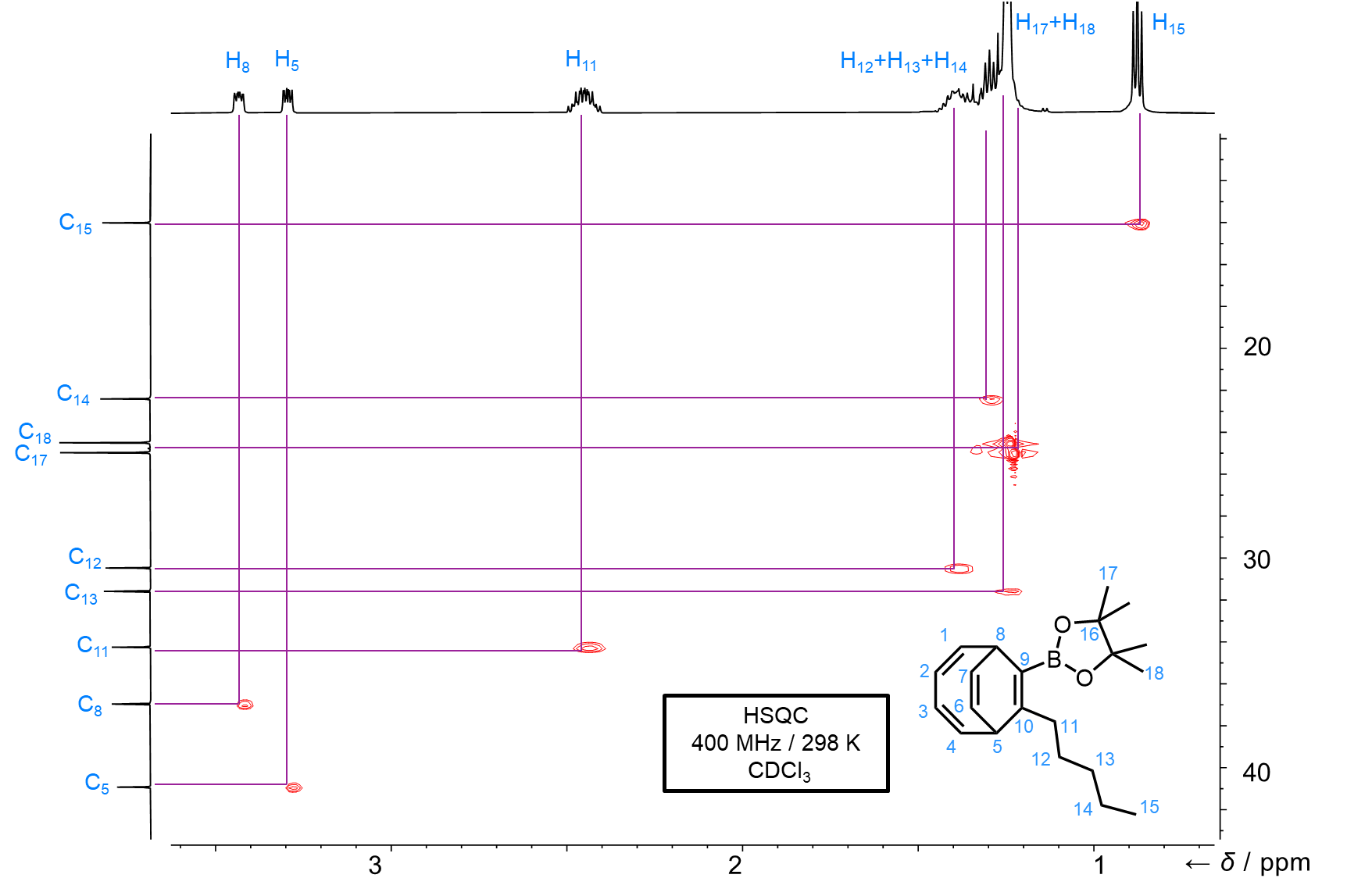


**Figure S39**. Partial HSQC spectrum of **4a**.

C_10_ and C_16_ were determined through correlations with H_11_ and H_17/18_ in the HMBC and no corresponding H atoms in the HSQC (Figure S40).


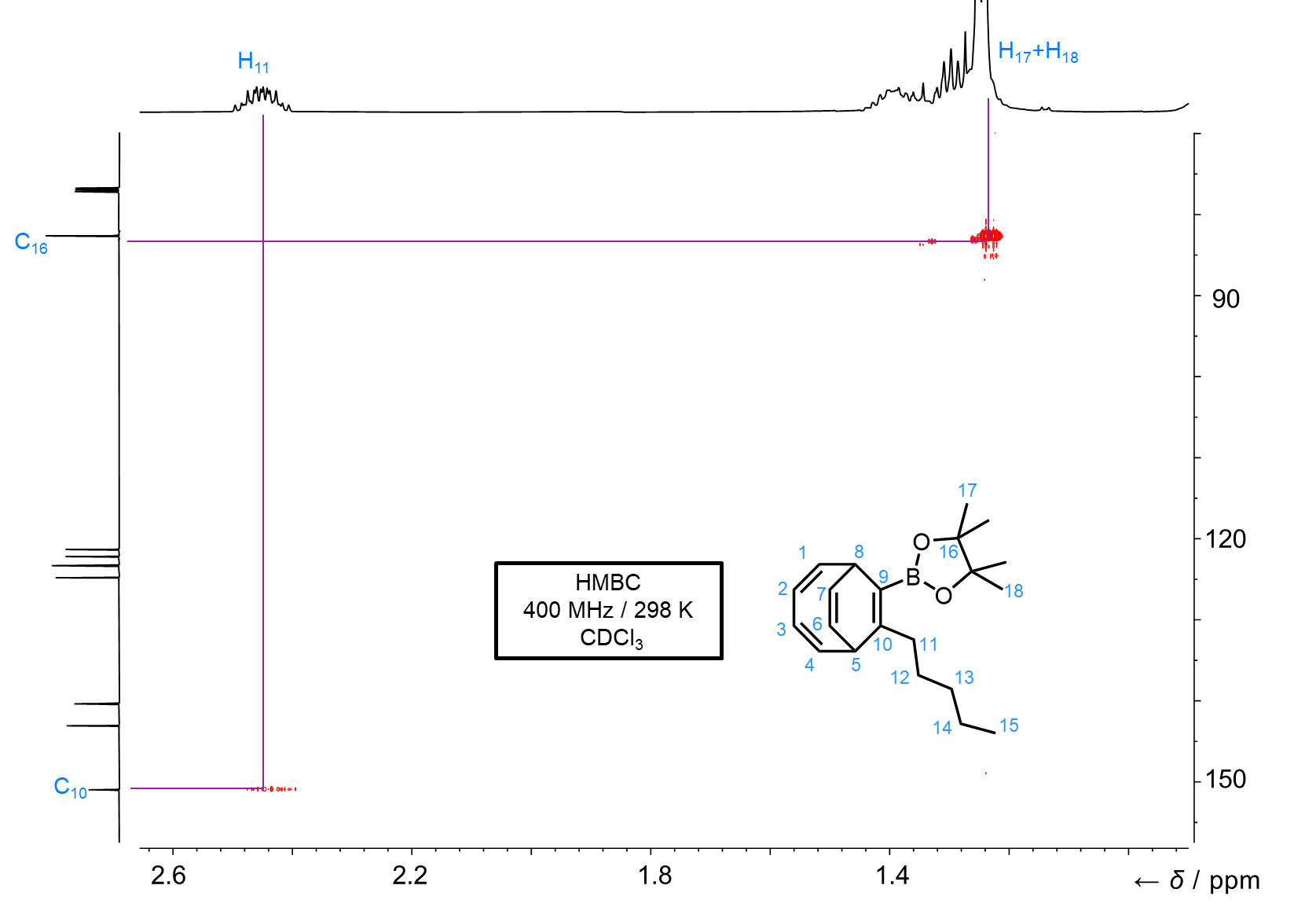


**Figure S40**. Partial HMBC spectrum of **4a**.

C_1/4_ and C_6/7_ were then distinguished through correlations with H_2/3_ in HMBC (Figure S41). Final assignment of all signals of **4a** in the ^1^H and ^13^C NMR are shown in Figure S4 and S5. The ^1^H and ^13^C spectra of BDT **4b** were assigned following the same procedure.


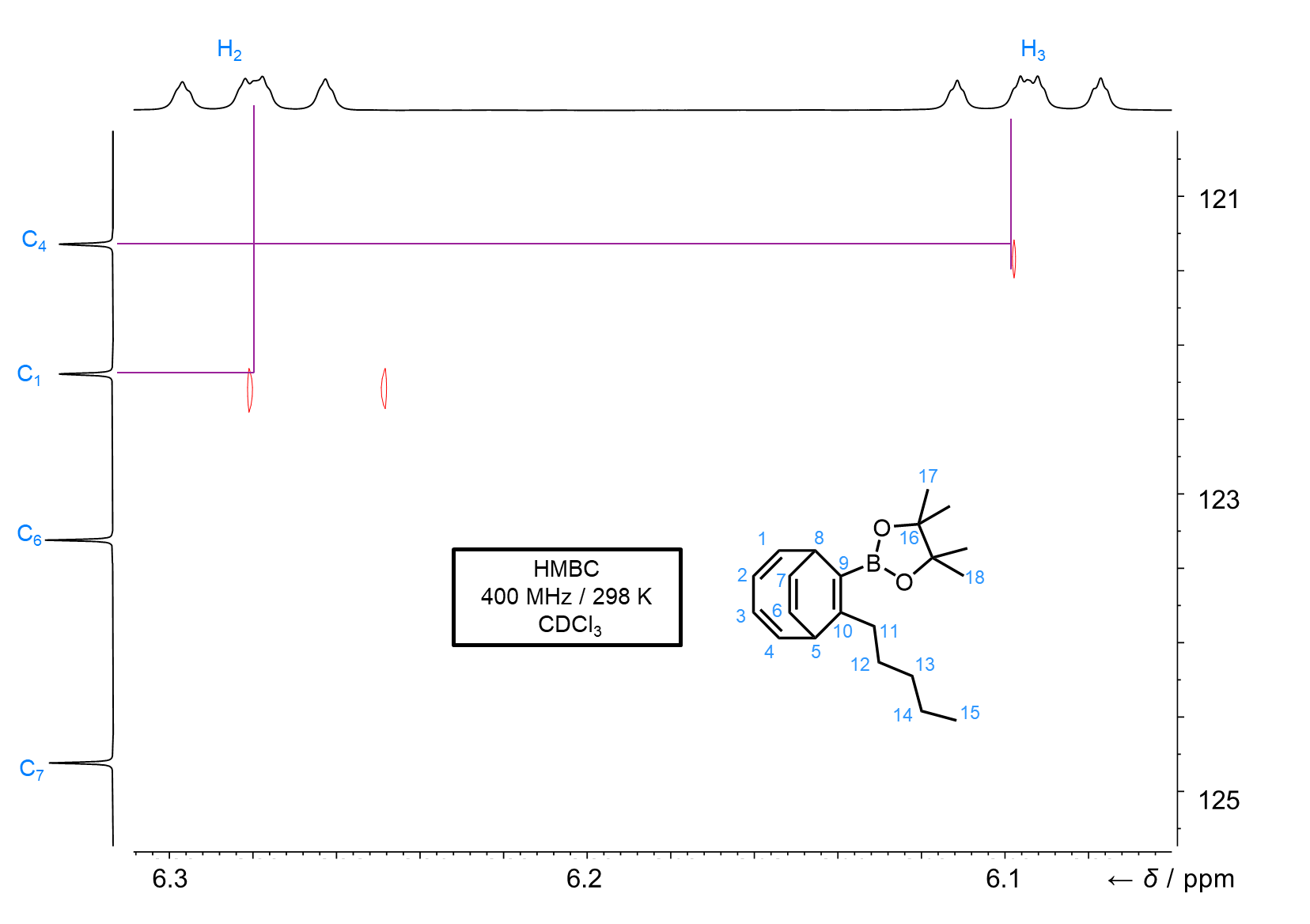


**Figure S41**. Partial HMBC spectrum of **4a**.

6. Solid-State NMR Spectroscopy

Solid-state NMR spectra of neat **1b** were acquired at temperatures above and below its glass transition temperature in order to investigate the effect of phase transition on the isomer distribution (Figure S42). No significant change in shifts or intensities of any signal was observed across the three temperatures, consistent with the fact that there is no major change in isomer population upon formation of the amorphous glass phase. A similar experiment was conducted for **2b** for comparison (Figure S43).


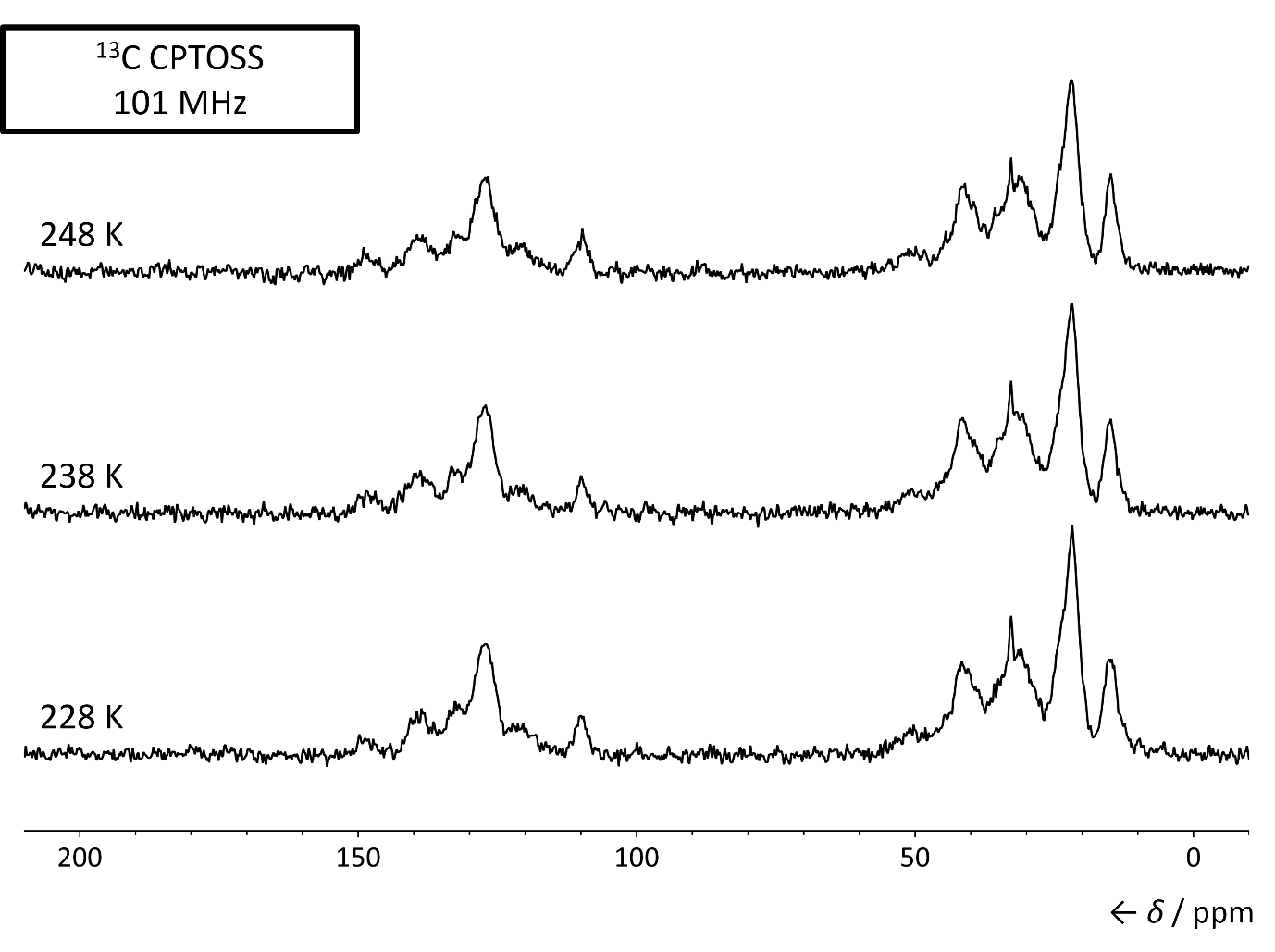


**Figure S42**. Solid state^13^C NMR spectrum of **1b** recorded above and below its *T*_g_.


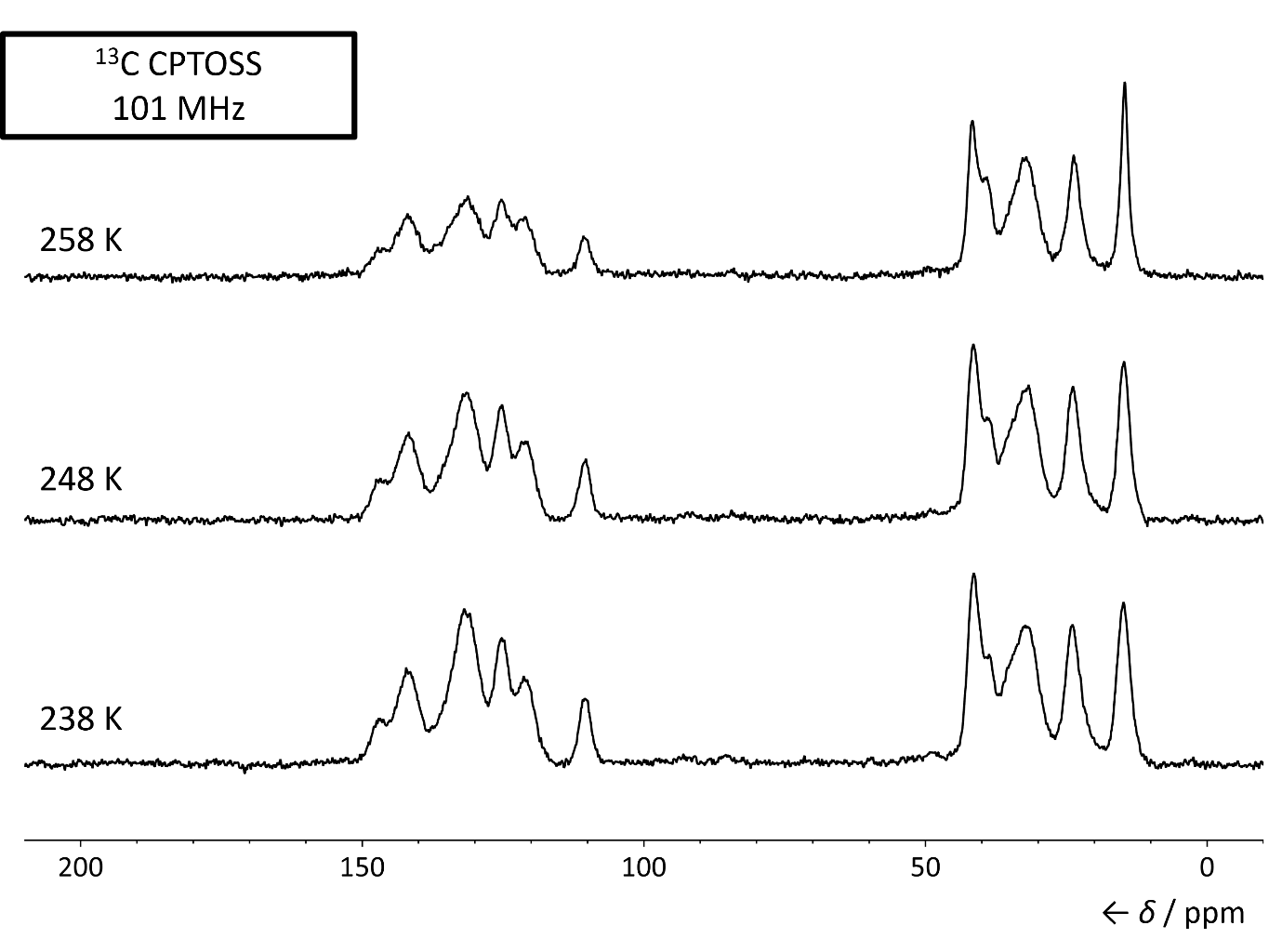


**Figure S43** Solid state^13^C NMR spectrum of **2b** recorded above and below its *T*_g_.

7. Computational modelling

All initial sets of Cartesian coordinates sampling the shapeshifting and conformational isomers were generated *via* the in-house-developed *bullviso* code.^[46]^ *bullviso* is publicly available under the GNU Public License (GPLv3) on GitLab. The α-, β-, γ-, and δ-constitutional isomers of **1b** were generated systematically/exhaustively using *bullviso*; the configurational and conformational isomers were generated according to the experimental torsion distance geometry (ETDG) with ‘basic knowledge’ (+K) embedding approach (ETKDGv3)^[47,48]^ as implemented in RDKit.^[49,50]^ An root mean square deviation (RMSD) filter with a threshold of 0.5 Å was used to prune the initial set of configurational and conformational isomers. All configurational and ten lowest-energy conformational isomers passing the RMSD filter were subsequently (pre-)optimized at the GFN2-xTB^[51,52]^(extended tight binding; xTB) level of theory using *xtb* (v6.4.1).^[53]^ An SCF convergence criterion of 1.0 × 10^−6^ a.u. was used with convergence criteria of 5.0 × 10^−6^ and 1.0 × 10^−3^ a.u. for the energy change and gradient, respectively, in all geometry optimizations. All unique configurational and conformational isomers verified at the GFN2-xTB level of theory were progressed to density functional theory (DFT) geometry optimization.

All DFT geometry optimizations and energy evaluations of **1b**, Me_2_BV, and Me_2_BDT were carried out at the PBE0-D3 level of theory (i.e., with the PBE0^[54-56]^ density functional of Adamo and Barone coupled with the D3^[57]^ dispersion correction of Grimme et al.) using Gaussian 16.^[58]^ All calculations were carried out under the resolution-of-identity (RI) approximation for the Coulomb integrals (RIJONX). A tightened SCF convergence criterion of 1.0 × 10^−9^ a.u. was used in all calculations; convergence criteria of 2.0 × 10^−7^ and 3.0×10^−5^ a.u. were used for the energy change and gradient, respectively, in all geometry optimizations. The def2-SV(P)^[59]^ basis set of Weigend and Ahlrichs was coupled with the def2/J^[60]^ auxilliary basis set; the two were used together throughout. The proper convergence of all geometry optimizations to real minima was verified *via* vibrational frequency inspection.

**Table S1.** Summary of Gibbs energies, *G*_298K_, and relative Gibbs energies, Δ*G*_298K_, for the isomers of **1b**. All values of Δ*G*_298K_ are tabulated relative to the lowest-energy isomer of **1b**. All values are given as evaluated at the PBE0-D3/def2-SV(P) level of theory.

| Isomer Barcode | Isomer | *G*_298K_/ Hartree | Δ*G*_298K_ / kJ mol^−1^ | Isomer population at 298K/ % |
| --- | --- | --- | --- | --- |
| 0000000012 | α,β-**1b** | –865.62743 | 46.99 | 0.0 |
| 0000000021 | β,α-**1b** | –865.63308 | 32.29 | 0.0 |
| 0000000102 | α,𝛾-**1b** | –865.6312 | 37.20 | 0.0 |
| 0000000120 | β,𝛾-**1b** | –865.64083 | 12.15 | 0.3 |
| 0000000201 | 𝛾,α-**1b** | –865.63716 | 21.68 | 0.0 |
| 0000000210 | 𝛾,β-**1b** | –865.63999 | 14.33 | 0.1 |
| 0000001002 | α,δ-**1b** | –865.62974 | 40.98 | 0.0 |
| 0000001020 | β,δ-**1b** | –865.64146 | 10.50 | 0.5 |
| 0000001200 | 𝛾,δ-**1b** | –865.63502 | 27.26 | 0.0 |
| 0000002001 | δ,α-**1b** | – 865.63541 | 26.24 | 0.0 |
| 0000002010 | δ,β-**1b** | –865.63986 | 14.67 | 0.1 |
| 0000002100 | δ,𝛾-**1b** | –865.63473 | 28.02 | 0.0 |
| 0000010020 | β,β'-**1b** | –865.6455 | 0 | 34.0 |
| 0000010200 | 𝛾,β'-**1b** | –865.64388 | 4.23 | 6.2 |
| 0000012000 | δ,β'-**1b** | –865.64084 | 12.11 | 0.3 |
| 0000020010 | β,β'-**1b** | –865.6455 | 0 | 34.0 |
| 0000020100 | β,𝛾'-**1b** | –865.64411 | 3.61 | 7.9 |
| 0000021000 | β,δ'-**1b** | -865.64139 | 10.70 | 0.5 |
| 0000100020 | β,𝛾'-**1b** | -865.64411 | 3.61 | 7.9 |
| 0000100200 | 𝛾,𝛾'-**1b** | –865.64145 | 10.53 | 0.5 |
| 0000102000 | δ,𝛾'-**1b** | –865.63992 | 14.51 | 0.1 |
| 0000200010 | 𝛾,β'-**1b** | –865.64388 | 4.23 | 6.2 |
| 0000200100 | 𝛾,𝛾'-**1b** | –865.64145 | 10.53 | 0.5 |
| 0000201000 | 𝛾,δ'-**1b** | –865.64043 | 13.19 | 0.2 |
| 0001000020 | β,δ'-**1b** | –865.64139 | 10.70 | 0.5 |
| 0001000200 | 𝛾,δ'-**1b** | –865.64043 | 13.19 | 0.2 |
| 0001002000 | δ,δ'-**1b** | –865.63504 | 27.22 | 0.0 |
| 0002000010 | δ,β'-**1b** | –865.64084 | 12.11 | 0.3 |
| 0002000100 | δ,𝛾'-**1b** | –865.63992 | 14.51 | 0.1 |
| 0002001000 | δ,δ'-**1b** | –865.63504 | 27.22 | 0.0 |

**Exit Vector Analysis**

We have previously used exit vector plots to analyse how the relationship between two substituents attached to a BV changes as the BV isomerizes (Figure S44a).^[13]^ The relative orientations of bonds projecting out from the central molecule are defined as the exit vectors, *v*_1_ and *v*_2_ (Figure S44b). Four geometric parameters associated with these vectors are defined as the distance between the functionalized carbon atoms of the scaffold (*r*), the dihedral angle of the vectors (*θ*), and the plane angles of each vector (*φ*_1_ and *φ*_2_). Using the previously reported data for dimethylbullvalene (Me_2_BV), we have compared (Figure S44c) the exit vectors of four isomers of Me_2_BV (corresponding to the eight major isomers of **1**) to the exit vectors of Me_2_BDT (which has analogous connectivity to **2**). Although the BDT gives a short distance between the substituents (Figure S44d) compared to the BV isomers, it gives almost identical plane and dihedral angles (Figures S44e,f) to the β,βʹ-isomer. Therefore, the BDT structure is a useful comparator to the BV. Like the BV, its bicyclic structure is closer to spherical than to the planar phenylene ring it is replacing in the structure of 4CB or 5CB. And the BDT mimics the exit vectors of the major BV isomer. Indeed, the similarity in angles is remarkable considering that one is a 1,2-disubstituted ring system (Me_2_BDT) and the other is a 1,3-disubstituted ring system (β,βʹ-Me_2_BV).


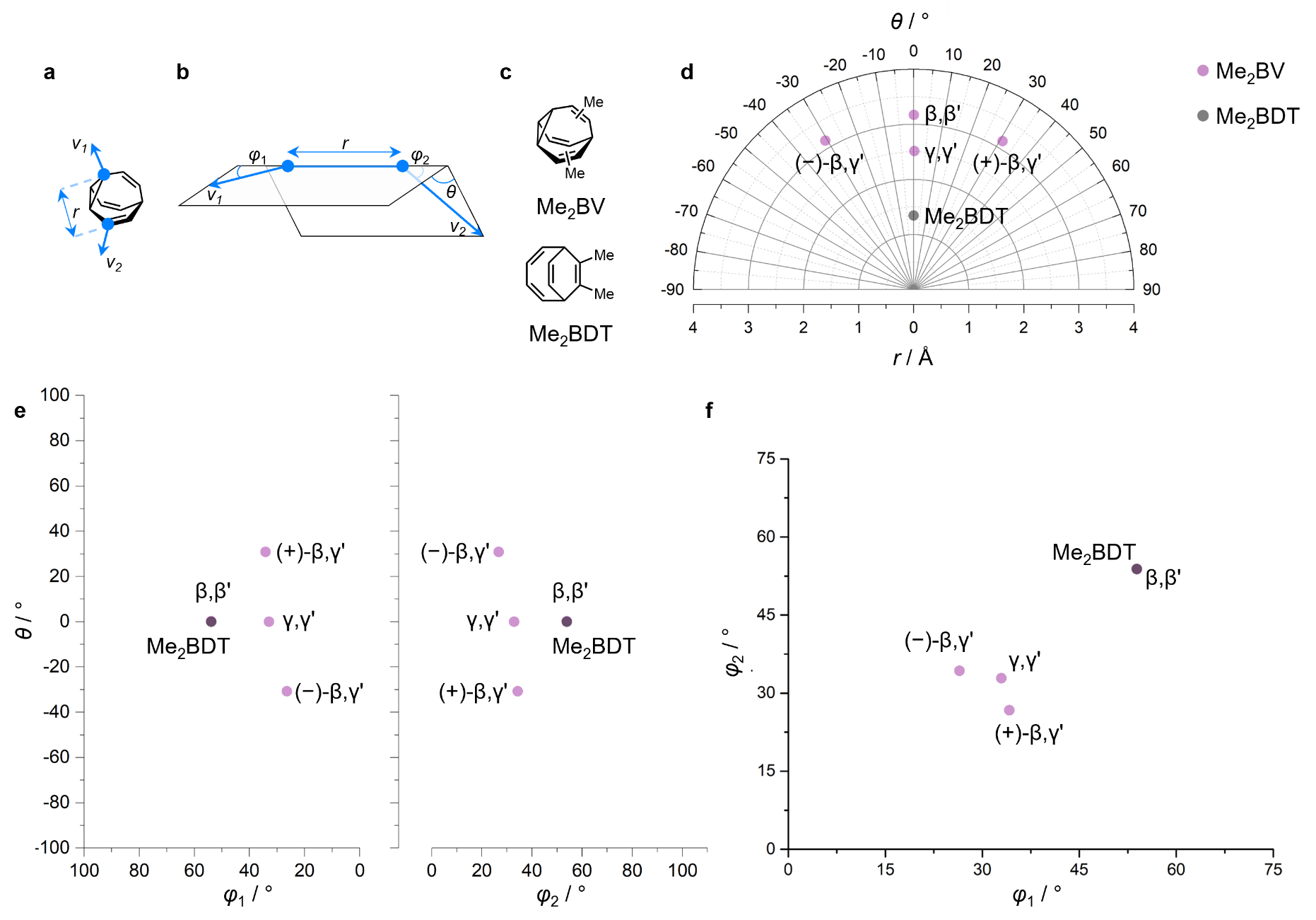


**Figure S44**. Exit vector plots for Me_2_BV and Me_2_BDT.

8. Polarised Microscopy

**
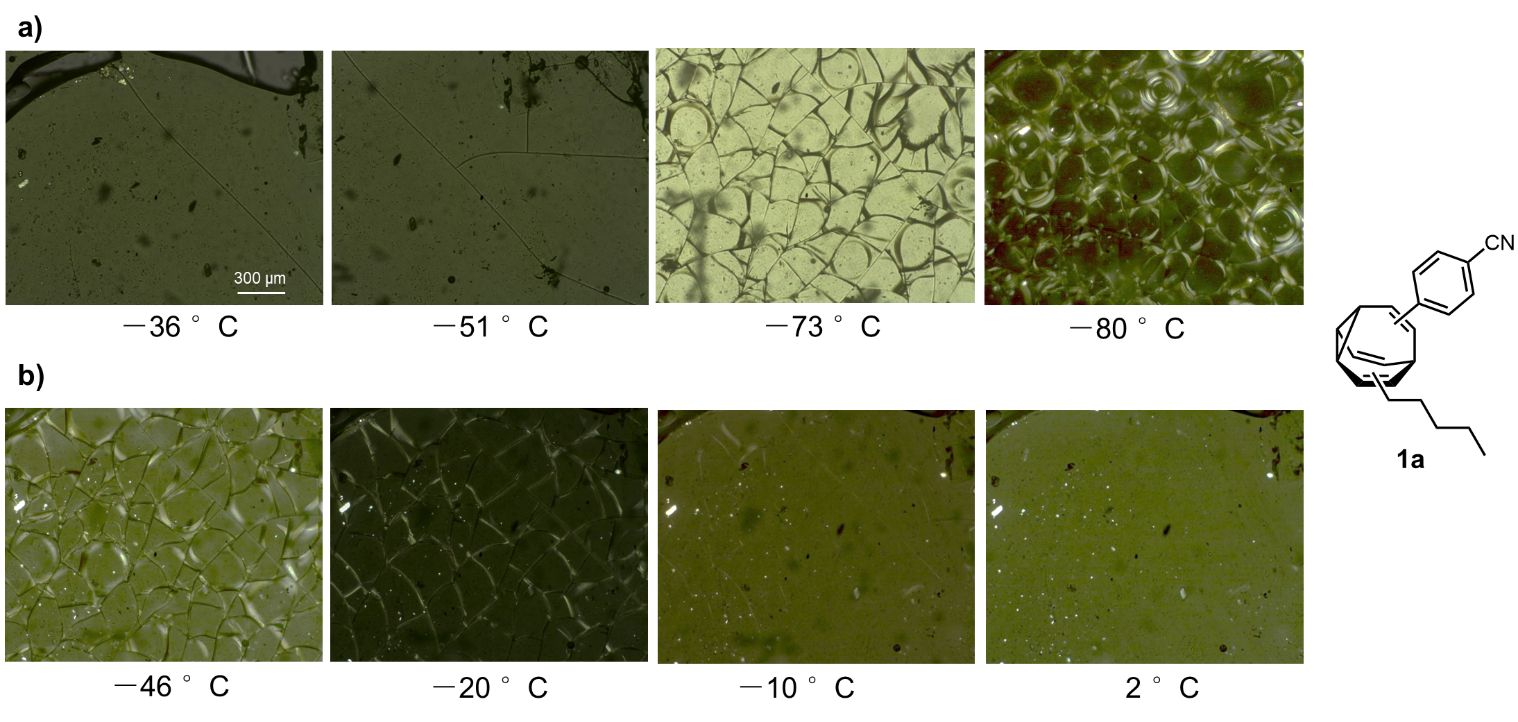
**

**Figure S45**. Polarised microscopy images of **1a** under variable temperature. a) cooling down; b) warming up.


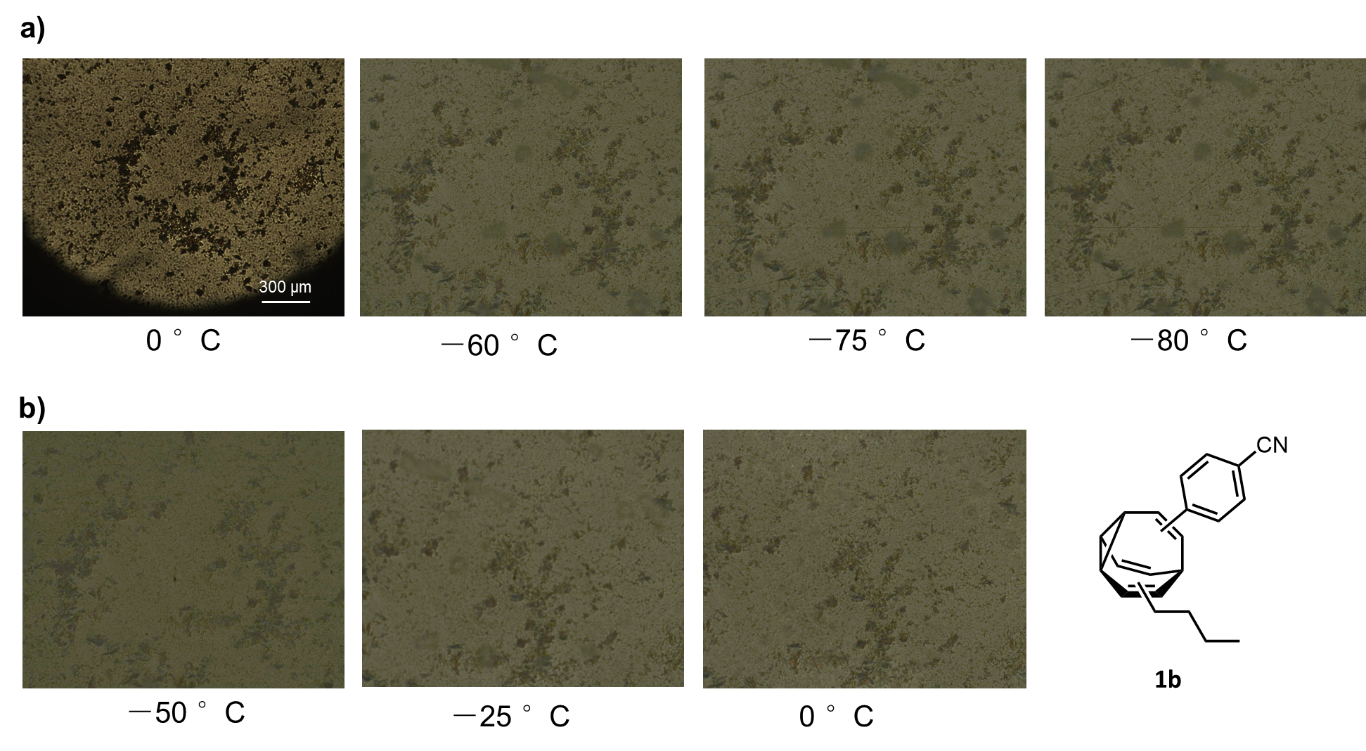


**Figure S46**. Polarised microscopy images of **1b** under variable temperature. a) cooling down; b) warming up.


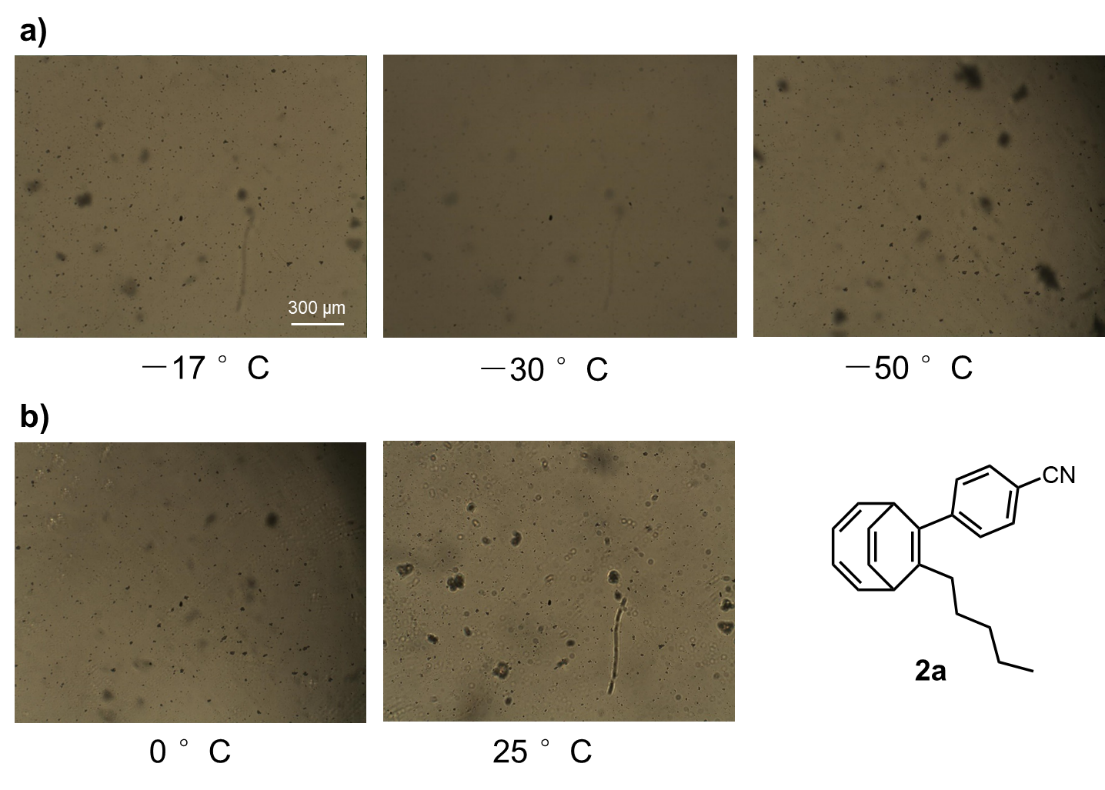


**Figure S47**. Polarised microscopy images of **2a** under variable temperature. a) cooling down; b) warming up.


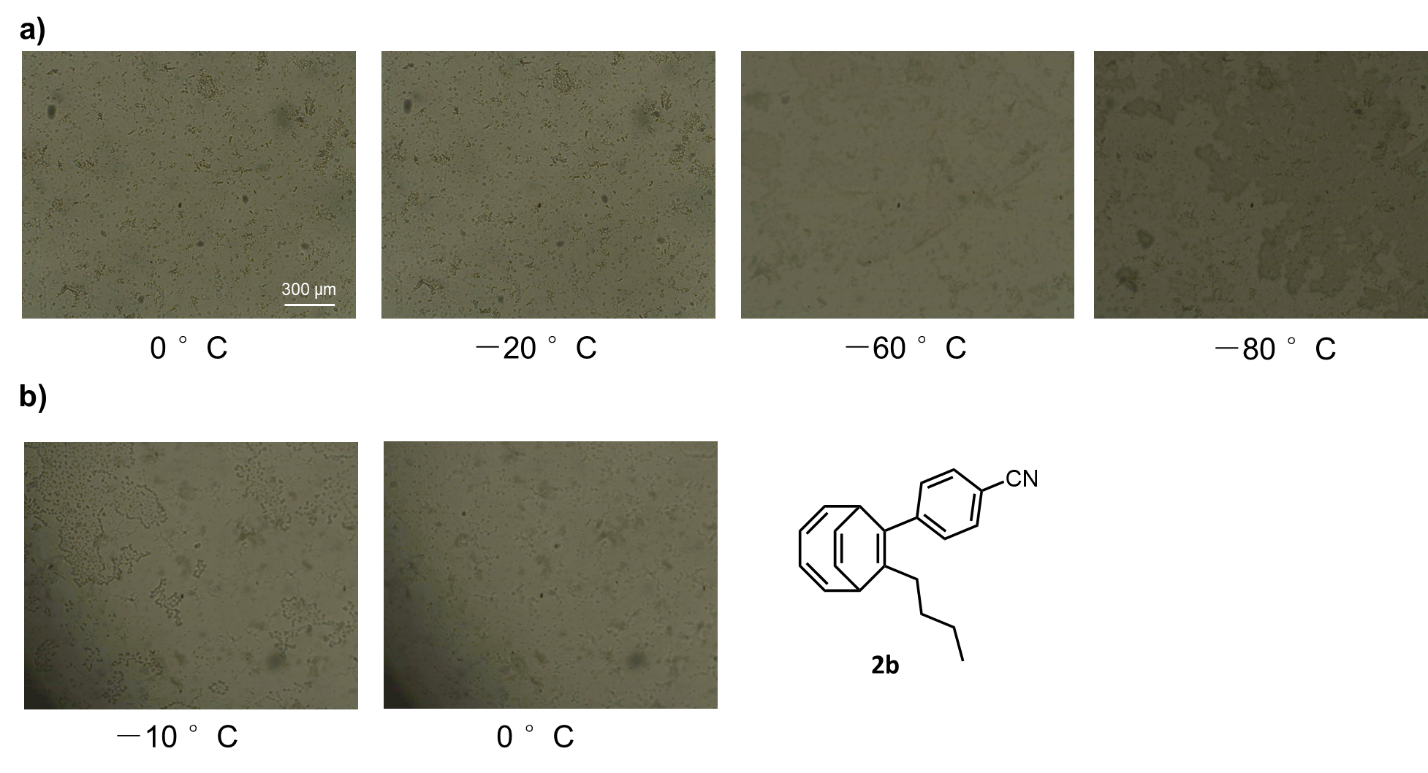


**Figure S48**. Polarised microscopy images of **2b** under variable temperature. a) cooling down; b) warming up.

A cycling experiment was also carried out, where a sample of **1a** was cooled to −85 °C to induce fracturing of the resultant glass. The sample was then warmed back to room temperature to allow the sample to anneal. This process was repeated a further two times, with images from the start and end of each cycle being captured (Figure S49).

**
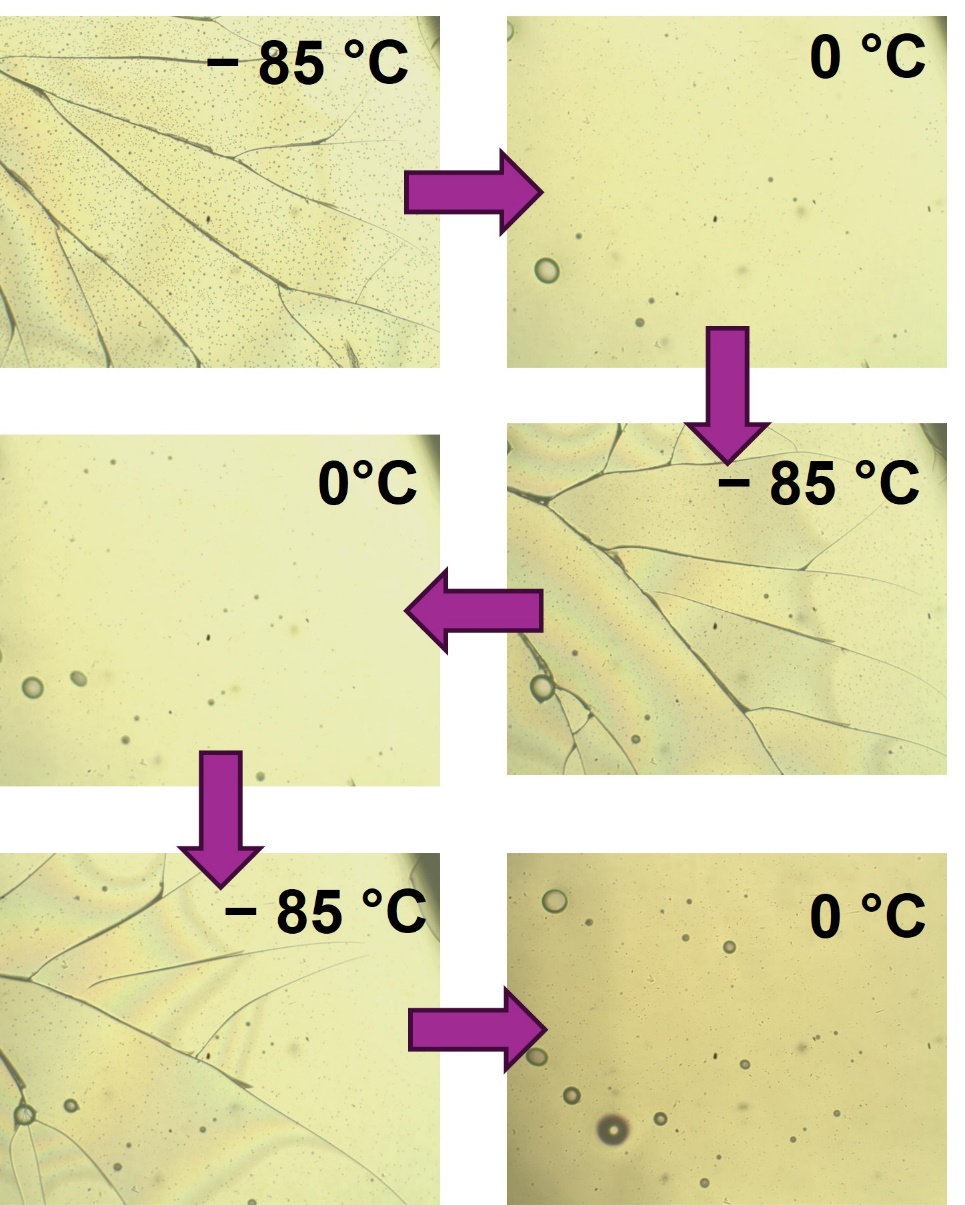
**

**Figure S49**. Three cycles of polarised microscopy images of fracturing and annealing of **1a**, beginning with an initial induced fracturing (top left) and followed by subsequent annealing and refracturing with repeated heating and cooling cycles.

9. Differential Scanning Calorimetry


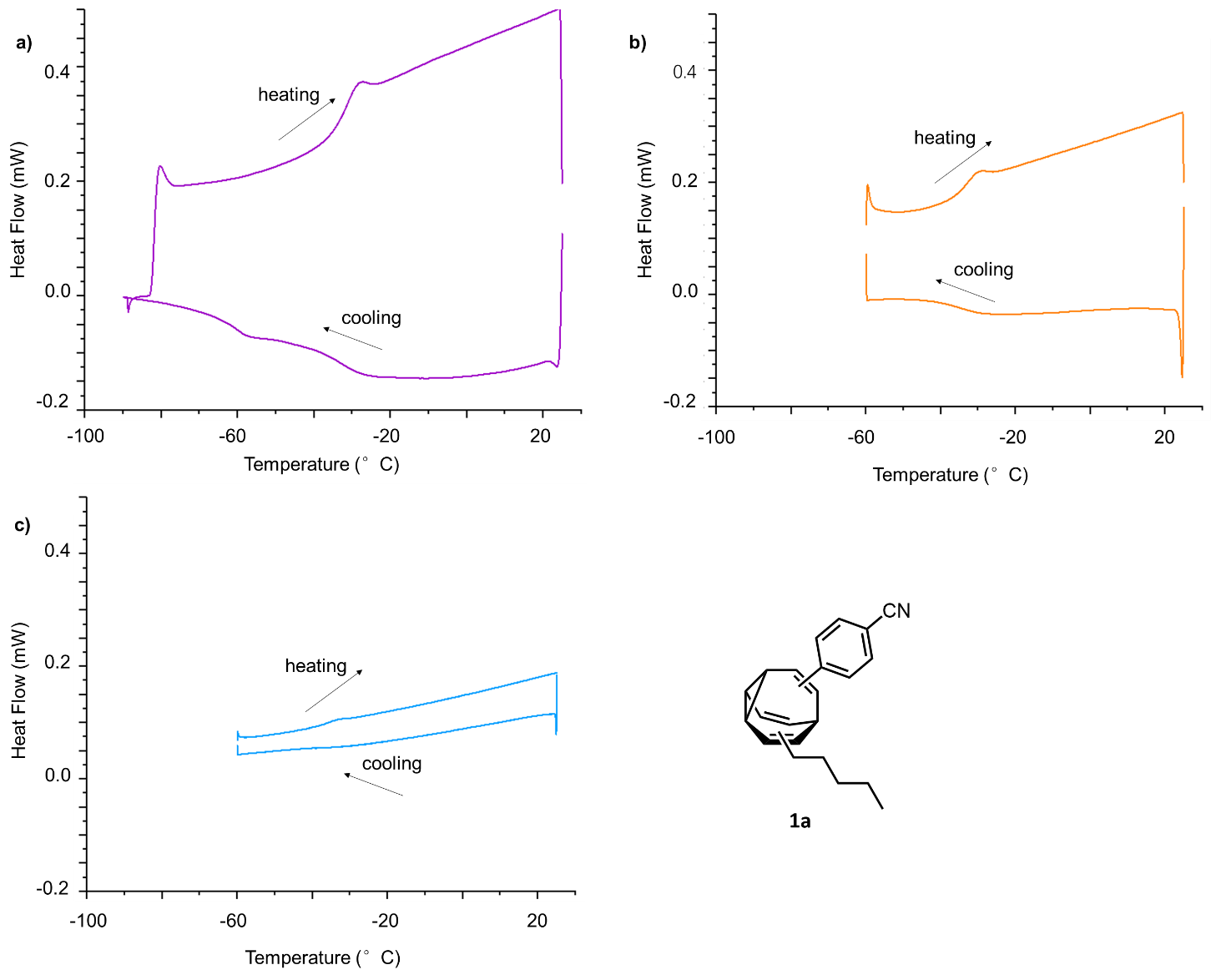


**Figure S50**. DSC diagrams of **1a** under different cooling and warming rates. a) 10 K / min; b) 5 K / min; c) 1 K / min.


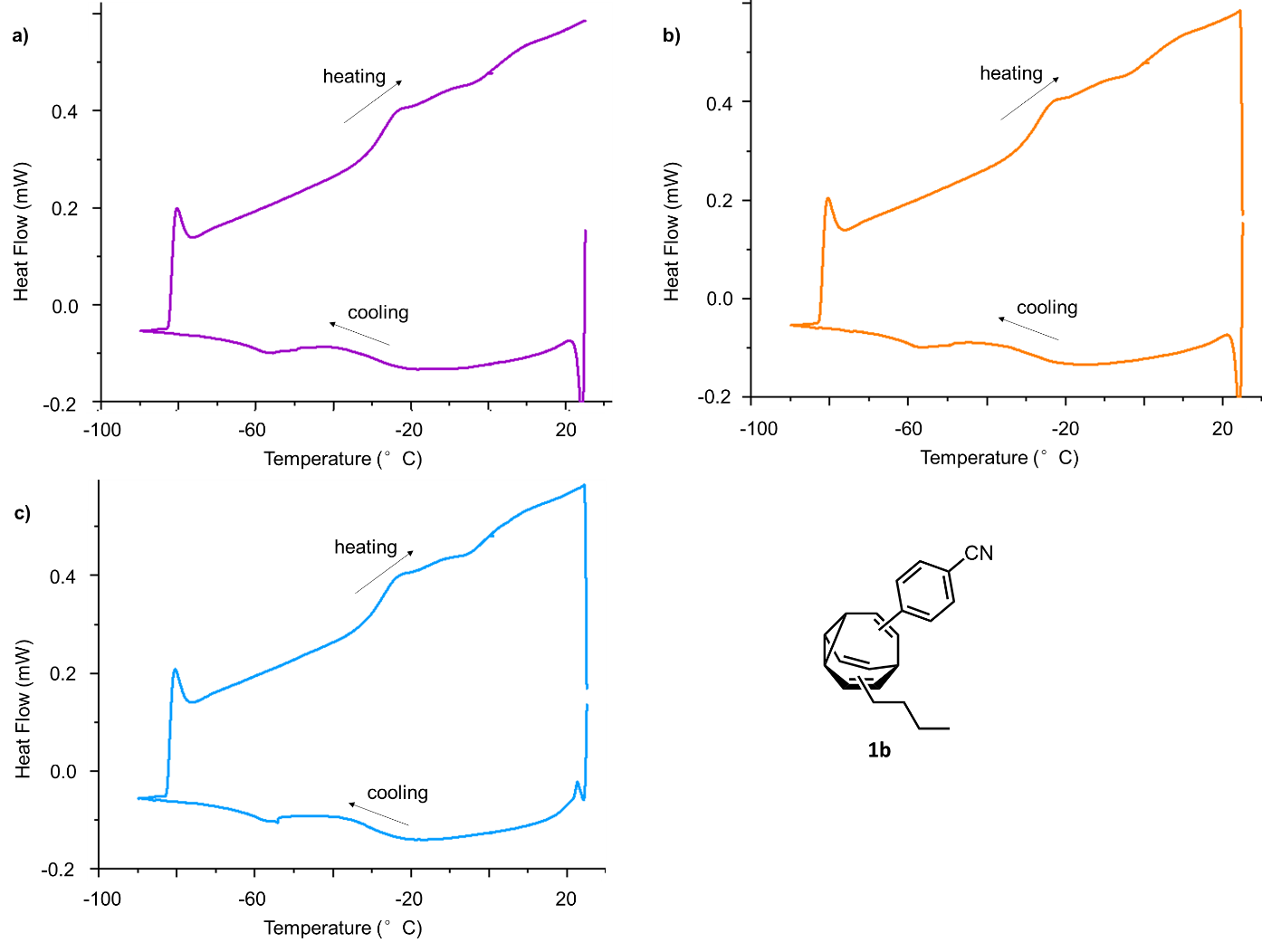


**Figure S51**. DSC diagrams of **1b** under different cooling and warming rates. a) 10 K / min; b) 5 K / min; c) 1 K / min.


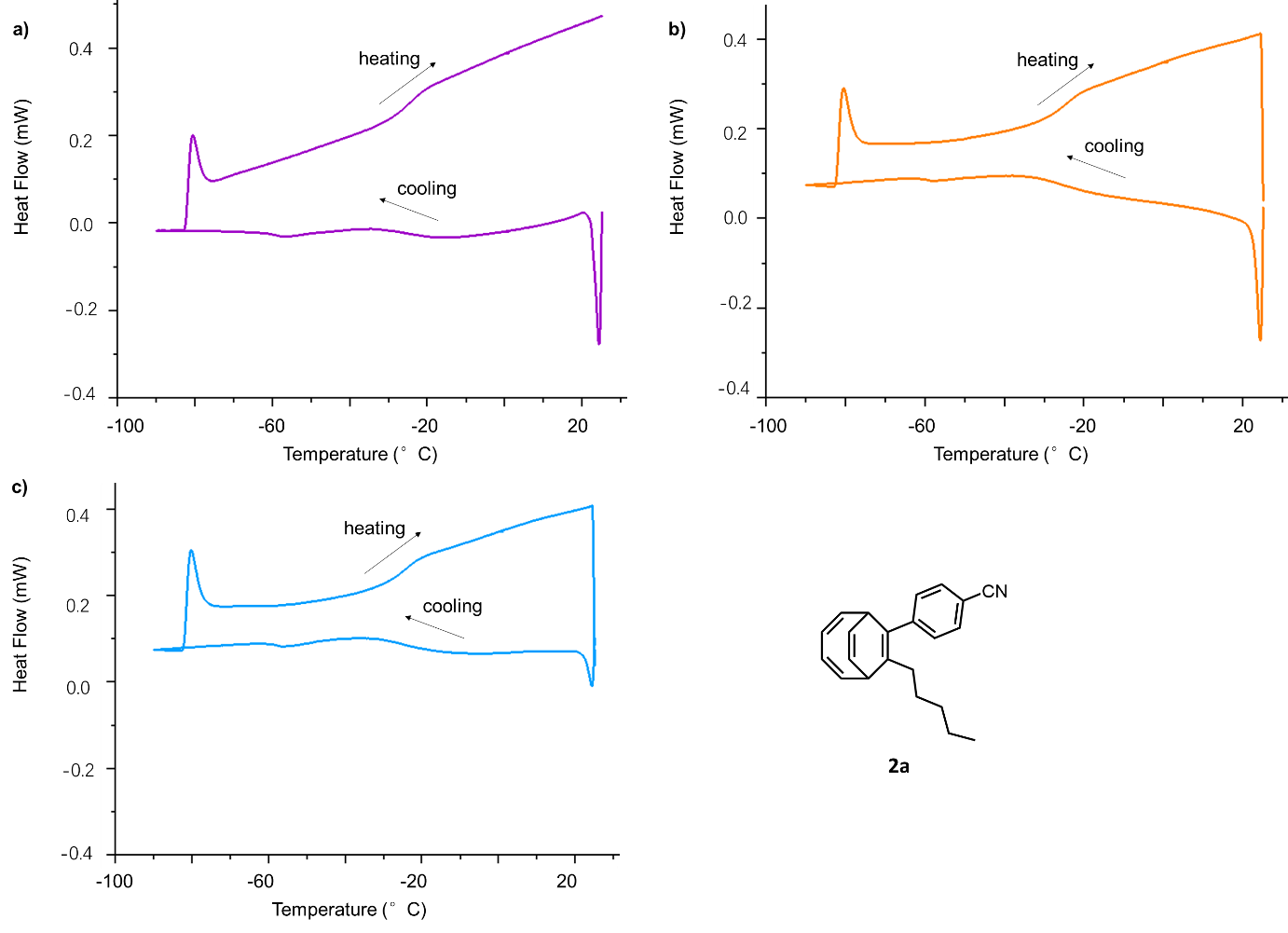


**Figure S52**. DSC diagrams of **2a** under different cooling and warming rates. a) 10 K / min; b) 5 K / min; c) 1 K / min.


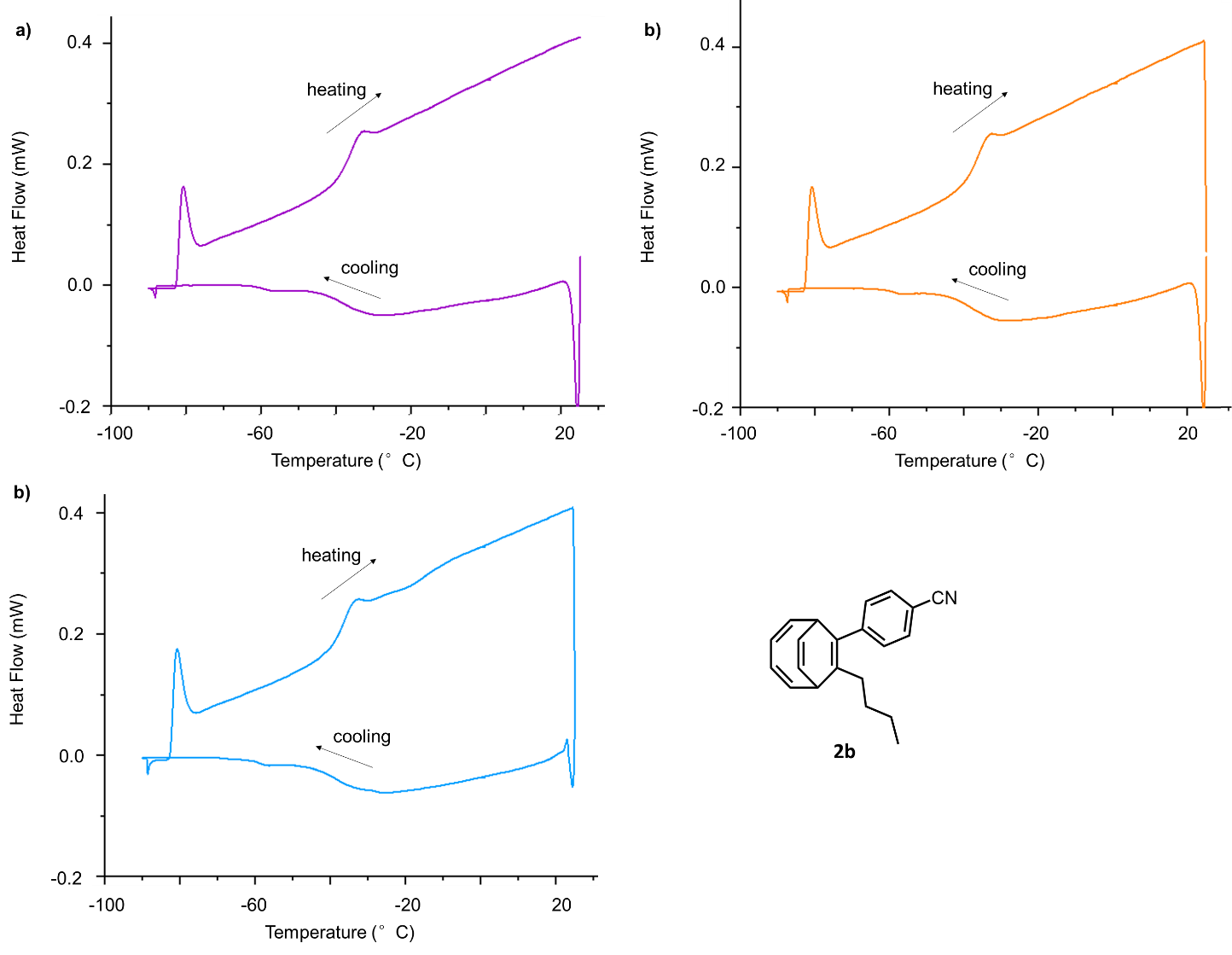


**Figure S53**. DSC diagrams of **2b** under different cooling and warming rates. a) 10 K / min; b) 5 K / min; c) 1 K / min.

**Table S2**. *T*_g_ ranges and heat capacity changes of **1** and **2**.

|  | Cooling | | | Heating | | |
| --- | --- | --- | --- | --- | --- | --- |
|  | *T*_g_ onset (°C) | *T*_g_ midpoint (°C) | ΔCp (J/g·K) | *T*_g_ onset (°C) | *T*_g_ midpoint (°C) | ΔCp (J/g·K) |
| **1a**  (10 K / min) | –33.1 | –32.8 | 0.454 | –20.0 | –32.8 | 0.293 |
| **1a**  (5 K / min) | –36.0 | –33.9 | 0.421 | –29.8 | –35.4 | 0.320 |
| **1a**  (1 K / min) | –37.4 | –37.0 | 0.369 | –33.7 | –34.3 | 0.369 |
| **1b**  (10 K / min) | –32.0 | –27.8 | 0.601 | –18.4 | –29.8 | 0.257 |
| **1b**  (5 K / min) | –31.6 | –27.6 | 0.586 | –19.5 | –29.8 | 0.211 |
| **1b**  (1 K / min) | –31.4 | –27.7 | 0.565 | –18.4 | –30.0 | 0.264 |
| **2a**  (10 K / min) | –29.2 | –25.5 | 0.596 | –16.7 | –23.8 | 0.242 |
| **2a**  (5 K / min) | –29.5 | –25.7 | 0.541 | –20.9 | –27.9 | 0.283 |
| **2a**  (1 K / min) | –29.2 | –26.3 | 0.539 | –22.1 | –26.4 | 0.149 |
| **2b**  (10 K / min) | –39.3 | –37.1 | 0.450 | –26.4 | –37.7 | 0.266 |
| **2b**  (5 K / min) | –39.2 | –37.0 | 0.458 | –27.8 | –38.3 | 0.297 |
| **2b**  (1 K / min) | –39.2 | –37.0 | 0.440 | –26.1 | –38.1 | 0.266 |

10. X-ray Diffraction

An X-ray diffraction experiment was used to verify the amorphous nature of the glassy state of **1a** by loading the sample onto a crystallography loop and attempting to diffract incident X-rays using a similar setup as would be used for X-ray crystallography (Figure S54a). The sample was cooled from room temperature and then irradiated with X-rays at various temperatures above and below the glass transition of the sample. The lack of a significant X-ray diffraction pattern is evident in Figure S54b.


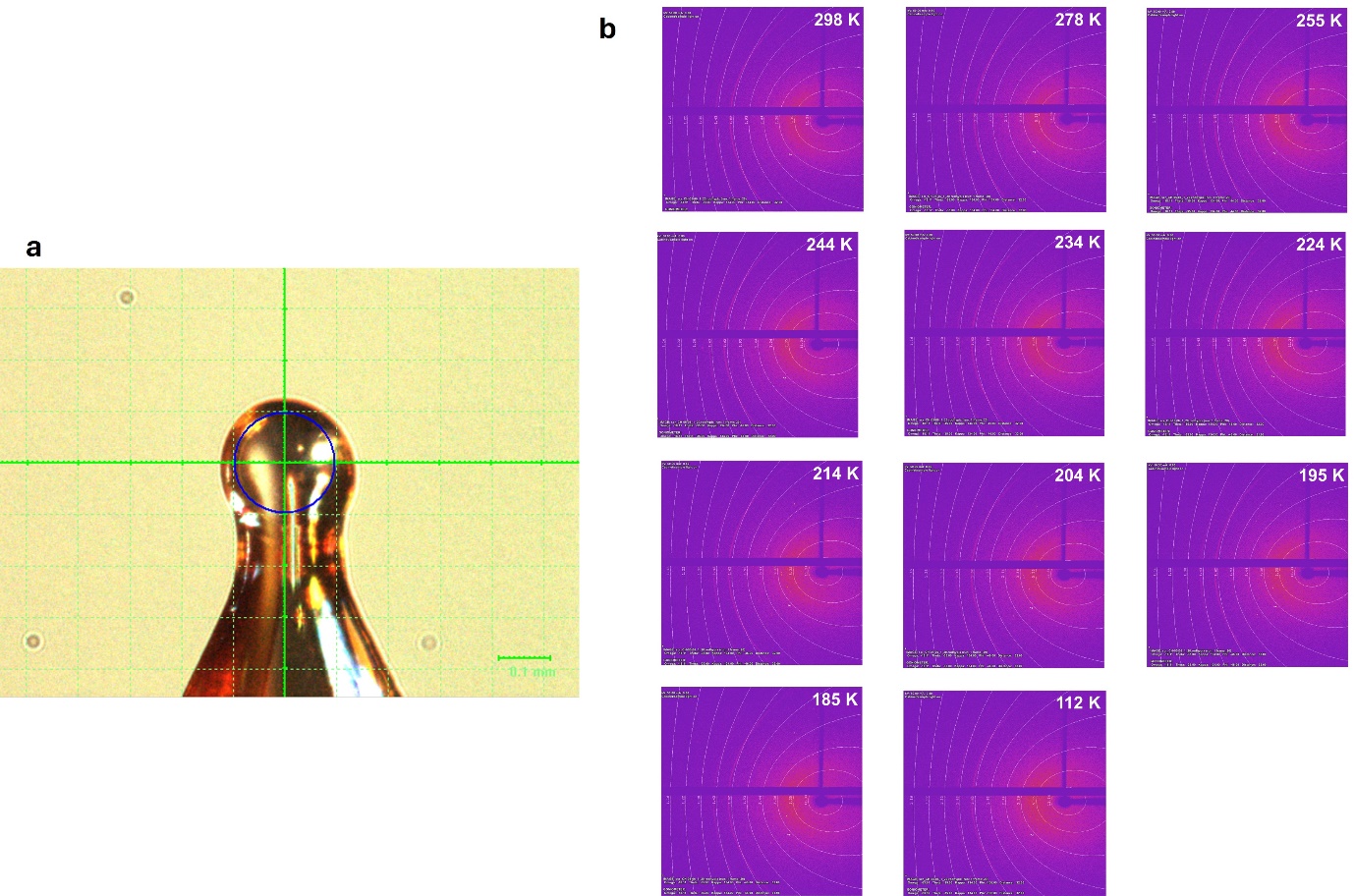


**Figure S54**. (a) A loop loaded with **1a** (b) was cooled from room temperature to its solid (glass) state at finally down to 112 K with periodic irradiation with X-rays giving rise to no X-ray diffraction, consistent with its amorphous structure.
